# Winter distribution of zooplankton and ichthyoplankton assemblages in the North Sea and the English Channel

**DOI:** 10.1371/journal.pone.0308803

**Published:** 2024-10-07

**Authors:** Carolin Julie Neven, Carolina Giraldo, Raphaël Girardin, Alain Lefebvre, Sébastien Lefebvre, Christophe Loots, Cédric Leo Meunier, Paul Marchal

**Affiliations:** 1 Ifremer, Channel and North Sea Fisheries Research Unit, Boulogne-sur-Mer, France; 2 Ifremer, Laboratoire Environment Ressources, Boulogne-sur-Mer, France; 3 Univ. Littoral Côte d’Opale, CNRS, IRD, UMR 8187 Laboratoire d’Océanologie et de Géosciences, Univ. Lille, Station Marine de Wimereux, Lille, France; 4 Alfred-Wegener-Institut Helmholtz-Zentrum für Polar- und Meeresforschung, Biologische Anstalt Helgoland, Helgoland, Germany; Central University of South Bihar, INDIA

## Abstract

Although zooplankton were extensively studied in the North Sea, knowledge about winter zooplankton assemblages is still scarce, despite potential influence of zooplankton overwintering stocks on seasonal plankton succession and productivity. Furthermore, several economically and ecologically important fish species reproduce during winter contributing to the zooplankton community as passive members (eggs) or predators (larvae). To elucidate on winter zooplankton distribution, abundance and composition in the Southern North Sea and Eastern English Channel, we defined assemblages based on mesozoo- and ichthyoplankton data sampled between January and February 2008 using fuzzy-clustering and indicator species. Mesozoo- and ichthyoplankton (eggs+larvae) were integrated in a common analysis by using a spatial grid adapted to the datasets and defined by means of a geostatistical method developed in agronomics. Potential environmental drivers of assemblage distribution were evaluated by means of GLMM and comparison with data from 2022 facilitated insight about the inter-annual representativeness of the assemblages. Five zooplankton assemblages were found varying with regard to total zooplankton abundance, dominant and indicator taxa. Spatial variability of abiotic (dissolved nutrients, salinity, depth, temperature, organic matter in suspension, chlorophyll a), biotic variables (phyto- and microplankton composition), water masses and fish spawning grounds were revealed as potential drivers of assemblage distribution. Assemblages off the Rhine-Scheldt estuary and in the German Bight harbored the biggest zooplankton overwintering stocks that might influence the grazing pressure on phytoplankton spring production. Assemblages off the Rhine-Scheldt estuary and covering the English Channel and the Southern Bight were found to be of high importance for herring and plaice larvae. Although further analyses suggested inter-annual representativeness of the assemblages found (2008 vs 2022), the assessment of further years would be necessary to account for potential inter-annual variability. Future studies could profit from the assessment of microzooplankton facilitating insight in fish larvae feeding potential and zooplankton overwintering strategies.

## Introduction

Zooplankton play a pivotal role in marine ecosystems with regard to the trophic web, biogeochemical cycles and the biological carbon pump [1]. The group of zooplankton is highly diverse considering size (pico to mega), taxonomy, life history traits and behaviors [2] allowing for complex interactions within the ecosystem. In temperate regions, the annual abundance of zooplankton organisms follows a seasonal cycle. This cycle starts with a phytoplankton spring bloom facilitating an increase of zooplankton abundance and biomass characterized by a succession from herbivorous to predatory taxa [2, 3]. Depending on hydrological conditions and community structure, a second abundance peak may occur in autumn facilitated by the phytoplankton autumn bloom growing on remineralized nutrients and terminating the productive period [2]. But what happens outside of these periods? How and where do zooplankton organisms survive or live during low primary production and winter conditions? Some insight is coming from studies on overwintering strategies and seasonality of marine copepods [4–9]. Whereas the copepod *Acartia clausi* was found to undergo a reproductive dormancy probably regulated by intrinsic factors as egg production increased irrespective of environmental conditions, *Temora longicornis* and *Centropages typicus* can reproduce throughout winter [9] albeit reproduction rate may be driven by prey availability and temperature, respectively [9, 10]. Some species like *T*. *longicornis* seem to display a mixed strategy consisting of hibernal (winter) reproduction and production of resting eggs [4, 5, 9]. Entire annual presence was also observed for congeners of *Temora spp*. in other regions [11]. Further overwintering strategies of zooplankton are diapause, seasonal vertical migration, building of energy storage and reduced growth and metabolic rate, for instance [12]. Zooplankton overwintering stocks are of importance as their size and distribution were found to influence year-to-year variations in the general abundance and distribution of zooplankton in the North Sea [13, 14] functioning like a seed [8] ready to flourish as soon as conditions are adequate. Evidence suggests that depending on the size and composition of the zooplankton overwintering stock the phytoplankton spring bloom might be exploited differently with regard to time, biomass, phytoplankton species and size classes [15]. As discussed by Nielsen and Richardson [16] small overwintering populations might leave a major part of the spring bloom unexploited. High initial copepod overwintering stocks by contrast might introduce top-down control earlier during the spring bloom development, prolonging the time span of nutrient availability for phytoplankton by remineralization. This might have further consequences for plankton succession and carbon sequestration [16–18]. Although overwintering strategies are complex and our understanding remains limited [9], spatial and temporal variation of zooplankton overwintering stocks can be expected. Temperature and prey availability are control mechanisms of overwintering. Thus, ocean warming [19–21] and observed changes in the phenology, composition and abundance of primary producers [20, 22, 23] might have taxa-specific influences on zooplankton overwintering [9]. This in turn, might have potential consequences for phytoplankton spring bloom succession [15, 24].

Zooplankton were extensively studied in the North Sea [e. g. 23–28], albeit winter populations have been less studied than spring-summer assemblages, despite growing acknowledgment of the importance of this period [14, 15, 28–31]. Furthermore, studies describing spatial distribution of single species or assemblages in detail remain scarce, spatially limited or describe the distribution of zooplankton at the end of the last century. Krause and Martens [32] and Krause et al. [33] assessed the zooplankton community of the entire North Sea sampled during winter 1987 and provided maps displaying the distribution of abundance and biomass for each taxon found. With regard to abundance three overall patterns of taxa distribution were revealed constituting the affiliation to Northern North Sea water, the central North Sea or coastal neritic areas [33]. With regard to biomass, centers of relatively high biomass were localized in the Southern North Sea (SNS) associated to eastern river deltas, off the British coast towards the Dogger Bank and in the Skagerrak region [32]. Van Ginderdeuren et al. [34] described the zooplankton assemblage in the Belgian part of the North Sea in winter 2009 as single neritic zooplankton assemblage dominated by *T*. *longicornis* and *A*. *clausi* with presence of oceanic species depended on Atlantic water inflow. In a recent study, Dudeck et al. [30] evaluated data sampled from 1991 to 2013 in the Eastern English Channel (EEC) and the Southern Bight to investigate temporal change in size and overall abundance of zooplankton considering potential spatial variation. Whereas zooplankton individual size displayed a decreasing trend, zooplankton abundance was found to increase with no difference among the four regions defined based on zooplankton congregations. None of these studies, however, defined and concurrently described the defined zooplankton assemblages and their spatial distribution in the Southern North Sea and Eastern English Channel (SNS-EEC), and ichthyoplankton (fish larvae and eggs) was never included comprehensively. Furthermore, although being a period of relatively low prey abundance [32], several fish species spawn in winter in the SNS-EEC [35–37]. The SNS-EEC harbor spawning sites, larval drifting routes and nursery areas and constitute therewith an important area for winter spawning fish, particularly for herring (*Clupea harengus*) and plaice (*Pleuronectes platessa*) [35, 36, 38]. The Downs herring spawning component, for instance, spawns in the EEC and Southern Bight of the North Sea from November until February [35, 39] and larvae hatching in the EEC are transported to eastern North Sea nursery grounds [38]. The Downs population has recovered after almost disappearing in the late 1970es, and it is now a major contributor to the overall North Sea herring recruitment [40, 41]. Fluctuations in Downs herring year class strength are driven by favorable environmental conditions combined with match-mismatch dynamics [42–44]. In particular, Downs herring larvae feed on overwintering plankton, and the lack of knowledge about the distribution and composition of these prey was mentioned as a major gap in understanding their survival [29].

A large scale approach aiming at the definition of plankton assemblages in the North Atlantic and its adjacent Seas using seasonally integrated data indicated the existence of several plankton assemblages in the SNS [45]. Although the data used in this study did not cover the Southern Bight and EEC and detailed information about the assemblages in the SNS-EEC remained limited, this study gives rise to the hypothesis that different zooplankton assemblages may exist in this area during winter. With the aim to shed light on the described knowledge gap, the present study intends to investigate the spatial distribution of winter zooplankton assemblages integrating mesozooplankton and ichthyoplankton in the SNS-EEC, and to discuss them with regard to potential environmental drivers. Understanding the distribution and composition of assemblages can be complex [46] as they might be influenced by several factors like abiotic condition (e.g. temperature, oxygen, depth) [47], predation [48, 49] and food availability [47]. Also food quality was shown to influence zooplankton abundance [50, 51]. The dissolved N/P ratio in the sea water was shown to reflect the phytoplankton quality in terms of prey for herbivorous zooplankton, as the N/P ratio in phytoplankton varies with the ratio of dissolved nutrients in surrounding sea water [52]. Phytoplankton displaying an N/P ratio close to the Redfield ratio of 16:1 were found to represent food of higher quality for copepod species [50]. These examples show that factors shaping assemblages are diverse and we aim to elucidate their relationships with the assemblages found by using a comprehensive set of potential environmental drivers.

Analyzing mesozooplankton and ichthyoplankton data concomitantly is methodologically challenging as these different zooplankton compartments are surveyed using different sampling schemes, resulting in datasets with distinct sampling resolution and coverage. In this study, we propose the use of a geostatistical method developed in agronomics [53] to define a common spatial grid adapted to the data and allowing for common analysis of those datasets. Using a clustering approach, we then defined zooplankton assemblages and assessed inter-annual variation of the spatial extent and composition of the assemblages found. As present knowledge about hibernal zooplankton assemblage composition and distribution is scarce, the outcome of the present study will allow to recognize potential future changes of zooplankton assemblage composition and distribution and help to better understand spring plankton succession/development in the context of climate change.

## Materials & methods

### Study area–SNS-EEC

**Fig 1:**
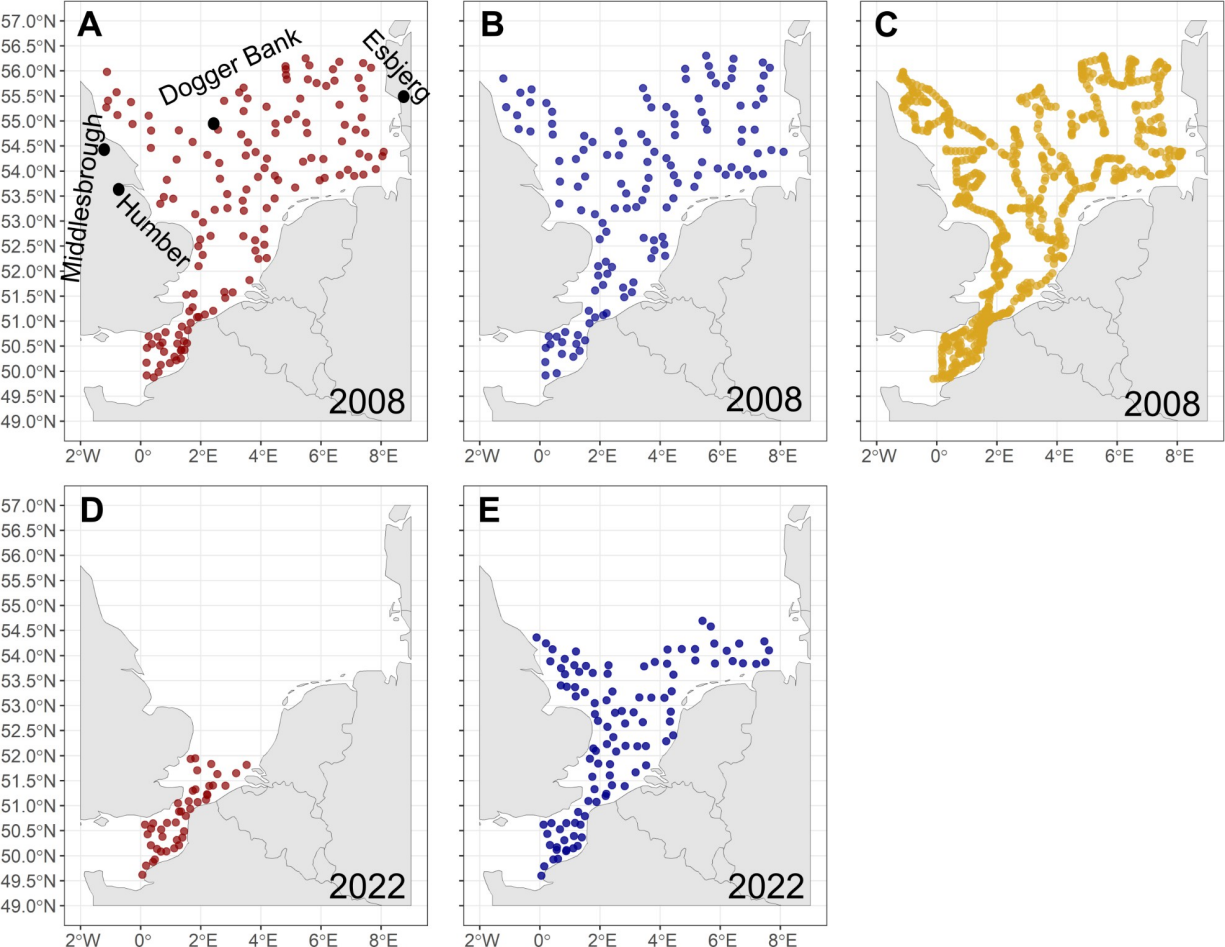
Sampling stations IBTS. (A) Mesozooplankton (IBTS 2008), black dots indicate locations delineating the geographical distinction between the Southern and Northern North Sea, furthermore the position of the sandbank Dogger Bank is indicated, (B) Fish larvae (IBTS 2008), (C) Fish eggs (IBTS 2008), (D) Mesozooplankton (IBTS 2022), (E) Fish larvae (IBTS 2022).

The North Sea is a European shelf sea being connected to the Atlantic Ocean in the North and via the English Channel in the South [54]. It can be divided in a northern and southern part due to its bathymetry, hydrology [54, 55] and ecology [26, 55] with a proposed border between Middlesbrough and Esbjerg [55] or the river Humber and Skagen (north of Esbjerg) [54] (Fig 1A).

The circulation and distribution of water masses are determining factors for the biology and ecology of the North Sea [54–56]. Svendsen et al. [57] described the accepted general mean pattern of North Sea circulation and distribution of water masses [54]. For a depiction of the circulation pattern see Krause et al. [54] Fig 5. Atlantic water enters the North Sea in the North by the Fair Isle current and over the eastern Shetland shelf area [58]. Only a small fraction of this Atlantic water protrudes further south as ’Scottish Coastal water’. Scottish Coastal water flowing along the British coast into the SNS is mixed with fresh water decreasing salinity to 34–34.75 and becoming ’Southern North Sea water’. This water mass prevails in the open SNS. Entering the North Sea via the Strait of Dover saline ’Channel water’ (salinity > 35) prevails in the Southern Bight. Flowing northwards along the east coast the Channel water is mixed with fresh water inputs and is called ’Continental Coastal water’ displaying a higher variability of salinity (31–34) and a lower mean salinity than the Southern North Sea water [54].

### Data

All zooplankton and most environmental data used in this study were sampled in January-February during the first quarter International Bottom Trawl Survey (IBTS) in 2008 and 2022, onboard of the French Thalassa Research Vessel from the French Oceanographic fleet. Additional details on sampling protocols are given below and are accessible using the survey’s DOI (2008: 10.17600/8040010, 2022: 10.17600/18001811).

In 2008 (27.01.2008 to 21.02.2008) data collection included taxa-specific abundance of zooplankton (mesozooplankton, ichthyoplankton (eggs, larvae), taxa-specific phyto- and microplankton abundance and environmental parameters (particulate organic matter (POM), chlorophyll a, nitrate (NO_3-_), nitrite (NO_2-_), ammonium (NH_4_^+^), phosphate (PO_4_^3-^), silicate (Si(OH)_4_)), temperature, salinity, depth) (Fig 1A–1C and S1 Fig). The ratio of nitrogen (sum of nitrite, nitrate and ammonium) and phosphate and the sum of nitrate and nitrite was calculated for further analysis. In 2022 (17.01.22–09.02.22) a more restricted spectrum of ecosystem and environmental parameters was assessed over a smaller spatial extent (Fig 1D and 1E). Data available for 2022 were mesozooplankton, fish larvae, temperature, salinity, depth and chlorophyll a concentration.

For the implementation of an anomaly analysis salinity and temperature data for the period 1998 to 2022 and chlorophyll a data available for the period 2008–2022 were downloaded from the ICES (https://www.ices.dk/data/dataset-collections/Pages/default.aspx) and Datras database (https://datras.ices.dk). Data of the herring larval index (1992–2022) were accessed from the 2023 report of the ICES working group HAWG [41].

#### Zooplankton taxa and abundance

*Mesozooplankton*. In 2008 and 2022, a total of respectively 142 and 43 mesozooplankton samples were taken using a WP2 bongonet (mesh size 200 μm) which was vertically hauled from 3 m above bottom to surface.

Species determination was assisted by a ZooScan [59, 60] (for further detail see supplementary S1 File). Since the ZooScan is able to detect organisms with an equivalent circular diameter of at least 300 μm [60], only the mesozooplankton size fraction >300 μm was retained in this study. Finally, specialists in zooplankton taxonomy validated ZooScan taxonomic classification output. In 2008, 46 groups of specimen were determined with the finest taxonomic determination level being genus (S1 Table) and multiples accounted for 6.6% of all pictures. In 2022, 62 taxa were found with the finest taxonomic level being species (S1 Table). Multiples accounted for 8.1% of all pictures. Taxonomic resolution was adapted to the resolution of the 2008 data set to facilitate inter-annual comparison reducing the number of taxa to 44.

Depth integrated mesozooplankton abundance was calculated as individuals per m^3^ as described in detail in the supplementary material (S2 File).

*Fish larvae*. In 2008 and 2022, a total of respectively 130 and 103 fish larvae samples were collected. Sampling took place after sunset and was realized using a MIK (Method Isaac Kid) with a mesh size of 500 μm. Sampling of fish larvae were carried out in accordance with relevant guidelines and regulation. Samples were analyzed for species abundance in the laboratory using a Stereo Microscope (Olympus SZX16 with a 7x – 115x zoom range). Calculation of abundance is described in the supplementary material (S3 File).

A total of eight taxonomic groups were found in 2008, of which four could be identified to species level (S1 Table). In 2022 a total of 14 taxonomic groups were found, including 11 species, one genus and two families (S2 Table). Herring larvae (*C*. *harengus*) were separated into three size classes to allow for spatial analysis of larvae with different prey composition. Class 6–12 mm represented the yolk sac and preflexion stage [61], class 13–20 mm represented a critical stage with regard to a change in prey selection [62, 63], and class 21–42 mm covered the postflexion stage [61]. No size specific data was available for the other taxonomic groups of fish larvae.

*Fish eggs*. In contrast to mesozooplankton and fish larvae samples, fish eggs were sampled continuously during the route of the research vessel. Seawater was pumped from three meters below sea surface by means of the Continuous Underway Fish Egg Sampler (CUFES) [64]. In total 861 samples were taken in 2008. Samples were stored in 1% formol. Taxonomic determination was realized using a Stereo Microscope (Olympus SZX16 with a 7x – 115x zoom range). Calculation of abundance is described in the supplementary material (S3 File).

In 2008, the eggs of five taxonomic groups (S1 Table) were collected of which two were identified to species level.

#### Phyto- and microplankton

In 2008, phyto- and microplankton taxa composition and taxa abundance were determined using inverse-microscopy. Calculation of abundance is described in the supplementary material (S3 File). To simplify later analysis taxa abundance of diatoms, dinoflagellates, flagellates, nanoflagellates, ciliates, chlorophytes and others was summed. The group of others contained the taxa *Phaeocystis globosa*, *Heterosigma spp*., *Eutreptiella spp*., Mediophyceae, Crysophyceae and Cryptophyceae. These groups represented the overall diversity of phyto- and microplankton and its spatial distribution sufficiently for the purpose of the present study.

### Data projection on an optimized grid

#### Estimation of optimal grid size

The optimal grid cell size was determined using the most comprehensive and spatially-extended 2008 dataset. As mesozooplankton, fish larvae and fish egg datasets differed in their sampling extent as in their sampling resolution a common sampling area and spatial grid was defined, building on an approach initially developed in agronomics [53] and to our best knowledge, for the first time applied to marine ecological data. The area of analysis (polygon) was restricted to the dataset covering the smallest sampling area (fish egg data set). Within the polygon 141 mesozooplankton samples, 129 fish larvae and 861 fish egg samples remained for further analysis. The optimal grid cell size (Lopt) was then defined as cell size that reduced the undesirable nugget variance (derived from a semi-variogram) to a minimum, whilst minimizing the resulting decrease in the informative variance component of the spatial structure [53, 65] (S4 File). After having defined taxon-specific-Lopt (79.9–123.3 km) (S3 Fig), we sought a compromise value for which Lopt was close to the maximum taxon-specific-Lopt of all taxa, and for which the number of empty grid cells was kept to a minimum. For this purpose, the median value of the taxon-specific-Lopt was calculated resulting in an Lopt of 91.58 km producing a maximal information content per cell that differed by less than 1.7% from the information content obtained using taxon-specific-Lopt. The minimum number of sampling stations per cell was one for mesozooplankton and fish larvae and two for the egg dataset (S4 Fig). One cell of central position did not contain sampling stations and thus was excluded from further analyses. A detailed description of the process is provided in the supplementary material (S4 File). The Lopt of 91.58 km was subsequently applied to project information from both the 2008 and 2022 datasets.

All analyses were performed in R version 4.2.1 using the package gstat [66] and sp [67] for the geostatistical analyses and the package raster [68] for definition of empty cells per grid cell size.

#### Planktonic abundance and environmental parameters per grid cell

A flow chart of the following steps (S5 Fig) and more detailed information with regard to method selection is given in the supplementary material (S5–S7 Files).

The abundance (x) of all zooplankton taxa was transformed with the log(x+1) function. This transformation allowed to downscale the high variability among the abundances of the different taxa [69]. Using a bootstrap with the number of iterations set to 10000, the mean abundance per grid cell was calculated for log-transformed and raw data of each taxon. The mean of log-transformed data was used for clustering whereas the mean derived from raw data was used for the determination of indicator species and to investigate the relation to environmental drivers later on (see below). The mean of non-transformed and log(x+1) transformed environmental parameters and phyto-microplankton abundance was calculated using the same method. Using the bootstrap method reduced the bias resulting from a differing number of sampling or measuring stations per grid cell [70].

Summing the mean abundance of all taxa collected in a grid cell, the total mean zooplankton (mesozooplankton and ichthyoplankton) abundance and the total mean phyto-microplankton abundance per grid cell were calculated.

### Determining assemblages by means of fuzzy clustering

In order to define assemblages based on the most important taxa with regard to abundance [71], whilst considering the ecological meaning of rarer specimens, taxa were separated into dominant and secondary. A taxon was considered dominant when its relative abundance was higher than 0.5%, and secondary otherwise [72]. Dominant taxa were used for clustering. Secondary taxa were included in the calculation of indicator species [73] (described later in this paragraph). Although dominant, the broad zooplankton taxonomic groups Copepoda, Calanoida, and Crustacea nauplius were excluded from the clustering analysis (S5 File).

The grid cell mean of mesozooplankton and ichthyoplankton taxa (calculated on log-transformed data) were transformed to relative abundance by means of the Hellinger transformation [74, 75], to enhance the joint analysis of the different data sets [75]. Furthermore, it allowed assigning the same weight to mesozooplankton and ichthyoplankton in the clustering analysis by rendering the contribution of abundant and rare species to the distance matrix similar [74, 76, 77]. Hellinger distance [74] was chosen as distance metric in this study.

After a pilot-study testing several clustering methods (S5 Fig and S6 File) the fuzzy c-means clustering method was chosen as most appropriate for the present data. In fuzzy clustering each cell is affiliated to each cluster and strength of affiliation to a cluster is expressed by the membership value. The sum of per-cell membership values over all clusters equals 1. To assess the coherence of the assemblage, maps displaying the maximum membership value per cell were produced indicating cells of strong affiliation (high membership value) and therefore coherent clusters and cells of weak affiliation (low membership value) and therefore less coherent regions (S7 Fig). Fuzzy clustering was implemented with a membership exponent of 1.2 using the function fanny () from the cluster package [74, 78].

The number of clusters (k) was determined using three statistical methods (Silhouette widths, Mantel correlation [74], Kelly-Gardner-Sutcliffe penalty function [79]) and by taking into account the existence of indicator taxa, the spatial pattern of membership values and ecological reasoning. The optimal number of clusters was set to 5. The choice of k is further detailed and justified in the supplementary material (S7 File). Indicator taxa were determined using the IndVal method [73]. All indicator taxa considered, had an indicator value higher than 0.25 [72, 73].

Analysis was conducted using the package labdsv [80] and a p-value of 0.05 adjusted after Benjamini and Hochberg [81].

Clustering analysis was conducted in R version 4.1.2 using the packages cluster [78], vegan [82] and maptree [83] and results were mapped by means of the packages sf [84], sp [67], EchoR [85] and ggplot2 [86].

To characterize the assemblages, the mean abundance of the taxa per assemblage was calculated using the non-transformed grid cell mean and visualized using barplots. Additionally, Principal Component Analysis (PCA) was applied to log(x+1) and Hellinger transformed grid cell means of zooplankton abundance using the package FactoMineR. PCA allows to reduce the dimensionality of a dataset. The position of variables with regard to the new dimensions created by the PCA, reveals patterns and relationships between variables and individuals. Thereby each new dimension explains a percentual proportion of the variance in the dataset and the most explaining ones are kept for interpretation.

### Environmental drivers of taxa distribution

A generalized linear mixed model (GLMM) was used to evaluate potential drivers of zooplankton distribution and therewith assemblage composition. If available, the relation of one mesozooplanktonic and one ichthyoplanktonic (fish larvae) indicator taxon per assemblage with potential environmental drivers was tested. To synthesize the environmental parameters measured, two PCAs were applied on log-transformed, centered and scaled grid cell means of abiotic (temperature, salinity, depth, sum of nitrate and nitrite, ammonium, phosphate, silicate, POM, chlorophyll a) on one side and to hellinger-transformed grid cell means of biotic parameters (abundance of 7 phyto-microplankton groups), on the other side. The N/P ratio was used as supplementary variable due to its correlation to nitrogen and phosphate. The dimensions (principal components) of the PCAs explaining the majority of the variance were used as explanatory variables in the GLMM model. As principal components are orthogonal to each other, no problems of correlation between the dimensions of the same PCA in the model can be encountered. Potential correlations between the principal components originating from the two different PCAs were tested. To consider the spatial component, assemblages (cluster) were integrated as random effect. GLMMs were applied to untransformed grid cell means of zooplankton taxa abundance. Starting with the most complete model, the parameters were reduced in a step-by-step procedure and the most parsimonious model was chosen with regard to the smallest AIC and significant Anova. The fulfillment of model assumptions was verified using the DHARMa package. To simplify interpretation the most parsimonious model with variables coded as independent was chosen if AIC, Anova and the fulfillment of assumptions allowed to do so. Models were run using the package glmmTMB [87]. Depending on data distribution, a “gamma” or “tweedie” distribution was used.

The most complete model coding variables as interactions was constructed as follows:

glmmTMB(taxon_abundance ~ Dim.1abiotic* Dim.1biotic + Dim.2abiotic* Dim.1biotic + (1|cluster),

family = distrib)

with distrib equal to tweedie () or Gamma(link =“log”).

This model tested also the separate effect of the independent variables.

### Inter-annual comparison

The distribution and composition of the assemblages found in 2008 were compared to data sampled in 2022 as zooplankton data coming from the same campaign were available for these two years. As the sampling extent of mesozooplankton sampled in 2022 was the smallest among data sets (Fig 1D), this sampling extent defined the area (polygon) serving for the inter-annual comparison. The clustering approach described above was applied to the spatially restricted data set of 2008. A k of 2 was found the most appropriate choice (S18 Fig). 2008 clustering (Fig 5) was then applied to calculate the mean abundance of the dominant (in terms of dominant and secondary) taxa sampled in 2022 per cluster. Inter-annual differences between the total abundance of mesozooplankton and fish larvae respectively and of the most dominant taxa per cluster were assessed by means of GLMM. Spatial autocorrelation was considered by integrating coordinates as random effect. Models were run using the glmmTMB package in R. Depending on data distribution a “log-normal” or “tweedie” distribution was used. The model applied to single taxa and total abundance was constructed as follows:

model <- glmmTBM(taxon_abundance ~ cluster + cluster:year + exp(coordinates + 0|year), family = tweedie())

or

model <- glmmTBM(log(taxon_abundance) ~ cluster + cluster:year + exp(coordinates + 0|year), family = gaussian())

With the aim to place the two years examined in a broader inter-annual context, an anomaly analysis with data of salinity, temperature (1998–2022), chlorophyll a concentration (2008–2022) and the herring larvae density index (1992–2022) sampled during the first quarter IBTS was conducted. Due to data availability, the region between 49°N and 55°N was used for the anomaly analysis.

## Results

### PCA on potential abiotic and biotic drivers

**Fig 2:**
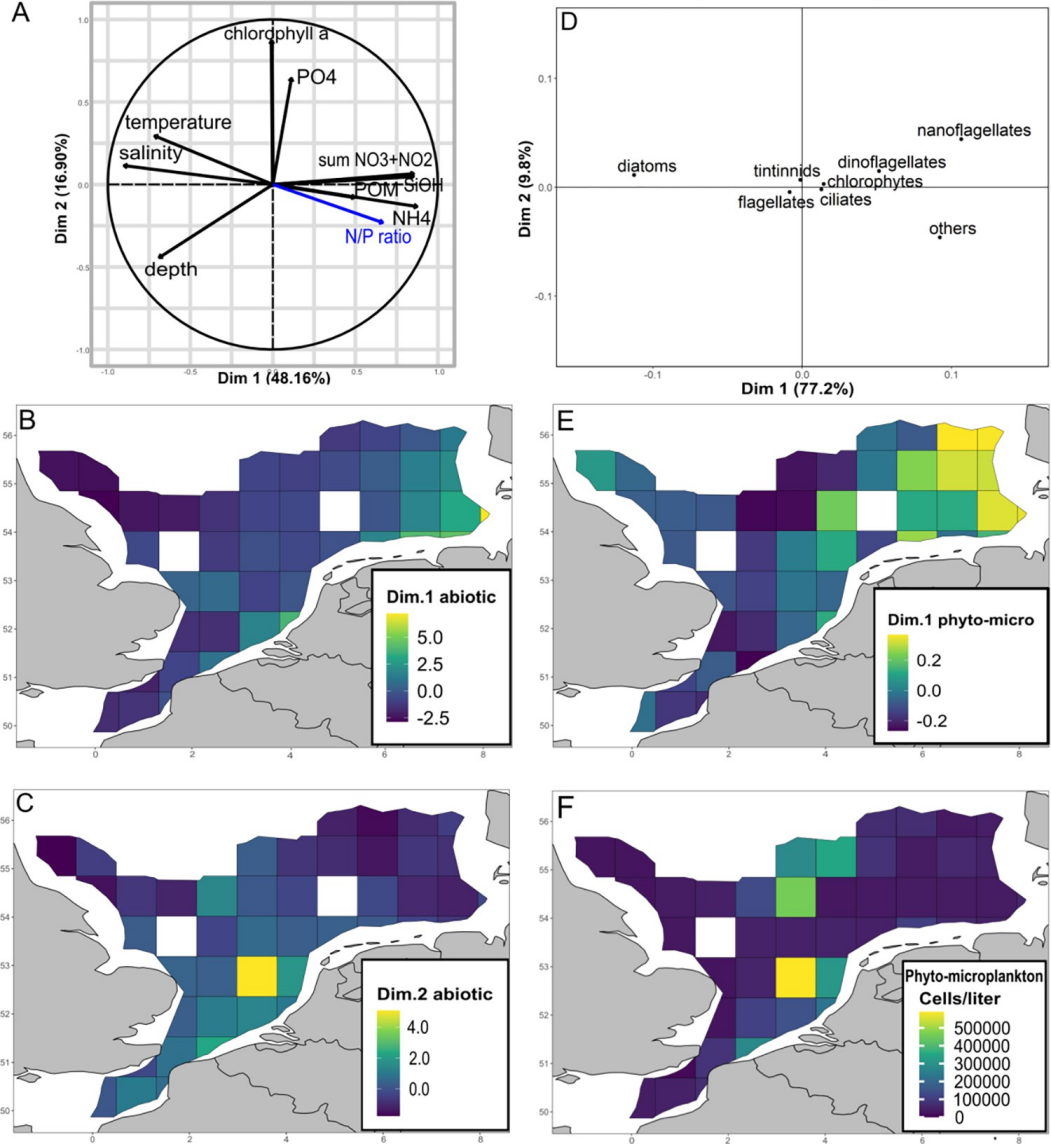
Environmental drivers. (A) Abiotic parameters displayed in a two-dimensional space constituted by the first and second dimensions of the PCA. (B) Values of coordinates of first dimension of the PCA on abiotic parameters per grid cell. The more positive a value the higher the concentration of nutrients, and the lower temperature, depth and salinity and vice versa. (C) Values of coordinates of second dimension of the PCA on abiotic parameters per grid cell. (D) Phyto- and microplankton displayed in a two-dimensional space constituted by the first and second dimensions of the PCA. (E) Values of coordinates of first dimension of the PCA on phyto-microplankton per grid cell. High values indicate high proportion of nanoflagellates and others (*Phaeocystis globosa*, *Heterosigma spp*., *Eutreptiella spp*., Mediophyceae, Crysophyceae and Cryptophyceae), low values indicate high importance of diatoms. (F) Total abundance of phyto- and microplankton per grid cell.

The first two dimensions of the PCA applied to abiotic variables (Fig 2A–2C) explained 64% of the total variance (48% and 16%, respectively). The first dimension (A1) represented an inverse relationship between dissolved nitrogen (sum of nitrate and nitrate, ammonium), silicate and POM on the positive side and temperature, depth and salinity on the negative side. Thus, a grid cell displaying a high value of dimension one was characterized by high POM, nitrogen and silicate concentration and low temperature, depth and salinity (Fig 2B). The second dimension (A2) represented chlorophyll a and phosphate concentration (Fig 2A), with higher values representing cells with high phosphate and chlorophyll a concentration (Fig 2C). The first dimension of the PCA applied to phyto- and microzooplankton (B1) explained 77% of the variance (Fig 2D). It represented the abundance and distribution of diatoms, nanoflagellates and the group of others (*Phaeocystis globosa*, *Heterosigma spp*., *Eutreptiella spp*., Mediophyceae, Crysophyceae and Cryptophyceae) with higher values indicating abundance of nanoflagellates and others whereas lower values indicated importance of diatoms (Fig 2E).

### Characterization of the assemblages found

The 2008 dataset was the most extensive in terms of spatial extent, observations and environmental variables available, and we first provide detailed results of the assemblages found with that dataset, their environmental conditions and the overall pattern. The assemblages found in 2008 and 2022 with a reduced spatial extent are compared subsequently.

**Fig 3:**
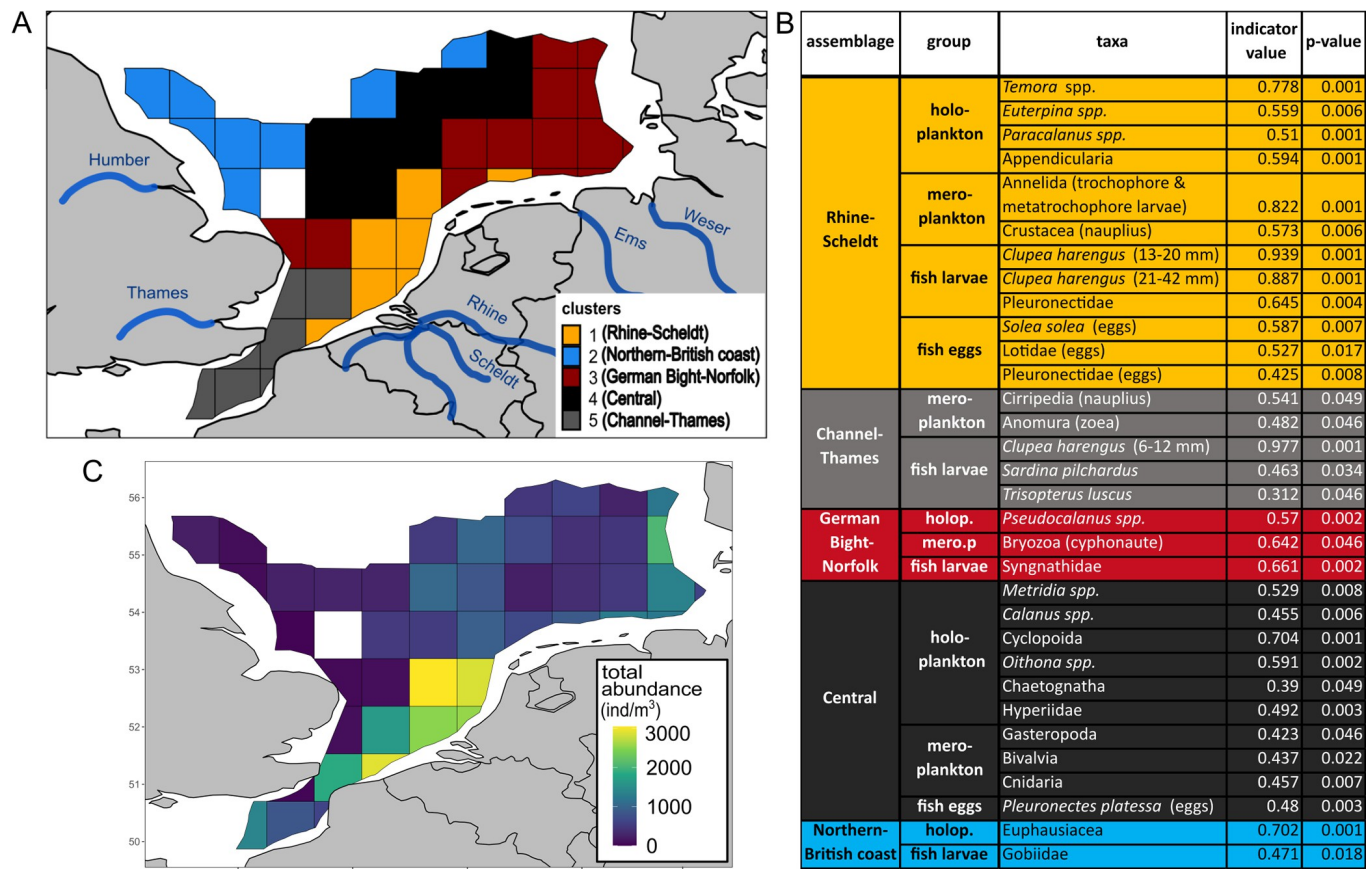
Assemblages and total zooplankton abundance. (A) Clusters/assemblages based on mesozooplankton and ichthyoplankton sampled during the International Bottom Trawl survey in January and February 2008. (B) Indicator species per cluster/assemblage with respective indicator value and adjusted p-value. (C) Total mean abundance of zooplankton (meso- and ichthyoplankton) per grid cell.

A total of five clusters was determined in the SNS-EEC representing five zooplankton assemblages (mesozoo- and ichthyoplankton) within the study area (Fig 3A). These assemblages were named as follows: cluster 1 will be referred to as ‘Rhine-Scheldt assemblage’, cluster 2 as ‘Northern-British coast assemblage’, cluster 3 as ‘German Bight-Norfolk assemblage’, cluster 4 as ‘Central assemblage’ and cluster 5 as ‘Channel-Thames assemblage’. As each assemblage was associated to a certain region the name of the assemblages was used together with the term region when referring to the location of the respective assemblage.

#### Zooplanktonic and environmental profiles per assemblage

**Fig 4:**
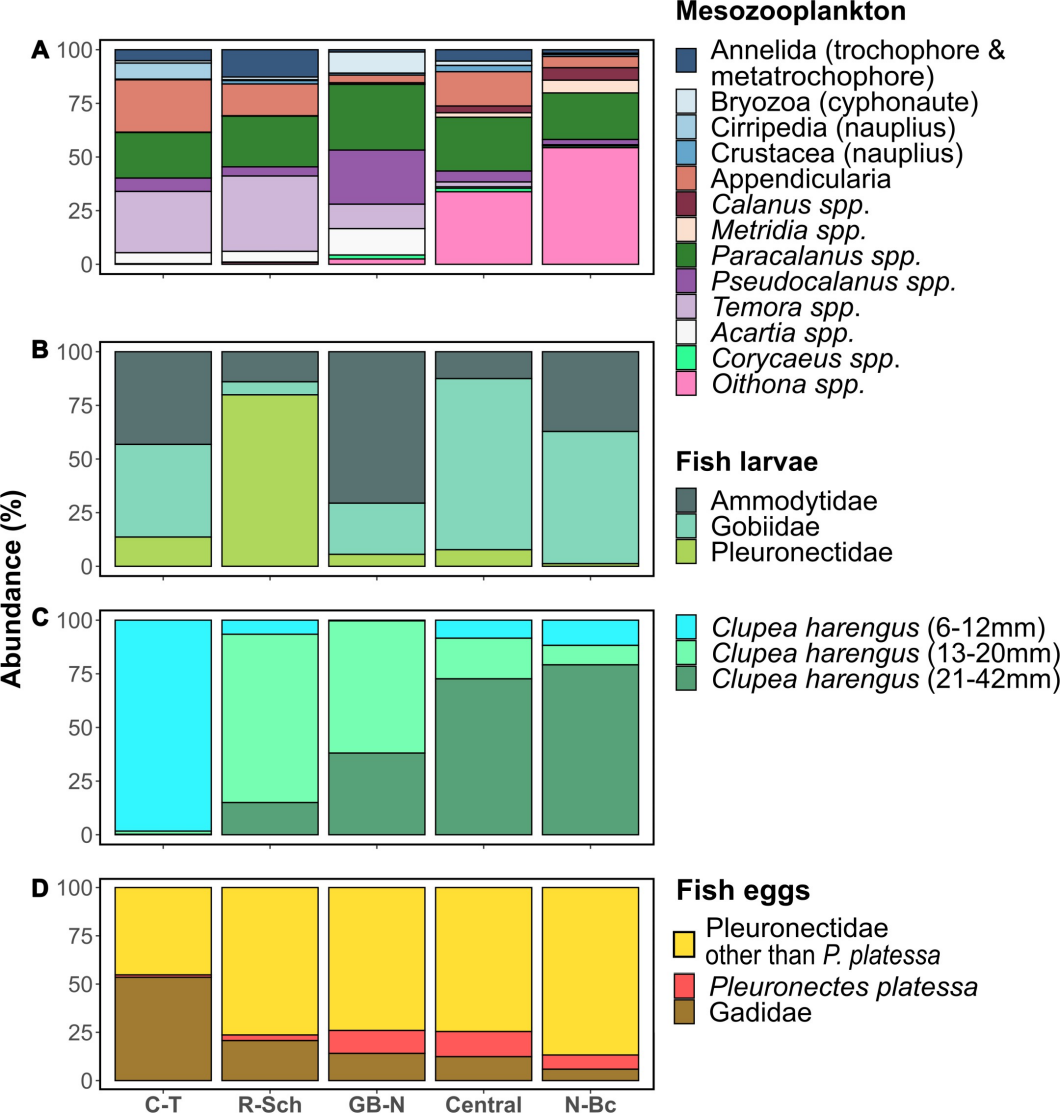
Proportional taxa composition per assemblage. Each bar corresponds to an assemblage: C-T: Channel-Thames assemblage. R-Sch: Rhein-Scheldt assemblage; GB-N: German Bight-Norfolk assemblage; Central: Central assemblage; N-Bc: Northern-British coast assemblage. (A) Mesozooplankton; (B) Fish larvae other than herring; (C) Herring larvae; (D) Fish eggs.

A detailed description summarizing the taxa-specific characteristics of the assemblages and their prevailing abiotic conditions is provided in the supplementary material (S8 File). In the following the overall pattern of characteristics within and among the assemblages will be described (Figs 3 and 4 and S10 and S11 Figs).

For an overall comparison of the zooplankton assemblages a PCA was applied to zooplankton data (S10 Fig). The first two dimensions explained 47.9% of the variance. PCA revealed an overall segregation between northern (Norther-British coast, Central) and southern assemblages (Rhine-Scheldt, Channel-Thames), with the Northern-British coast assemblage and the Central assemblage being located on the negative side of dimension one and the Channel-Thames assemblage and the Rhine-Scheldt assemblage on the positive side. This corresponded to the relative abundance of certain taxa with *Oithona spp*. Gobiidae larvae, *Metridia spp*. and *Calanus spp*. characterizing northern assemblages whereas southern assemblages were related to eggs of *Solea solea*, *Centropages spp*., small and medium sized herring larvae and *Temora spp*. (Fig 4 and S10 and S11 Figs). The German Bight-Norfolk assemblage was positioned on both sides of the first dimension what corresponded to the observation that *Pseudocalanus spp*. was part of the dominant taxa in contrast to the remaining assemblages (Fig 4 and S10 and S11 Figs).

The **Channel-Thames assemblage** was characterized by small herring larvae (6 and 12 mm), Cirripedia nauplius larvae, zoea larvae of the infra-order Anomura, larvae of *Sardina pilchardus* and *Trisopterus luscus* that were revealed as indicator taxa (Fig 3B). The larval assemblage was dominated by the smallest size class of herring larvae (97%) that displayed with a mean abundance of 508 individuals per 1000 m^3^ the highest abundance among assemblages.

The Channel-Thames region was characterized by negative values of dimension 1 of the abiotic PCA (A1) meaning warmer temperature, average salinity, low nitrogen, silicate and POM concentration and average values on dimension 2 (A2) i.e. phosphate and chlorophyll concentration (Fig 2). The phyto- and microplankton community was characterized by diatoms that accounted for 74% of total abundance (negative values on dimension 2 of the biotic PCA (B1)) (Fig 2 and S14–S16 Figs). The N/P ratio was with 20±7 slightly elevated with regard to the Redfield ratio.

The **Rhine-Scheldt assemblage** was characterized by 15 indicator taxa and covered the region with highest total zooplankton abundance (Fig 3C). Mesoplanktonic indicator taxa were trochophore and metatrochophore larvae of the phylum Annelida, Crustacea nauplii, *Temora spp*., Appendicularia, *Euterpina spp*., Calanoida, and *Paracalanus spp*.. Indicator taxa belonging to the ichthyoplankton were *C*. *harengus* size class 13–20 mm, *C*. *harengus* size class 21–42 mm, Pleuronectidae larvae, *Solea solea* eggs, Lotidae eggs and Pleuronectidae eggs (Fig 3C). The Rhine-Scheldt region was characterized by elevated abundance of phyto- and microplankton (Fig 2F) corresponding to positive values on A2 meaning elevated chlorophyll a concentration (Fig 2C). The phyto-microplankton assemblage was dominated by diatoms (58%) and the group of others (33%) (average value on B1) (Fig 2E and S17 Fig). Positive values on A1 represented elevated concentration of POM, nitrogen, phosphate and silicate and average temperature, salinity and depth (Fig 2A). The N/P ratio of 17±12 indicated rather balanced nutrient availability with regard to the Redfield ratio (S14 Fig).

The **German Bight-Norfolk assemblage** was characterized by cyphonaute larvae of the phylum Bryozoa, the copepod genus *Pseudocalanus* and fish larvae of the family Syngnathidae. The east of the German Bight-Norfolk assemblage covered a region of elevated zooplankton abundance (Fig 3C).

The German Bight-Norfolk region was characterized by positive values on A1 meaning cold temperature, shallow depth and low salinity as well as elevated concentrations of POM, nitrogen and silicate. Negative values on A2 represented low concentration of phosphate and chlorophyll a (Fig 2). A low concentration of phosphate (S14 Fig) and an elevated concentration of nitrogen resulted in an elevated N/P ratio of 21 ±11 (S14 Fig). The phyto- microplankton was dominated by diatoms (42%), others (28%) and nanoflagellates (17%) (positive values on B1) whereby nanoflagellates and dinoflagellates (10%) displayed the highest chair among assemblages (Fig 2 and S17 Fig).

Ten indicator taxa characterized the **Central assemblage**: Cyclopoida, *Oithona spp*., *Metridia spp*., Hyperiidae, Cnidaria, *Calanus spp*., Bivalvia, Gasteropoda and Chaetognatha, and one being *P*. *platessa* eggs (Fig 3B). The Central region displayed average depth, salinity, temperature, silicate and POM concentration (values around zero on A1). Values around zero on A2 mean average chlorophyll a and phosphate concentration (Fig 2). Nitrogen was low resulting in an N/P ratio of 6±3 (Fig 2 and S14 Fig). Total phyto-microplankton abundance was elevated (Fig 2F) and dominated by diatoms (81%) (negative value on B1) (Fig 2 and S17 Fig).

Two indicator taxa were revealed for the **Northern-British coast assemblage**, namely the order Euphausiacea and Gobiidae larvae (Fig 3B). Overall zooplankton abundance was low in the assemblage (Fig 3C).

With regard to environmental conditions, this region was characterized by deep depth, elevated salinity and warm temperature and low concentrations of POM, nitrogen, silicate (negative values on A1) and phyto-microplankton abundance (Fig 2). Negative values on A2 mean low chlorophyll a concentration (S14 Fig). N/P ratio was low (8±3). Diatoms dominated the phyto-microplankton assemblage as indicated by negative values on B1.

#### Drivers of species distribution

The two first dimensions of the PCA applied to abiotic parameters (A1, A2) and the first dimension of the PCA applied to the biotic variables (B1) were used as fixed explanatory variables in the GLMM. Biotic and abiotic drivers explaining taxa distribution differed between the indicator taxa tested (Table 1). Overall, A2 significantly explained the abundances of 6 out of the 10 taxa tested to a different extent whereas A1 and B1 contributed to the explanation of the distribution of 4 out of the 10 taxa tested. For 6 out of 10 taxa the random effect cluster was retained in the most appropriate model (Table 1). This means that an unexplained spatial gradient remained once environmental variables included. The most extreme case was Euphausiacea with a significant random cluster effect while no significant environmental drivers were evidenced. For the remaining taxa (*Temora spp*., *Pseudocalanus spp*., *Oithona spp*., Gobiidae larvae), the fixed variables alone were sufficient to explain the specificity of the taxa to the assemblages, meaning that any spatial effects were accounted for through the selected environmental variables.

**Table 1:** Outcomes of GLMM evaluating possible biotic and abiotic drivers of taxa distribution from two PCAs (dimensions = principal components). The n and y in the column cluster indicate if cluster was retained in the most appropriate model or not (n = no and y = yes). Significance: *** (P<0.001), ** (P<0.01), * (P<0.05),^.^ (P<0.1). N indicates the number of sampling stations used in the model. AIC (Akaike’s Information Criteria) was the parameter used for model selection with smaller AIC for the models of the same taxon indicating better explanation of variance. Estimates indicate significant and non-significant positive or negative correlation between a dimension and taxon abundance. Non-significant correlations were not displayed in the table.

Taxa	Intercept	Estimates								cluster	N	AIC
		Dim1-abiotic (A1)		Dim2-abiotic (A2)		Dim1-phyto (B1)		Dim1-abiotic (A1) x Dim1-phyto (B1)				
		+	-	+	-	+	-	+	-			
		NO_2-_+NO_3-,_ NH_4_^+^ Si(OH), POM	temperature, salinity, depth	chlorophyll a, PO_4_^3-^		nanoflagellates, others, dinoflagellates	diatoms					
*Temora spp*.	3.54	0.62***		1.17***						n	44	407
herring larvae (medium-sized)	0.71			0.34^.^						y	44	212
*Pseudocalanus spp*.	3.55	0.46***		0.38*		2.43**				n	44	408
Syngnathidae larvae	-3.42	0.15^.^			-0.89**					y	44	48
*Oithona spp*.	3.26		-0.43***		-1.06**					n	44	312
*Metridia spp*.	-1.09						-2.64^.^			y	44	154
Euphausiacea	-2.58									y	44	61
Gobiidae larvae	0.71				-0.74***		-3.52***			n	44	194
Cirripedia nauplius larvae	-0.33			1.27*						y	44	139
herring larvae (small-sized)	0.76					6.61*			-4,22*	y	44	203

The distribution of small herring larvae characterizing the Channel Thames region was explained by an interactive effect of A1 and B1 with a higher importance of B1 indicated by its revelation as significant (Table 1). This means that the abundance of small herring larvae was positively correlated to nanoflagellates and other phytoplankton that were the major drivers for this taxon but that in regions of low nanoflagellate and other phyto-microplankton abundance elevated nutrient and POM concentration still positively influenced small herring larvae abundance. The distribution of Cirripedia nauplius larvae and medium sized herring larvae were solely driven by chlorophyll a concentration (and possibly phosphates) in accordance with the elevated chlorophyll a concentration in the Channel-Thames and Rhine-Scheldt region. *Temora spp*. and *Pseudocalanus spp*. were further taxa positively correlated to chlorophyll a concentration (and possibly phosphates) but additional groups of variables explained their distribution and abundance. *Temora spp*. was additionally correlated to nutrient and POM concentration (A1) and *Pseudocalanus spp*. to nutrient and POM concentration (A1) and abundance of nanoflagellates and the group of other phyto-microplankton (B1) corresponding to the characteristics of the Rhine-Scheldt and the German Bight-Norfolk region, respectively. Syngnathidae larvae, *Oithona spp*. and Gobiidae larvae were negatively correlated to chlorophyll a and phosphate concentration (A2). The abundance and distribution of Syngnathidae larvae was further correlated to nitrogen and POM concentration (A1) and diatom abundance (B1) was revealed as further driver of Gobiidae larvae. The drivers of these two taxa corresponded to the conditions in the German Bight-Norfolk and Northern-British coast region. Beside low chlorophyll a and phosphorous concentration *Oithona spp*. were negatively correlated to A1 and thus further driven by elevated salinity, temperature, depth.

### Inter-annual comparison between zooplankton assemblages in relation to the SNS-EEC environment

#### Spatial extent and distribution of assemblages

**Fig 5:**
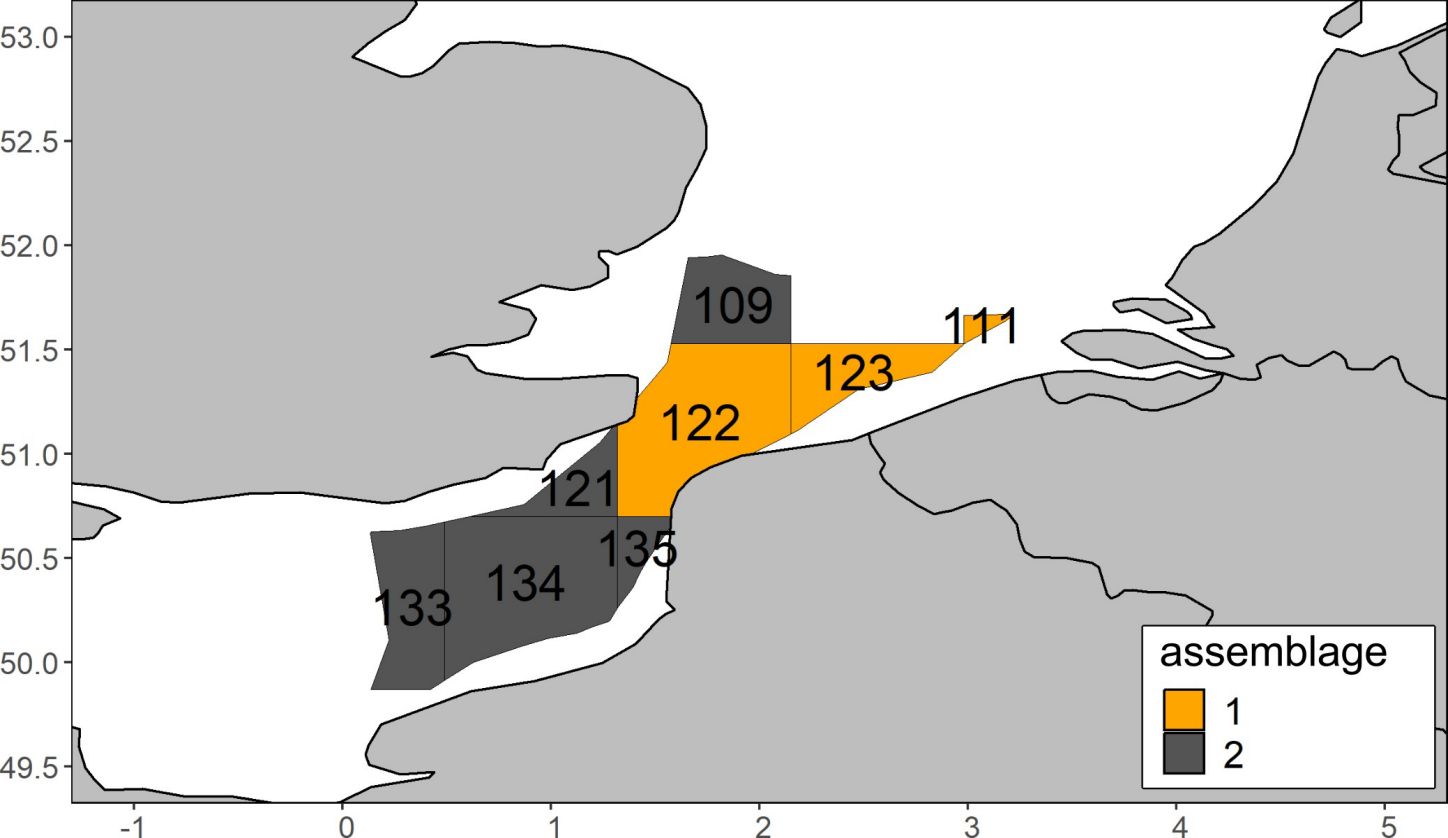
Zooplankton assemblages using the 2008 data and the small sampling extent.

Using a smaller spatial extent, clustering with data from 2008 resulted in regions slightly different (cell 122) from clustering utilizing the full spatial extent (Figs 3 and 5). As the distribution of the assemblages in 2008 nevertheless related to those found when using the full spatial extent, the same nomenclature will be applied with the orange assemblage corresponding to the Rhine-Scheldt and the grey assemblage corresponding to the Channel-Thames assemblage.

#### Inter-annual differences of assemblage composition and environmental condition

When comparing 2008 and 2022 zooplankton assemblages remained overall stable with regard to taxa composition, relative and absolute abundance.

**Fig 6:**
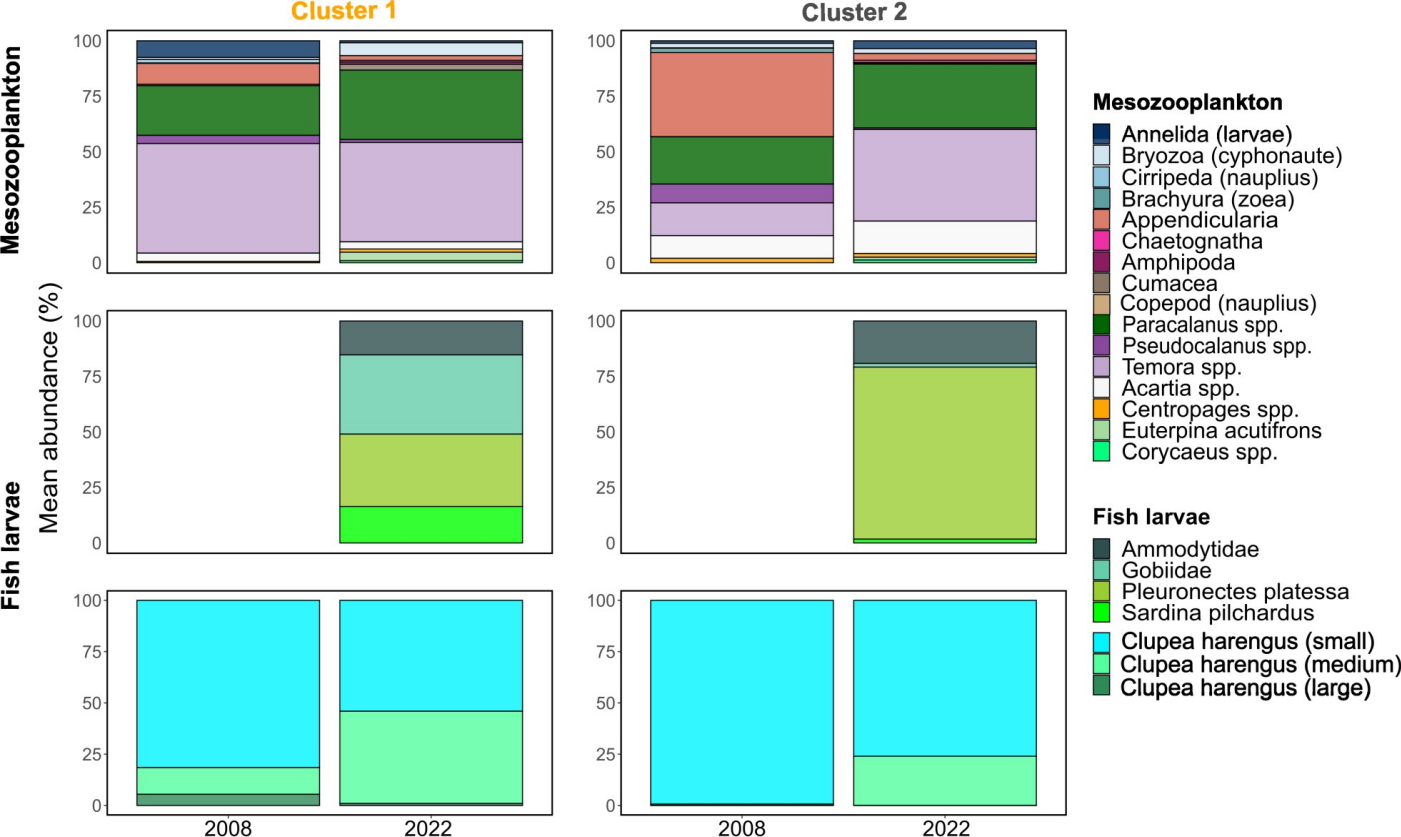
Comparison of relative community composition between 2008 and 2022 in the clustering output from 2008 (Fig 5). Fish larvae are displayed in two lines with the line positioned in the middle displaying all fish larvae other than herring larvae and the lowest line representing herring larvae of three different size classes.

With regard to relative abundance Appendicularia were not part of the dominant taxa in 2022 in the Channel-Thames region, in contrast to 2008 as relative abundance decreased (Fig 6). The relative contribution of *Temora spp*. was higher in this assemblage in 2022 compared to 2008 (Fig 6). In both assemblages the relative contribution of medium sized herring larvae was higher in 2022 than in 2008.

With regard to absolute abundance (S20 and S21 Figs), the GLMM (Table 2) revealed a lower abundance of Appendicularians in the Rhine-Scheldt assemblage in 2022 compared to 2008. The absolute abundance of all other taxa tested remained stable. A striking difference was observed with regard to fish larvae abundance other than herring that was high enough to be considered in the clustering process in 2022 but not in 2008. This indicates a higher larval abundance of fish larvae other than herring in 2022. Furthermore, GLMM detected a higher total larvae abundance in the Channel-Thames region in 2022 than in 2008. A heatmap displaying the inter-annual differences of taxa per grid cell did not reveal a great inter-annual variability of taxa abundance and supported the differences and similarities revealed by the GLMM between the two years (S21 and S22 Figs, S9 File).

**Table 2:** Results of GLMM testing for differences in abundance of dominant taxa between the assemblages independent of year and with regard to inter-annual differences in the same assemblage.

	*Predictors*	(Intercept)	Rhine-Scheldt	Channel-Thames * year2022	Rhine-Scheldt* year2022	dispersion parameter	n
***Temora spp*.**	***Estimates***	0.71	3.37	1.97	1.39	0.000	77
	***std*. *Error***	0.73	1.13	1.00	1.28		
	***p***	0.329	**0.003**	0.050	0.278		
***Paracalanus spp*.**	***Estimates***	2.02	1.71	-0.10	1.19	0.000	77
	***std*. *Error***	0.54	0.91	0.77	1.07		
	***p***	**<0.001**	0.060	0.900	0.264		
**Appendicularia**	***Estimates***	1.77	2.06	-1.24	-2.57	0.213	77
	***std*. *Error***	0.69	1.01	0.98	1.22		
	***p***	**0.011**	**0.041**	0.207	**0.035**		
***Acartia spp*.**	***Estimates***	1.31	1.26	0.38	0.50	0.000	77
	***std*. *Error***	0.48	0.79	0.68	0.92		
	***p***	**0.007**	0.113	0.581	0.588		
**herring larvae (small)**	***Estimates***	4.6716	-0.4083	-0.3036	0.4290	13.8	57
	***std*. *Error***	1.1294	1.1714	0.9323	1.3479		
	***p***	**<0.001**	0.727	0.745	0.750		
**herring larvae (medium)**	***Estimates***	-0.7317	4.3214	2.3202	0.3522	5.84	57
	***std*. *Error***	1.1503	1.3634	1.1935	1.3516		
	***p***	0.52472	**0.00153**	0.05189	0.79440		
**total abundance mesozooplankton**	***Estimates***	4.47	1.70	0.25	0.30	0.127	77
	***std*. *Error***	0.50	0.68	0.69	0.84		
	***p***	**<0.001**	**0.013**	0.719	0.718		
**total abundance fish larvae**	***Estimates***	3.34	1.75	1.95	0.78	0.000	57
	***std*. *Error***	0.58	1.03	0.82	1.22		
	***p***	**<0.001**	0.089	**0.017**	0.525		

The total mean abundance of mesozooplankton and abundance of medium sized herring larvae was higher in the Rhine-Scheldt than in the Channel-Thames assemblage in both years (Table 2).

The statistical evaluation of inter-annual changes by means of GLMM was limited by sampling size for several species. Models integrating cluster and the interaction between cluster and year as predictive variables did not meet model assumptions for *Temora spp*., *Paracalanus spp*. and medium sized herring larvae. The output of these models could be verified, however, by running simplified models testing cluster and year separately allowing us to display the output of the complete model in Table 2, nonetheless.

**Fig 7:**
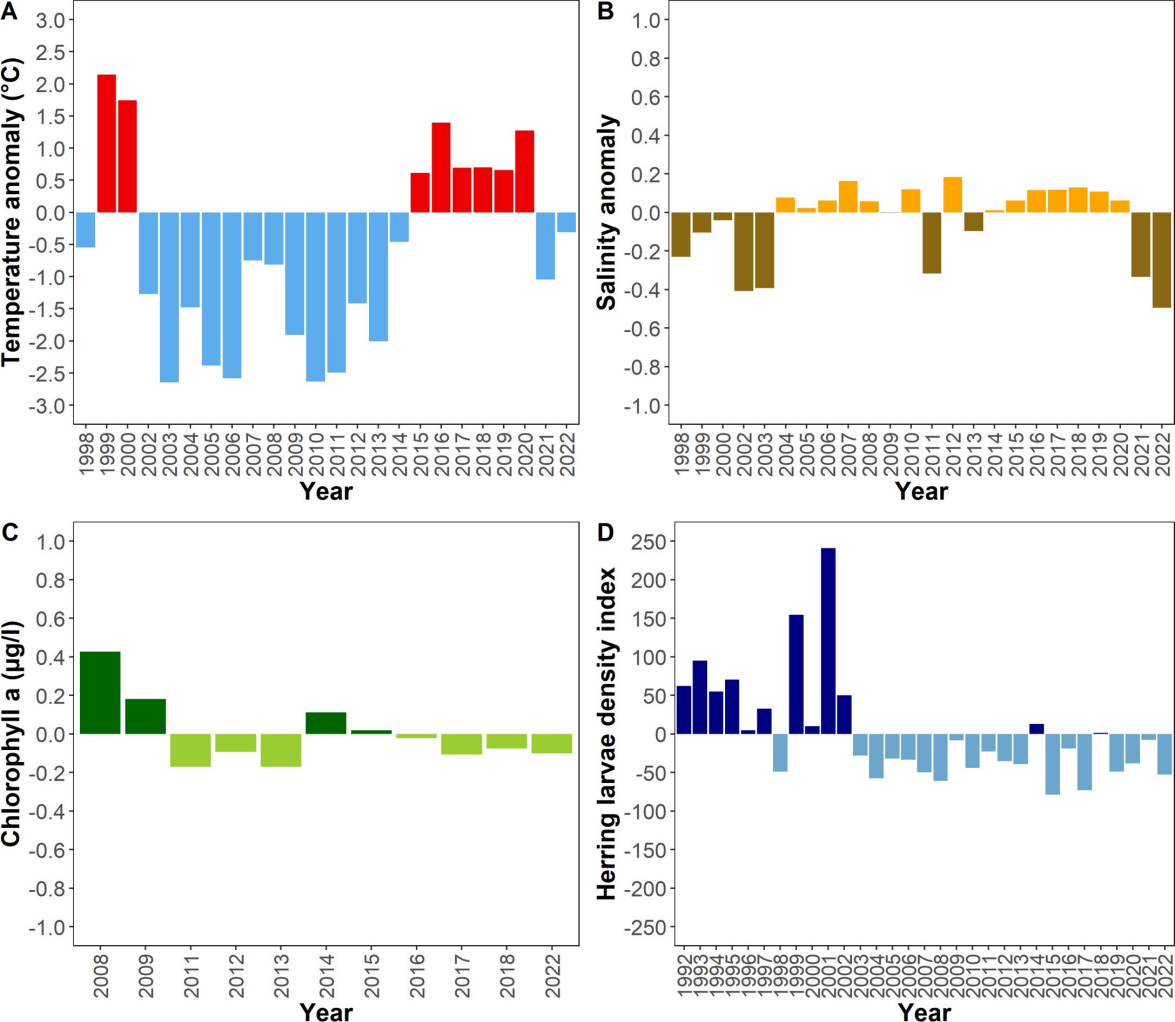
Anomaly analysis. (A) Temperature, (B) Salinity, (C) Chlorophyll a concentration, (D) Herring larvae density index in billions per area. Measurements were taken in the area between 49°N and 55°N during January and February.

An anomaly analysis revealed no exceptional conditions in 2008 or 2022 with regard to temperature, salinity, chlorophyll a and herring larvae density (Fig 7). In comparison with 2022 both years displayed a slight negative temperature anomaly and a comparable negative anomaly in the herring larvae density index which was found to be negative from 2003 to 2022 with only two exceptions.

## Discussion

### Zooplankton assemblages and environmental conditions

Five zooplankton assemblages were found in the SNS-EEC during winter, which differed with regard to taxa abundance and indicator taxa. The spatial distribution of the assemblages appeared related to chlorophyll a concentration, dissolved N/P ratios, phyto- and microplankton composition, advection and fish spawning grounds. In the following, we will first discuss the overall pattern of zooplankton distribution with regard to environmental drivers and secondly the composition of the assemblages separated in their mesozooplankton and ichthyoplankton component with a major focus on indicator taxa.

#### Overall patterns and environmental drivers

The assemblage distribution and composition displayed two overall patterns representing a north south gradient and being related to total zooplankton abundance. The north south gradient was indicated by the dominance of *Oithona spp*. in the northern assemblages (Northern-British coast and Central) and of *Temora spp*. in the southern assemblages (Channel-Thames and Rhine-Scheldt) suggesting the influence of different water masses on the zooplankton assemblages. The northern assemblages can be hypothesized to be influenced by Northern North Sea water and the Central North Sea as further indicated by the presence of *Metridia spp*., Euphausiacea and Gobiidae larvae. *Oithona spp*. were found to display higher abundance in the Northern and Central North Sea in autumn thus elevated abundance in the SNS might indicate advection from the North and/or Center [8]. Due to high abundance of *Metridia spp*. around the Orkney isles the presence of this genera was proposed to indicate the presence of Atlantic water in the North Sea [28, 33]. The finding that the spatial component was the major driver of *Metridia spp*. among the variables tested might be interpreted as further indication of advection. Also Euphausiacea and Gobiidae larvae are known to have a northern distribution during winter and can be hypothesized to be transported by advection into the SNS [33, 88] (see below). Southern assemblages further characterized by herring and Pleuronectidae larvae (Fig 4) were most probably influenced by Channel water and Southern North Sea water which are richer in nitrogen and silicate due to riverine input [89] (Fig 2B and S14 Fig) and which represent drifting routs of herring and plaice larvae [38, 39, 90] (see below). Influence of riverine input on zooplankton species composition was also found by other studies [91].

The German Bight-Norfolk region was not associated to the north south gradient (S10 Fig). It was characterized by *Pseudocalanus spp*. and differed with regard to phyto-microplankton and nutrient composition from the other assemblages.

The second pattern was related to the distribution of total zooplankton abundance. Total zooplankton abundance was elevated in the Rhine-Scheldt and the German Bight-Norfolk assemblage and seemed to be driven by phyto-microplankton biomass (chlorophyll a), phyto-microplankton and nutrient composition. Elevated phyto-microplankton abundance in the Central assemblage did not result in increased zooplankton abundance. Relatively high abundance of phyto-microplankton in the Rhine-Scheldt and the Central assemblages during winter in comparison to the other regions was in accordance with the findings of Dudeck et al. [30], Hay et al. [8], Krause et al. [33], Nielsen et al. [92], Nielsen and Richardson [16] and Groβ et al. [93]. The low zooplankton abundance despite elevated phyto-microplankton abundance in the Central assemblage might be due to lower phytoplankton biomass, differing phyto-microplankton composition and differences in dissolved N/P ratio compared to the Rhine-Scheldt region. Lower chlorophyll a concentration in the Central compared to the Rhine-Scheldt region indicated lower biomass in the former. Whereas diatoms dominated in the Central assemblage, the Rhine-Scheldt assemblage was more diverse as characterized by a mixture of diatoms and the group of other phyto-microplankton. The N/P ratio in the Rhine-Scheldt region was close to the Redfield ratio, which may indicate favorable phytoplankton quality for zooplankton, which was not the case in the Central region during the study period.

The German Bight-Norfolk region displayed elevated zooplankton abundance despite low phyto-microplankton abundance, chlorophyll a and phosphorus concentrations and an elevated dissolved N/P ratio. The low phosphorus concentration found in this region was in accordance with Eberlein [94] and the proportion of nanoflagellates was also reported by Wesche et al. [9] who observed dominance of small flagellates with regard to phytoplankton biomass in winter around Helgoland. The main proportion of total zooplankton abundance was constituted by *Para-* and *Pseudocalanus spp*.. As revealed by the GLMM abundance of *Pseudocalanus spp*. was related to nanoflagellates, the group of other phyto-microplankton, POM, chlorophyll a and phosphate. This indicated that *Pseudocalanus spp*. might be able to profit from the prey composition in this assemblage, despite low prey quantity. Both *Para- and Pseudocalanus spp*. are known to feed on small prey in the size range of flagellates and dinoflagellates [95, 96] and *Pseudocalanus spp*. were reported to feed on flagellates, dinoflagellates and detritus [97 and references therein]. Furthermore, the elevated load of POM could result in a detritus based food web with flagellates and dinoflagellates as intermediate food-level, upgrading prey quality in this region [98]. Another hypothesis takes into account the low temperature in the German Bight-Norfolk region, which by decreasing metabolic costs, could facilitate zooplankton organisms to better deal with low food abundance.

#### Mesozooplankton

The **Northern-British coast region** was the assemblage displaying lowest total zooplankton abundance. This was in accordance with the lower total biomass concentration along the British coast reported by Pitois and Fox [99]. A possible explanation might be the deep depth of this region as using GAM, Dudeck et al. [30] found a negative relationship between zooplankton abundance and depth. As shown by the PCAs on potential environmental drivers deeper depth was related to higher relative salinity and temperature and lower nitrogen concentration indicating a potential multifactor explanation for the low abundance observed in this assemblage. The abundance of the indicator taxon Euphausiacea might indicate the presence of Scottish coastal water in this region. A transport of Euphausiacea by Scottish coastal water to this part of the SNS was also hypothesized by Krause et al. [33] who found a similar distribution of this taxon in winter 1987. Further indication of advection was the finding that all fixed variables tested to explain the distribution of abundance of this taxon were not found significant but the spatial component of the model explained spatial distribution of Euphausiacea. This could indicate that the distribution and presence of this taxon in the Norther-British coast assemblage was mostly due to advection to this precise area but not due to the preference for or avoidance of the variables tested.

An almost similar finding was made for *Metridia spp*. an indicator taxa of the **Central assemblage** and known as indicator taxa for Atlantic water as discussed above. *Oithona spp*., a further indicator taxon [100] of the Central assemblage, was driven by elevated salinity, temperature, deeper depth and low nutrient and chlorophyll a concentration as revealed by the GLMM. These set of drivers reflect the off-shore distribution of this taxon that was absent or very low in abundance in the southern assemblages and the German Bight-Norfolk region, areas characterized by coastal characteristics like shallow depth and increased nutrient concentration, for instance (S14 Fig). As proposed for other taxa displaying higher abundance in the Central North Sea [33], the absence or lower abundance of *Oithona spp*. in the southern assemblages might be the result of water mass circulation. Northwards flowing water masses namely Channel and Southern North Sea water might prevent the protrusion of this genus further south in the SNS. Correlation to elevated salinity, temperature and deep depth might be a further indication of advection of this taxon from the Central and Northern North Sea as for example from the Dogger Bank region. In this area the egg-carrying strategy of Cyclopoid copepods [101] was hypothesized to be advantageous as pelagic eggs might encounter high mortality rates due to elevated predation risk by the benthic suspension feeder community in this shallow and well mixed area [102]. Although chlorophyll a concentration was lower in this assemblage than in the Rhine-Scheldt region, the concentration of chlorophyll a in the Central region was not the lowest in the study area. Thus, the negative correlation of *Oithona spp*. to chlorophyll a concentration appears surprising but could be due to the low explanatory power of A2 axis (16%) that represented chlorophyll a and phosphate. The finding of Chaetognatha and Cnidaria as indicator species for the Central assemblage was in accordance with Krause et al. [33]. They hypothesize that hibernal primary production in the Dogger Bank region in winter 1988 [8, 16, 33, 92] was found to facilitate maintenance of secondary production even sufficient to sustain predators like cnidarians (*Aglantha digitale*, *Pleurobrachia pileus*) and Chaetognaths.

The **Rhine-Scheldt assemblage** appeared to be characterized by taxa associated with elevated phyto-microplankton abundance, biomass and the balanced N/P ratio. Appendicularians, micro- to macrophagous filter feeders [103], were found to reproduce in the Southern Bight [104]. *T*. *longicornis* was reported to reproduce year around [5] with highest production repeatedly observed in hibernating females [9, 105] and to display increased egg production when food concentration is high [9, 10]. Moreover, high nutrient levels and chlorophyll a concentration were revealed as drivers of *Temora* spp. abundance and distribution. The dominance of *Temora spp*. in this assemblage was further in accordance with other investigations [28, 30, 33, 34, 106] pointing to the importance of this taxon in this region during winter. Also, the presence of crustacean larvae and annelid trochophore and metatrochophore larvae might be due to adequate feeding conditions promoting reproduction of these meroplanktonic taxa during winter. Polychaete-larvae were found in similar abundance at a similar location in winter 1987 [33].

The mesozooplankton assemblage of the **Channel-Thames** region was characterized by meroplanktonic taxa and displayed a mesozooplankton composition very similar to the Rhine-Scheldt assemblage but with lower total abundances. Cirripedia larvae, Anomura larvae and ichthyoplankton taxa (see below) were indicator taxa for this region. Cirripedia larvae might find adequate feeding conditions as their abundances were positively related to chlorophyll a concentration that was of average concentration in this region (S14 Fig).

The **German Bight-Norfolk assemblage** differed from northern and southern assemblages and was characterized by *Pseudocalanus spp*. and relatively high proportion of nanoflagellates and dinoflagellates with regard to phyto-microplankton composition. As discussed above, *Pseudocalanus spp*. were positively correlated to nanoflagellate abundances. Furthermore, evidence suggests that *Pseudocalanus spp*. might be able to cope with low prey abundance as it displays a certain resilience to food shortage [107–110] which could be of advantage in this region of low primary production. However, *Pseudocalanus spp*. were also positively correlated to high concentrations of chlorophyll a and phosphate although chlorophyll a and phosphate were found in low concentrations in this region. This apparently contradictory result could be due to the low explanatory power of A2 axis (16%) that represented chlorophyll a and phosphate. A further explanation could be the fact that this genus was also present in the Rhine-Scheldt assemblage although in lower abundance.

A common feature of all assemblages was the dominance of *Paracalanus spp*., in accordance with other studies [33, 111].

The North Sea mesozooplankton community is estimated to consist of 112 species [34, 112], while this study considered 43 taxa of which was none determined until species level. It should be noted that we used different levels of taxonomic resolution to keep a maximum of information, an approach applied by several other studies [33, 45]. Nonetheless, we expect the use of a finer taxonomic resolution to result in similar assemblages but to potentially strengthen the distinction of the assemblages we found [33]. Predicting future changes in the study area during winter, e.g. in response to climate change, is an interesting aspect which future studies should focus on. To that purpose, a taxonomic resolution at species level would be necessary as each species has characteristics of its own, e.g., biology, sensitivity to environmental and community changes [113] and may play a specific role in the food web [114].

#### Ichthyoplankton

Winter zooplankton assemblages also differed with regard to ichthyoplankton. Based on the present study the southern assemblages, Channel-Thames and Rhine-Scheldt, can be judged particularly important for the offspring of several fish species as they harbor spawning and nursery areas and drifting routes. This was specifically true for plaice and herring larvae for which the study period represented peak spawning time [35, 36, 115, 116]. By contrast common dab (*Limanda limanda*), flounder (*Platichthys flesus*), sole (*Solea solea*), whiting (*Merlangius merlangus*) and cod (*Gadus morhua*) were at the beginning of their spawning period indicating the potential for higher inter-annual variability in the contribution of these species to the assemblages. The smallest size class of herring larvae outnumbered the larval assemblage in the **Channel-Thames region**, a region harboring several spawning grounds of Downs herring [e.g. 39]. The drift of herring larvae spawned in the EEC can explain the finding of the size classes 13–20 mm and 21–42 mm being an indicator taxa of the Rhine-Scheldt assemblage [39, 62]. Although larval drift and retention is inter-annually highly variable [39, 62], the arrival of larvae bigger than 12 mm in the Southern Bight and smaller larvae being rather situated in the EEC at the moment of the first quarter IBTS survey appears to be a recurring pattern [29, 62]. Thus, we hypothesize that small herring larvae are a reoccurring characteristic component of the Channel-Thames assemblage with larger herring larvae being associated to the Rhine-Scheldt region. Environmental drivers differed between the two size classes with the small larvae being predominantly correlated to the abundance of nanoflagellates and the group of other phytoplankton and secondly to nitrogen, silicate and POM concentration whereas medium sized larvae were marginally related to chlorophyll a concentration indirectly corresponding to the elevated zooplankton abundance in the Rhine-Scheldt assemblage. The high explicative power of the spatial component for the abundance of medium sized larvae might be interpreted as indicator of larval drift being a further driver of this taxon’s distribution. Nevertheless, an inter-annual variability of the contribution of the different herring larvae size classes to the Channel-Thames and Rhine-Scheldt assemblages has to be expected. A region corresponding to the South of the Rhine-Scheldt assemblage (French and Belgium coast) was considered advantageous with zooplankton biomass in December comparable to the Buchan/Banks areas in September and low proportions of starved larvae [29]. Prey of medium sized herring larvae like *Paracalanus spp*. and *Temora spp*. [62] where of high abundance in the Rhine-Scheldt assemblage. Interestingly, Dickey-Collas et al. [39] found a positive relation of retention of herring larvae close to their spawning grounds, meaning larval drift not surpassing the Rhine-Scheldt delta, with a higher recruitment index of Downs herring. This indicates that although elevated plankton abundance was found in the entire Rhine-Scheldt assemblage in the present study this might not been the case in earlier years investigated by the mentioned authors (1988–2003). Or, that due to spatial variation of plankton or other factors influencing larval feeding within the assemblage the area until the Rhine-Scheldt delta provides better feeding conditions than the rest of this assemblage. Small herring larvae characterizing the Channel Thames region were likely distributed in the vicinity of spawning areas. The relation to nanoflagellates could indicate a higher abundance of microzooplanktonic prey [117] but a direct relation to prey abundance could not be revealed in the present study due to the lack of detailed microzooplankton data.

Pleuronectidae larvae, an indicator taxon of the **Rhine-Scheldt assemblage**, most probably consisted of plaice larvae (*P*. *platessa*) with regard to the main spawning period [37, 116]. These larvae most probably originated from the spawning grounds in the EEC and Southern Bight, where spawning starts in December and January, respectively [38, 90, 118], and which are mainly connected to the nurseries in the Scheldt estuary [118] and along the Dutch Wadden Sea [38]. As plaice larvae were found to nearly exclusively prey on Appendicularians in the study area [118, 119], it can be hypothesized that the Rhine-Scheldt assemblage is suitable for drifting plaice larvae with regard to prey provision as abundance of Appendicularians accounted for 15% of the mesozooplankton assemblage in this region. As larvae of both the EEC and the Southern-North Sea spawning grounds provide larvae to the Rhine-Scheldt assemblage we assume that plaice larvae will constitute a reoccurring member of this assemblage despite the inter-annual variability in larval drift predicted by Bolle et al. [38]. Eggs of plaice characterized the Central assemblage which is in accordance with the location of the spawning ground south of the Dogger Bank [118, 120, 121] for which spawning was reported to peak during February and March [90]. Pleuronectidae eggs other than plaice were revealed as indicator species of the Rhine-Scheldt assemblage. They were most probably belonging to common dab (*Limanda limanda*) and flounder (*Platichthys flesus*) as egg distribution corresponded to spawning periods and spawning grounds [35, 36, 122–124].

Further indicator species of the Rhine-Scheldt region were the eggs of *Solea solea* and Lotidae which was in accordance with the spawning areas of these taxa. Spawning grounds of Lotidae species like *Ciliata mustela* are located along the eastern coast of the SNS with centers off the Belgium and Dutch coast up to the Frisian islands with a spawning period reported to start in January [36]. Sole spawns in the EEC, the Thames estuary and along the Belgian coast [125, 126].

Although spawning grounds of sardine exist in the German and Southern Bight, sardine larvae were observed in summer (June–August) in these areas [127, 128]. Spawning in the EEC by contrast continued until October (July–October) [129]. We can thus assume that the larvae characterizing the Channel-Thames assemblage, displaying a size between 16–40 mm, were drifted to the Channel-Thames region from the EEC.

Further north, the eastern part of the **German Bight-Norfolk region** appeared important for Syngnathidae larvae most probably *S*. *rostellatus* consistent with previous works [37, 130].

Gobiidae larvae were found to be an indicator species of the **Northern-British coast assemblage**. The only species reported to reproduce in the North Sea during winter was *Pomatochistus minutus* with a spawning period from February to June off Scotland [88]. One could thus hypothesize that larvae hatched off Scotland drifted into the Northern-British coast assemblages with the Scottish Coastal water mass.

Drivers of taxa distribution not considered in this study could further have influenced the distribution of the assemblages found. This was indicated by the finding that for six taxa the abundance in the assemblages could not be entirely explained with the explicative variables used as indicated by retention of the spatial component in the most appropriate model (Table 1). A possible driver not considered in this study is the effect of top-down control that might be exerted by zooplanktonic taxa like fish larvae, carnivorous copepods and by planktivorous fish. Although the North Sea as a whole is considered as bottom-up controlled [48], top- down control exerted by planktivorous fish was proposed to play a role in subareas of the North Sea [48, 131] and especially during autumn and winter when secondary production is low [48, 49, 132]. Whiting displayed an increased consumption of *T*. *longicornis* in the Channel-Thames and Rhine-Scheldt region in winter [132] and calculated consumption of herring and sprat juveniles exceeded production of investigated copepod species in the Scheldt estuary during this season [49].

#### Inter-annual changes in zooplankton assemblages

The overall mesozooplankton taxa composition and total abundance as well as herring larvae abundance were relatively stable comparing 2008 and 2022 despite the long time span elapsed between these two years. This finding gives further evidence for an inter-annual persistence or reoccurrence of elevated secondary production in the Rhine-Scheldt assemblage during winter as was reported by several other studies [30, 32, 33]. The inter-annual comparison further supported the finding of medium sized herring larvae as indicator of the Rhine-Scheldt assemblage as abundance of this taxon was higher in this assemblage in both years. Overall, the only notable differences between the two periods were found in the Rhine-Scheldt assemblage with a lower abundance of Appendicularians in 2022 and in the Channel Thames region with higher total fish larvae abundance in 2022. The decline of Appendicularians might be related by the increased abundance of plaice larvae (Fig 6 and S20 Fig) known to feed mainly on *Oikopleura doika* [118, 119]. With regard to fish larvae, abundance of species other than herring (plaice, sardine, Gobiidae, Ammodytidae) was higher in 2022 compared to 2008. An increase in sardine larvae in the southern and south-eastern North Sea but also further north is a phenomenon observed for several years [41].

Other studies have found inter-annual differences in mesozooplankton abundance in the study area between 2008–2022. An increase in mesozooplankton abundance was observed by Dudeck et al. [30] between 2010–2013 in both the EEC and the Southern Bight. Semmouri et al. [114] described a decrease in abundance of *T*. *longicornis*, *A*. *clausi*, *Centropages sp*. and *Calanus helgolandicus* from 2015 to 2022 in Belgian waters. With regard to mesozooplankton abundance before 2008 the present study was in accordance with the reported decreasing trend of zoo- and holoplankton [25, 99, 133]. Compared to winter 1987 [33] the abundance of *Acartia spp*. and *Pseudocalanus spp*. were distinctively lower, for instance. Thus, the stability observed when comparing 2008 and 2022 does not fully reflect the inter-annual variability occurring between these two years.

However, there are reasons to believe that the assemblages found in the big polygon in 2008 displayed a representative pattern of winter zooplankton assemblages in the study area. First, the assemblages were related to the distribution of different water masses and other work (see above) [54, 56]. Second, higher abundance of phytoplankton in the Dogger Bank region and of plankton in the Rhine-Scheldt assemblage were found in several years by several authors [8, 16, 30, 33, 92, 93]. Third, the overall distribution of taxa was similar to 2022. Fourth, 2008 did not display strong anomalies with regard to salinity, temperature, chlorophyll a and herring larval density.

## Conclusion

The present study suggests the existence of five zooplankton assemblages in the Southern North Sea and Eastern English Channel during winter that vary with regard to productivity, taxa abundance and composition. Chlorophyll a concentration, dissolved N/P ratios, phyto- and microplankton composition, water masses and fish spawning grounds were revealed as major driver of assemblage distribution. The Rhine-Scheldt and German Bight-Norfolk assemblages harbored the biggest zooplankton overwintering stocks that might influence the grazing pressure on phytoplankton spring production. Furthermore, elevated phyto-microplankton abundance in the Central region indicated this assemblage being a center of early spring plankton production. The distribution of ichthyoplankton taxa within the assemblages corresponded to spawning grounds and drifting routes of fish species with the Channel-Thames and Rhine-Scheldt assemblages being of high importance for herring and plaice larvae.

Future studies would profit from the integration of taxonomical data of microplankton (<200μm) facilitating the distinction of developmental stages of copepods, for instance, that would enhance the understanding of fish larvae distribution with regard to prey availability [29, 62] and give further insight in overwintering strategies of zooplanktonic organisms. Anomaly analysis of environmental and biological parameters, comparison with zooplankton data sampled in 2022 and earlier work suggest that the 2008 assemblages are broadly representative of the mesozooplankton and ichthyoplankton spatial distribution. Nonetheless, the evaluation of spatial data from other years would clearly enhance and verify our understanding of hibernal zooplankton assemblages and their potential influence on seasonal plankton succession in the context of climate change.

## Supporting information

S1 Fig Sampling stations IBTS 2008. (A) Water samples for phyto- and microplankton community analysis, (B) Water samples for POM, nitrate, nitrite, ammonium, phosphate, silicate and chlorophyll a, (C) Salinity, temperature and depth.
(TIF)

S2 Fig Empirical and theoretical variograms of species used for the determination of optimal grid cell size. (A) Small-sized herring larvae (6–12 mm), variogram model fitted to raw data; B; medium-sized herring larvae (13–20 mm), variogram fitted to raw data; (C) Appendicularia, variogram fitted to residuals of quadratic regression; (D) Acartia, variogram fitted to residuals of linear regression; (E) Calanoida, variogram fitted to residuals of linear regression, (F) Pseudocalanus, variogram fitted to residuals of linear regression; (G) Centropages, variogram fitted to residuals of quadratic regression.
(TIF)

S3 Fig Taxon-specific optimal grid cell size (Lopt). Functions display maximal information content per cell (f(v)) depending on grid cell size (length and width in km) calculated for different taxa. PN = proportion of nugget variance removed, PS = proportion of sill variance retained. Lopt indicates taxon-specific optimal grid cell size. The median of the species specific Lopt (91.58 km, 0.83°) produced a single cell of central position not containing sampling stations and was thus accepted without further adjustment.
(TIF)

S4 Fig Number of sampling stations per cell. (A) Grid with the optimal grid cell size of 0.83 x 0.83 degrees, (B) Number of sampling stations for mesozooplankton per grid cell, (C) Number of sampling stations for fish larvae per grid cell, (D) Number of sampling stations for fish eggs per grid cell, (E) Number of sampling stations salinity, temperature and depth, (F) Number of sampling stations of water samples for phytoplankton community analysis, (G) water samples for concentration of material in suspension and chlorophyll a.
(TIF)

S5 Fig Schematic representation of the methodological approach. (TIF)

S6 Fig Statistical methods to determine optimal number of clusters (2008 big spatial extent). (A) Silhouette width indicating an optimum number of three clusters, (B) Kelly-Gardner-Sutcliffe penalty function proposing an optimal number of four clusters, (C) Mantel correlation indicating an optimal number of four clusters.
(TIF)

S7 Fig Highest membership values per cell for clustering using different k. Fuzzy clustering evaluates the strength of affiliation of a cell to each cluster, which is expressed by a membership value. High membership values indicate coherent regions, low membership values indicate regions of low coherence. (A) three clusters (maximized silhouette width), (B) four clusters (minimized Kelly-Gardner-Sutcliffe penalty and maximized Mantel correlation), (C) five clusters (quasi minimized Kelly-Gardner-Sutcliffe penalty, quasi maximized Mantel correlation and quasi maximized silhouette width).
(TIF)

S8 Fig Number of clusters/cut-off levels tested. (A) three clusters (maximized silhouette width), (B) four clusters (minimized Kelly-Gardner-Sutcliffe penalty and maximized Mantel correlation), (C) five clusters (quasi minimized Kelly-Gardner-Sutcliffe penalty, quasi maximized Mantel correlation and quasi maximized silhouette width).
(TIF)

S9 Fig Percentage of explained variance of dimensions of PCA on taxonomical data big spatial extent. (TIF)

S10 Fig PCA applied to zooplankton taxa. (A) Taxa displayed in a two-dimensional space of the PCA. (B) Assemblages/cluster displayed in the same two-dimensional space as in A. Numbers indicate grid cell ID.
(TIF)

S11 Fig Mean abundance of the most important taxa per cluster. From left to right: mesozooplankton, fish larvae other than herring, three different size classes of herring larvae, and fish eggs. Each row represents one assemblage. Only the most structuring taxa were displayed (excluded taxa: Centropages spp., Euterpina spp., Lotidae (eggs) and Solea solea (eggs)). The same Fig but using individually scaled y-axis was provided in the supplementary material (Fig S 10).
(TIF)

S12 Fig Mean abundance of taxa per cluster with individually scaled y-axis. Mean abundance is therefore not comparable between clusters but gives further inside in community per cluster.
(TIF)

S13 Fig Drivers per assemblage. Boxplots displaying the variability of a potential driver per assemblage. Beginning in the upper left corner continuing to the right: temperature, salinity, depth, concentration of particulate organic matter, concentration of chlorophyll a, total phyto- and microplankton abundance and Nitrogen/Phosphorus ratio.
(TIF)

S14 Fig Distribution of nutrients and chlorophyll a. Maps display mean per grid cell of nutrients sampled during the IBTS 2008. (TIF)

S15 Fig PCA on potential abiotic and biotic drivers. (A) Abiotic parameters displayed in a two-dimensional space of a PCA. (B) Assemblages/cluster displayed in the same two-dimensional space as in A. Numbers indicate grid cell ID. (C) Phyto- and microplankton groups displayed in a two-dimensional space of a PCA. (D) Assemblages/cluster displayed in the same two-dimensional space as in C. Numbers indicate grid cell ID.
(TIF)

S16 Fig Boxplots of PCA dimensions per cluster. (A) First dimension of PCA applied to abiotic parameters. (B) Second dimension of PCA applied to abiotic parameters. (C) First dimension of PCA applied to biotic parameters.
(TIF)

S17 Fig Relative phyto- microplankton composition per zooplankton assemblage. (TIF)

S18 Fig Statistical methods to determine optimal number of clusters (Comparison 2008 vs 2022, small spatial extent). (A) Silhouette width indicating an optimum number of three clusters, (B) Kelly-Gardner-Sutcliffe penalty function proposing an optimal number of four clusters, (C) Mantel correlation indicating an optimal number of four clusters.
(TIF)

S19 Fig Clusters received using different k. (A) Clusters derived using a k = 2; (B) Clusters derived using a k = 3.
(TIF)

S20 Fig Taxa composition of assemblages in 2008 (left) and 2022 (right). Mean abundance of taxa was calculated with regard to the spatial distribution of clusters in 2008 for both years.
(TIF)

S21 Fig Difference in abundance between 2008 and 2022 per grid cell. Abundance in 2008 was subtracted form abundance in 2022. A: Heatmap displaying increase or decrease per taxon and cell. B: Difference of total abundance of mesozoo- and ichthyoplankton per grid cell.
(TIF)

S22 Fig Inter-annual differences of environmental conditions. Measurements taken in 2008 were subtracted from measurements taken in 2022 so that positive values indicate an increase in 2022 compared to 2008. Numbers in grid cells indicate grid cell ID. A: Temperature; B: Salinity; C: Chlorophyll a; D: Particulate organic matter.
(TIF)

S1 Table Taxa determined in 2008 sorted by taxonomic level. (DOCX)

S2 Table Taxa determined in 2022 sorted by taxonomic level. (DOCX)

S1 File Taxa determination mesozooplankton. (PDF)

S2 File Calculation of mesozooplankton abundance. (PDF)

S3 File Calculation of fish larvae and fish eggs, phyto-microplankton. (PDF)

S4 File Determination optimal grid cell size. (PDF)

S5 File Choice of taxa for clustering. (PDF)

S6 File Pilot study clustering method. (PDF)

S7 File Definition of optimal number of clusters. (PDF)

S8 File Detailed characterization of assemblages. (PDF)

S9 File Maps and heatmaps of inter-annual differences of taxa abundance and environmental parameters per grid cell. (PDF)
