# Supplementary figures and images for: Winter distribution of zooplankton and ichthyoplankton assemblages in the North Sea and the English Channel
Source: PLoS One. 2024 Oct 7;19(10):e0308803. doi: 10.1371/journal.pone.0308803 (PMC11458026; doi:10.1371/journal.pone.0308803)

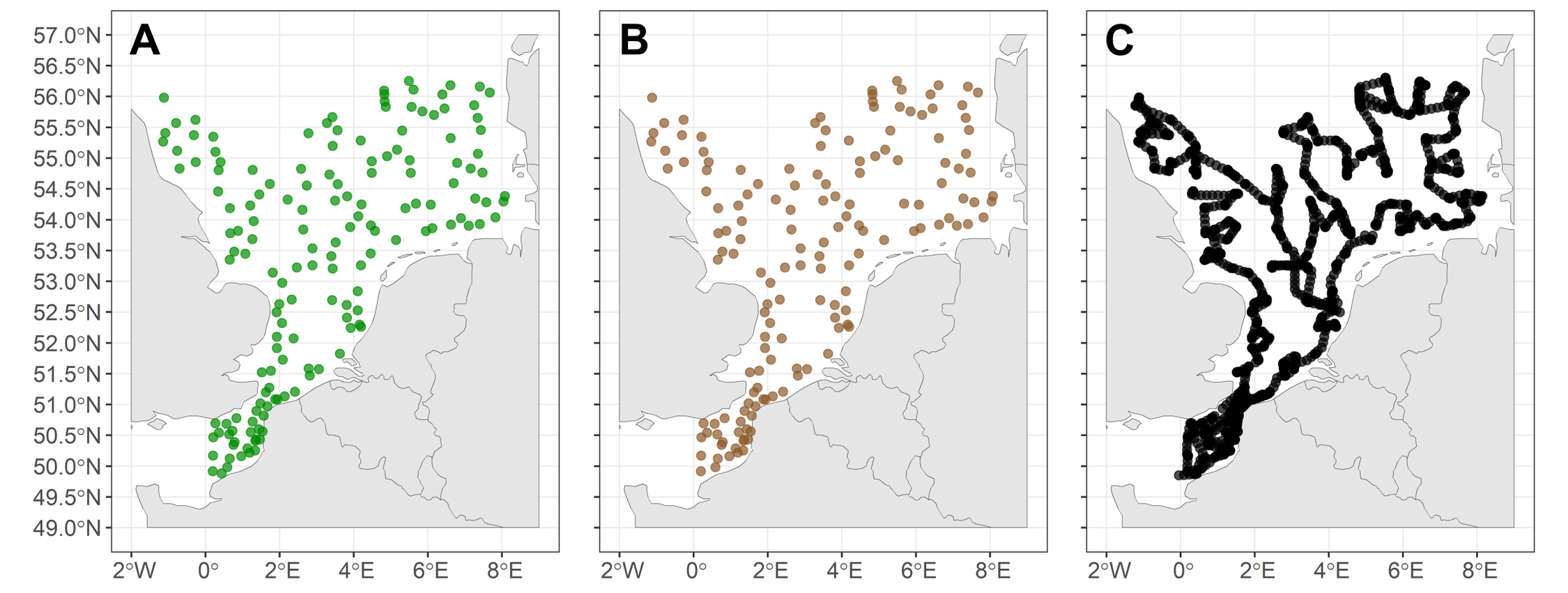

Supplement: S1 Fig — (A) Water samples for phyto- and microplankton community analysis, (B) Water samples for POM, nitrate, nitrite, ammonium, phosphate, silicate and chlorophyll a, (C) Salinity, temperature and depth. (TIF) [file pone.0308803.s001.tif]

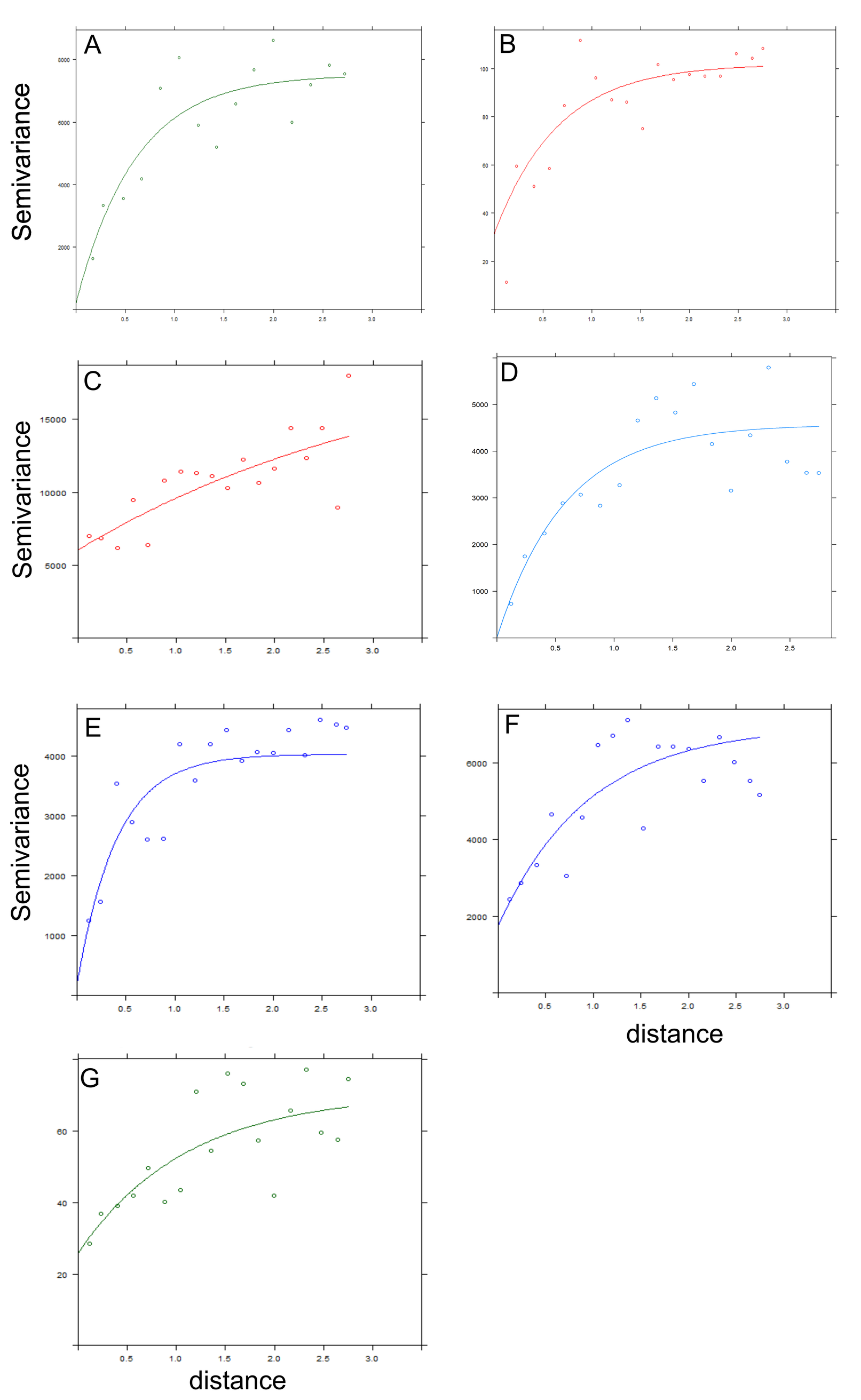

Supplement: S2 Fig — (A) Small-sized herring larvae (6–12 mm), variogram model fitted to raw data; B; medium-sized herring larvae (13–20 mm), variogram fitted to raw data; (C) Appendicularia, variogram fitted to residuals of quadratic regression; (D) Acartia, variogram fitted to residuals of linear regression; (E) Calanoida, variogram fitted to residuals of linear regression, (F) Pseudocalanus, variogram fitted to residuals of linear regression; (G) Centropages, variogram fitted to residuals of quadratic regression. (TIF) [file pone.0308803.s002.tif]

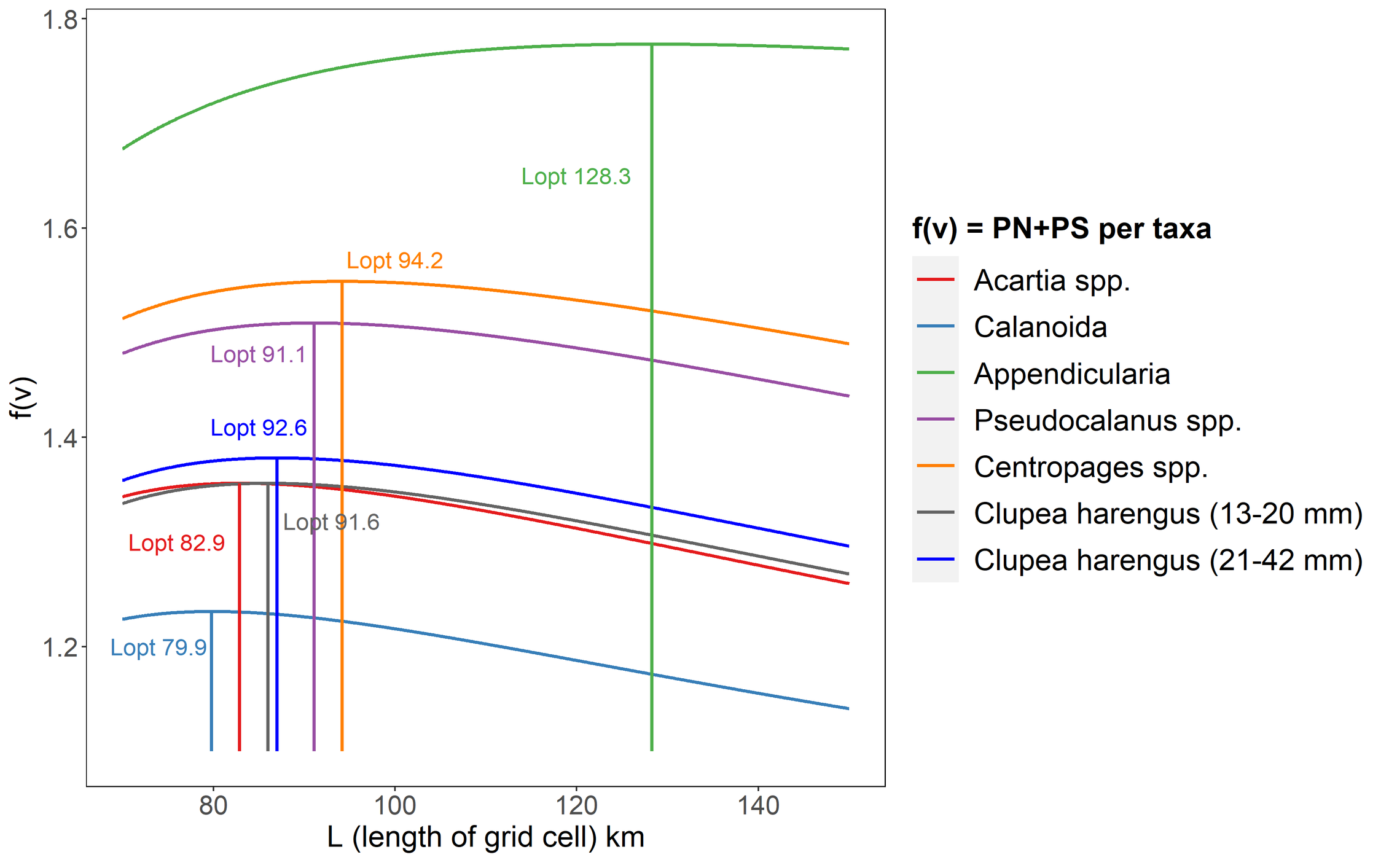

Supplement: S3 Fig — Functions display maximal information content per cell (f(v)) depending on grid cell size (length and width in km) calculated for different taxa. PN = proportion of nugget variance removed, PS = proportion of sill variance retained. Lopt indicates taxon-specific optimal grid cell size. The median of the species specific Lopt (91.58 km, 0.83°) produced a single cell of central position not containing sampling stations and was thus accepted without further adjustment. (TIF) [file pone.0308803.s003.tif]

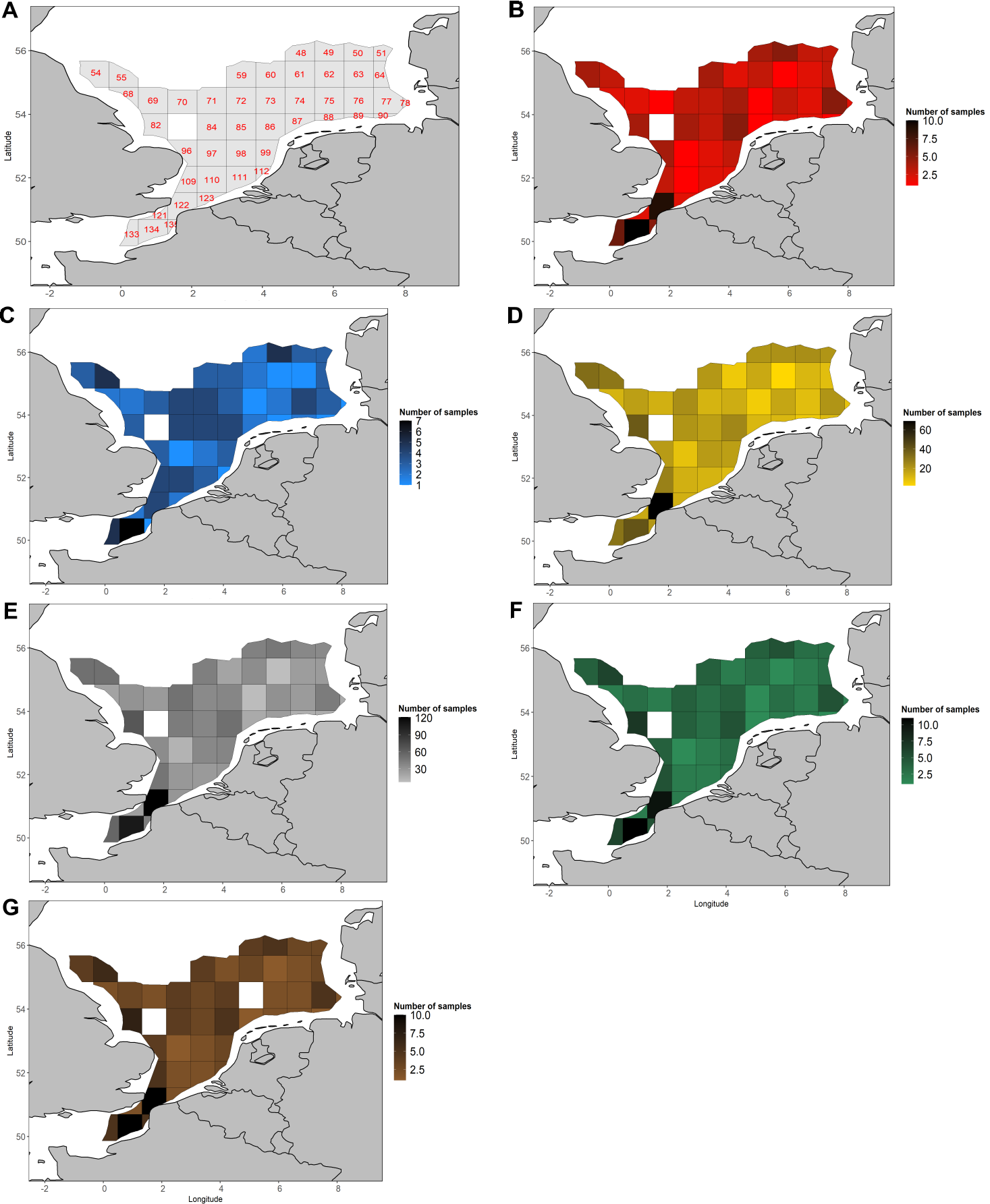

Supplement: S4 Fig — (A) Grid with the optimal grid cell size of 0.83 x 0.83 degrees, (B) Number of sampling stations for mesozooplankton per grid cell, (C) Number of sampling stations for fish larvae per grid cell, (D) Number of sampling stations for fish eggs per grid cell, (E) Number of sampling stations salinity, temperature and depth, (F) Number of sampling stations of water samples for phytoplankton community analysis, (G) water samples for concentration of material in suspension and chlorophyll a. (TIF) [file pone.0308803.s004.tif]

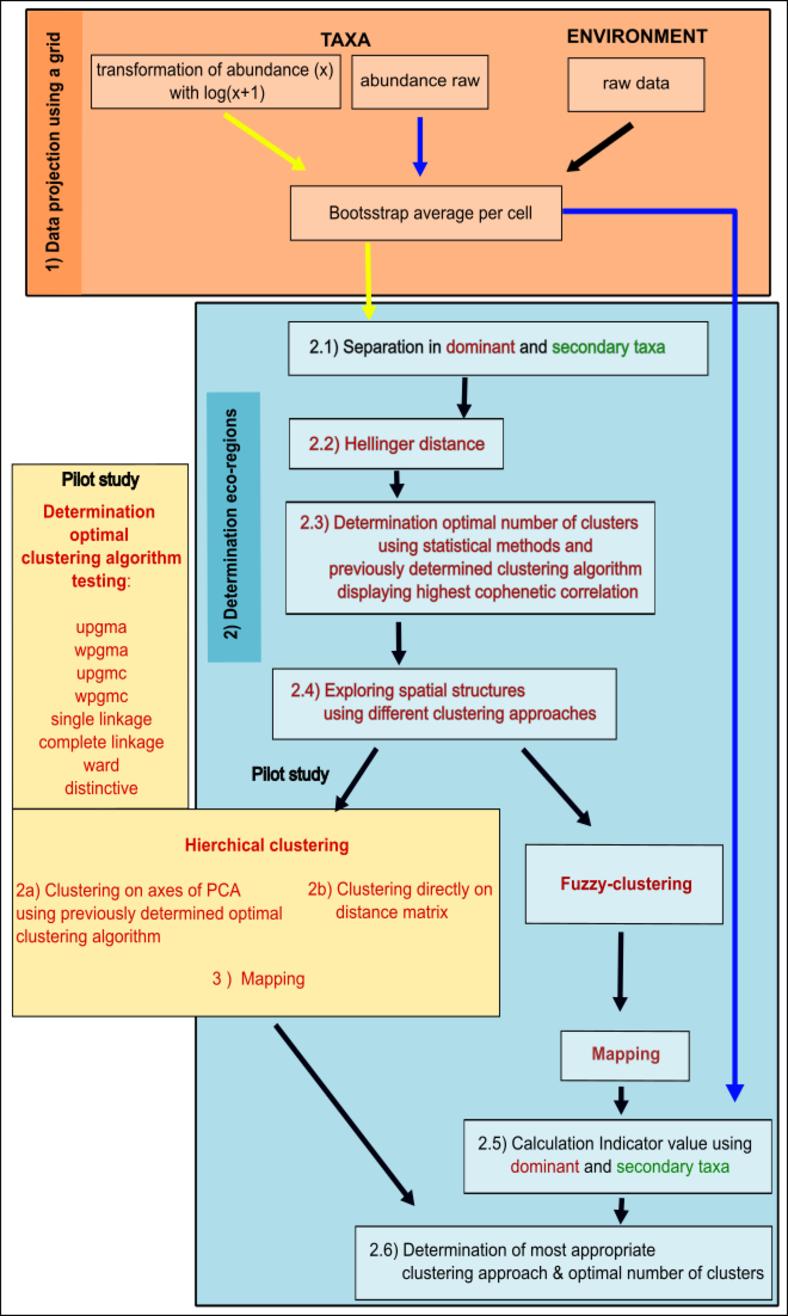

Supplement: S5 Fig — (TIF) [file pone.0308803.s005.tif]

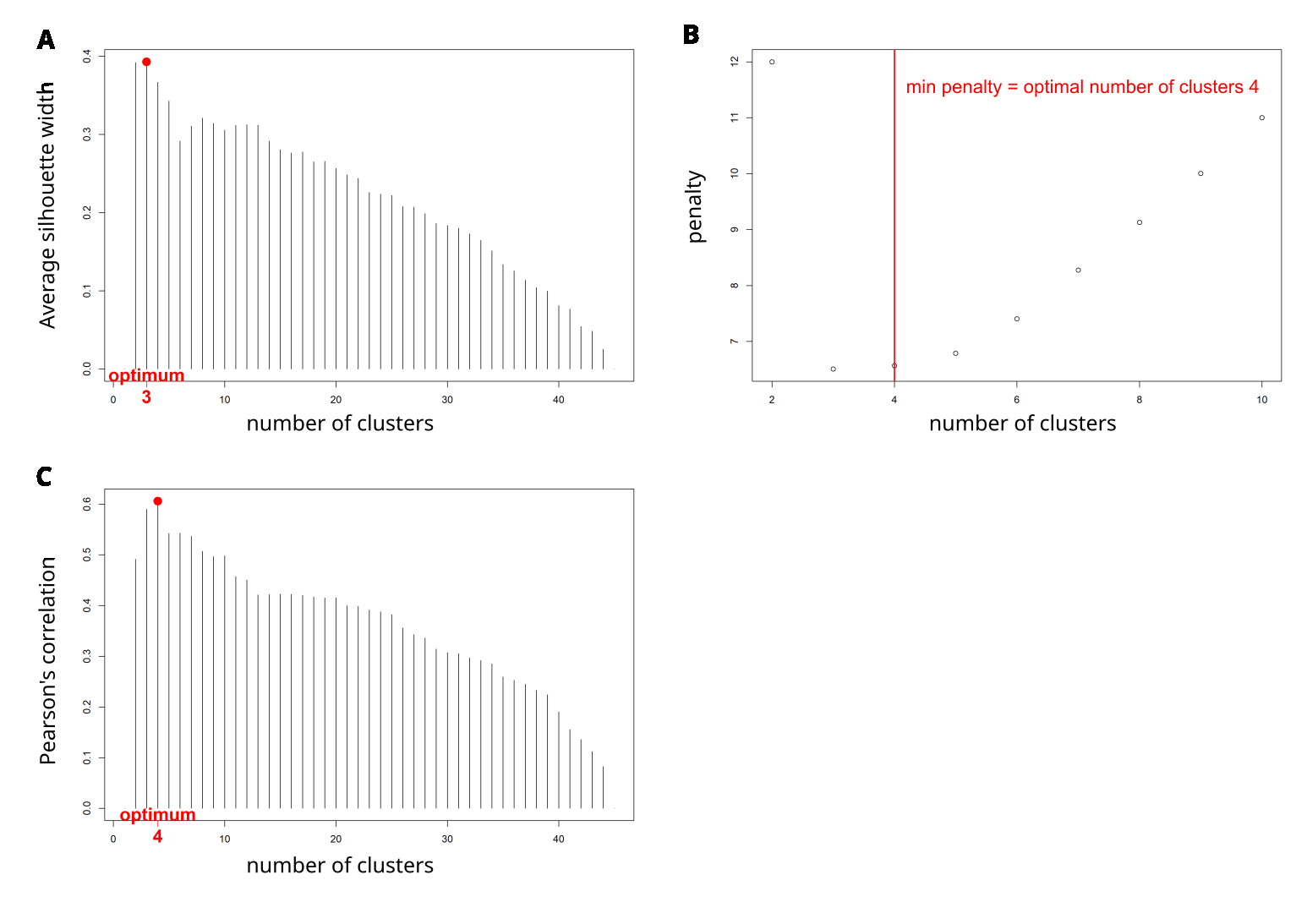

Supplement: S6 Fig — (A) Silhouette width indicating an optimum number of three clusters, (B) Kelly-Gardner-Sutcliffe penalty function proposing an optimal number of four clusters, (C) Mantel correlation indicating an optimal number of four clusters. (TIF) [file pone.0308803.s006.tif]

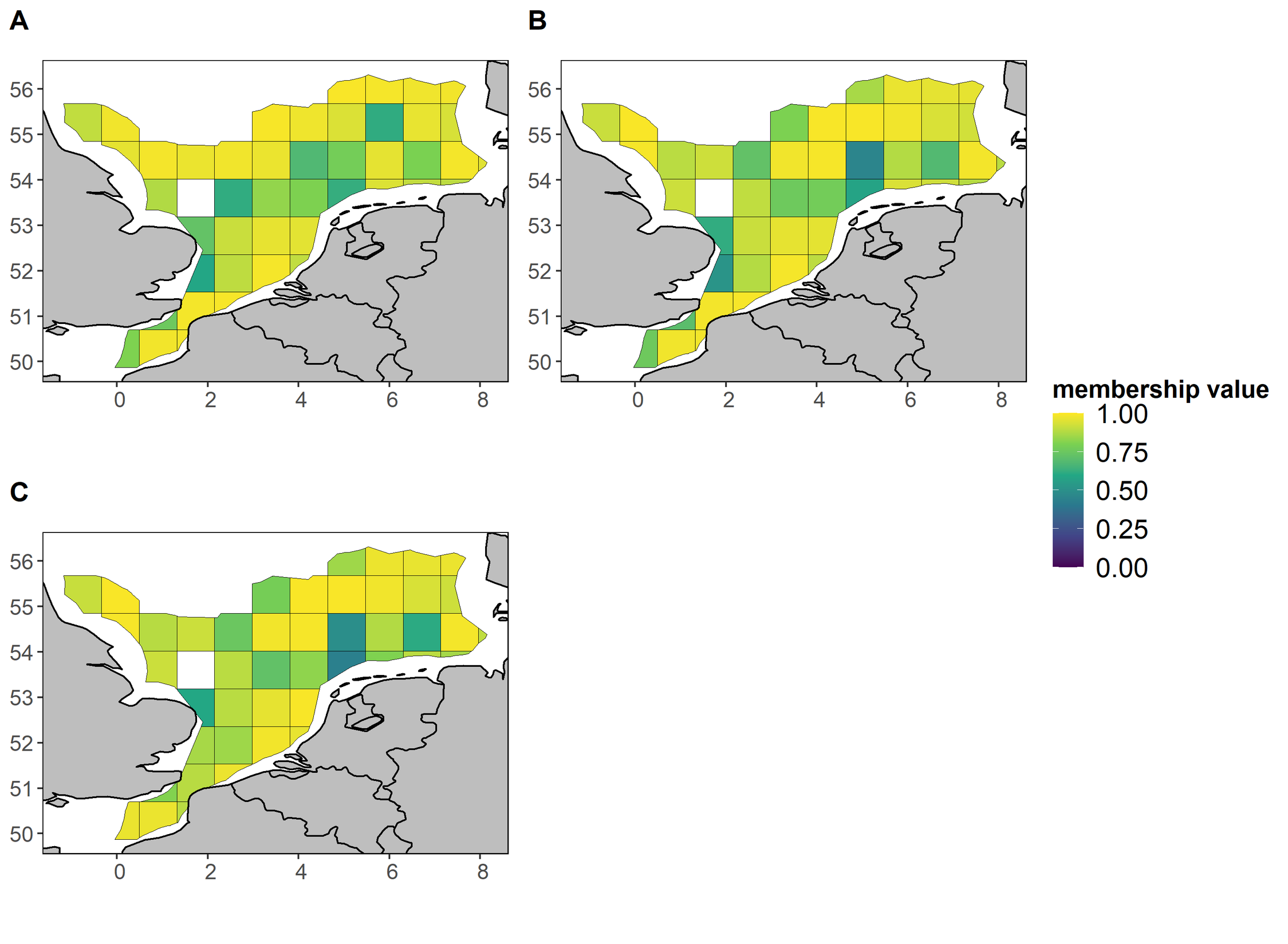

Supplement: S7 Fig — Fuzzy clustering evaluates the strength of affiliation of a cell to each cluster, which is expressed by a membership value. High membership values indicate coherent regions, low membership values indicate regions of low coherence. (A) three clusters (maximized silhouette width), (B) four clusters (minimized Kelly-Gardner-Sutcliffe penalty and maximized Mantel correlation), (C) five clusters (quasi minimized Kelly-Gardner-Sutcliffe penalty, quasi maximized Mantel correlation and quasi maximized silhouette width). (TIF) [file pone.0308803.s007.tif]

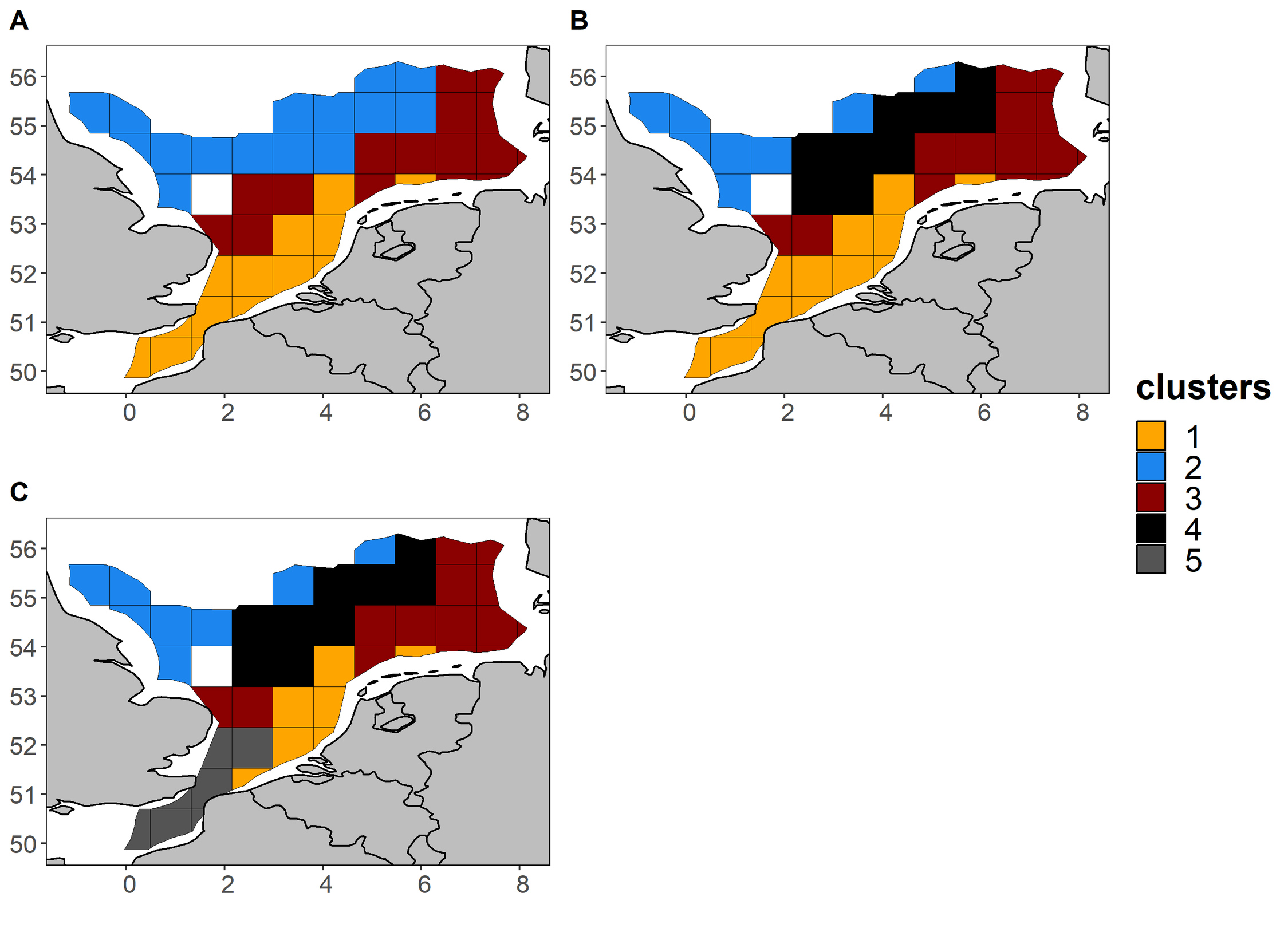

Supplement: S8 Fig — (A) three clusters (maximized silhouette width), (B) four clusters (minimized Kelly-Gardner-Sutcliffe penalty and maximized Mantel correlation), (C) five clusters (quasi minimized Kelly-Gardner-Sutcliffe penalty, quasi maximized Mantel correlation and quasi maximized silhouette width). (TIF) [file pone.0308803.s008.tif]

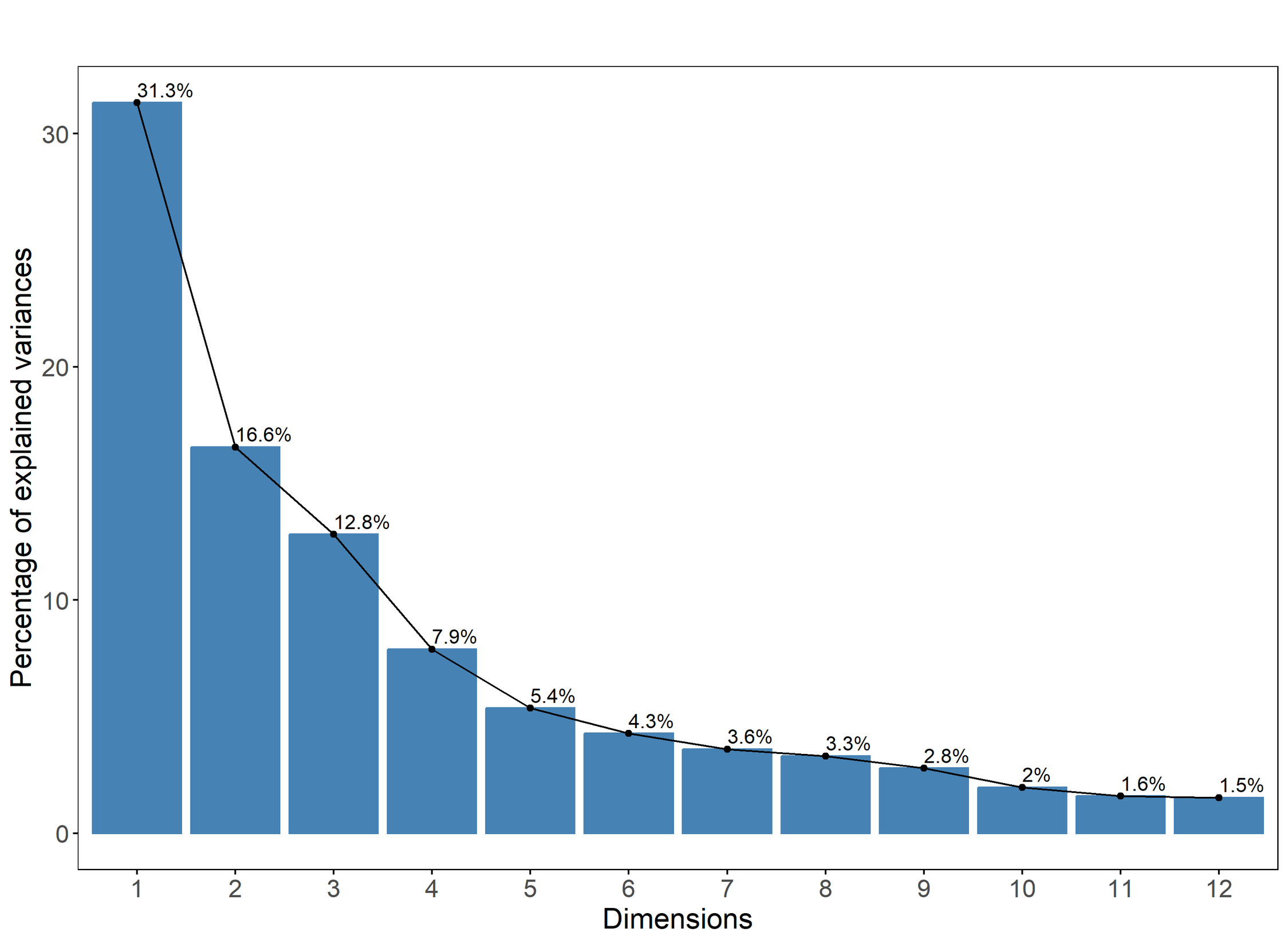

Supplement: S9 Fig — (TIF) [file pone.0308803.s009.tif]

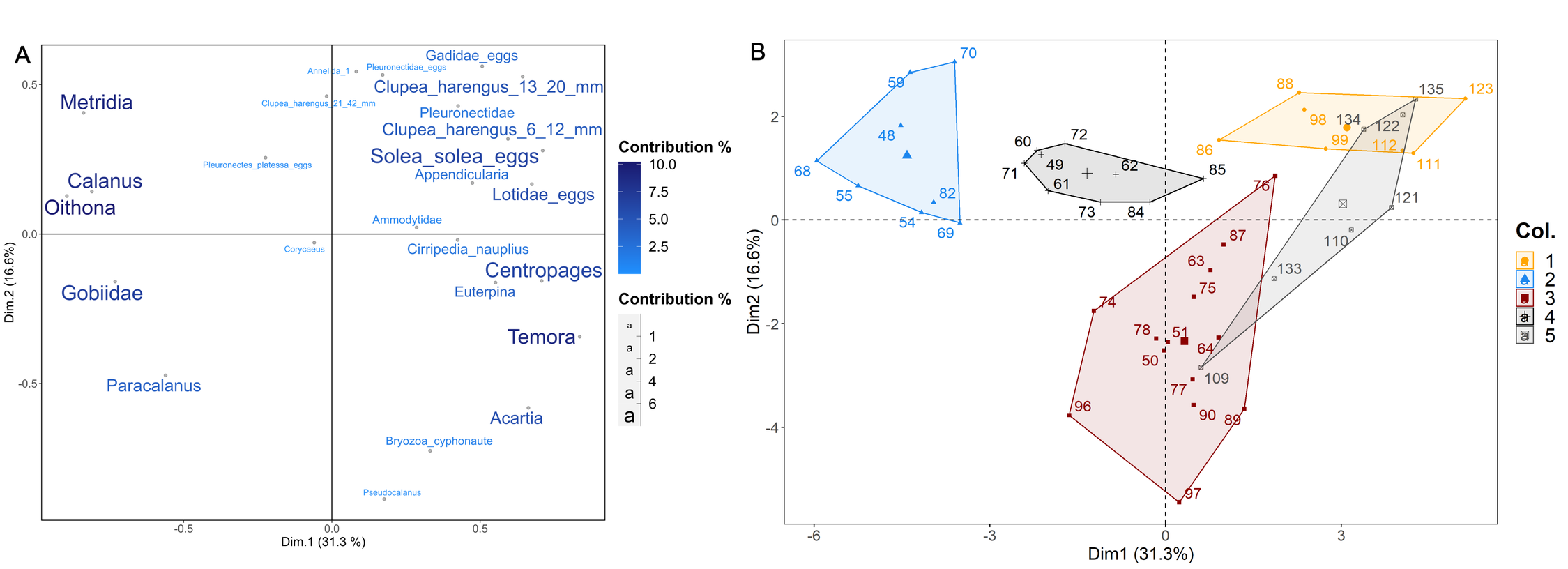

Supplement: S10 Fig — (A) Taxa displayed in a two-dimensional space of the PCA. (B) Assemblages/cluster displayed in the same two-dimensional space as in A. Numbers indicate grid cell ID. (TIF) [file pone.0308803.s010.tif]

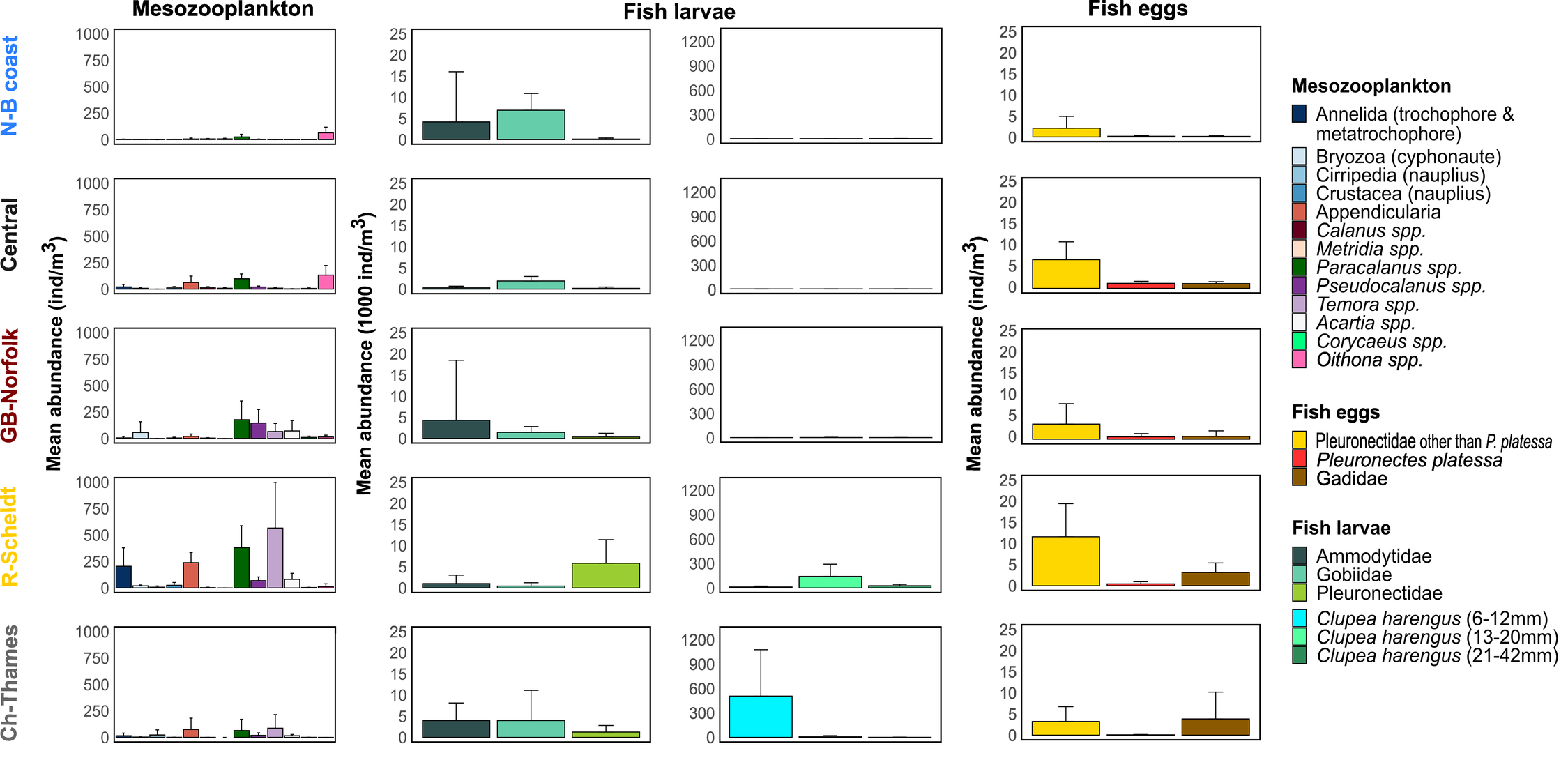

Supplement: S11 Fig — From left to right: mesozooplankton, fish larvae other than herring, three different size classes of herring larvae, and fish eggs. Each row represents one assemblage. Only the most structuring taxa were displayed (excluded taxa: Centropages spp., Euterpina spp., Lotidae (eggs) and Solea solea (eggs)). The same Fig but using individually scaled y-axis was provided in the supplementary material (Fig S 10). (TIF) [file pone.0308803.s011.tif]

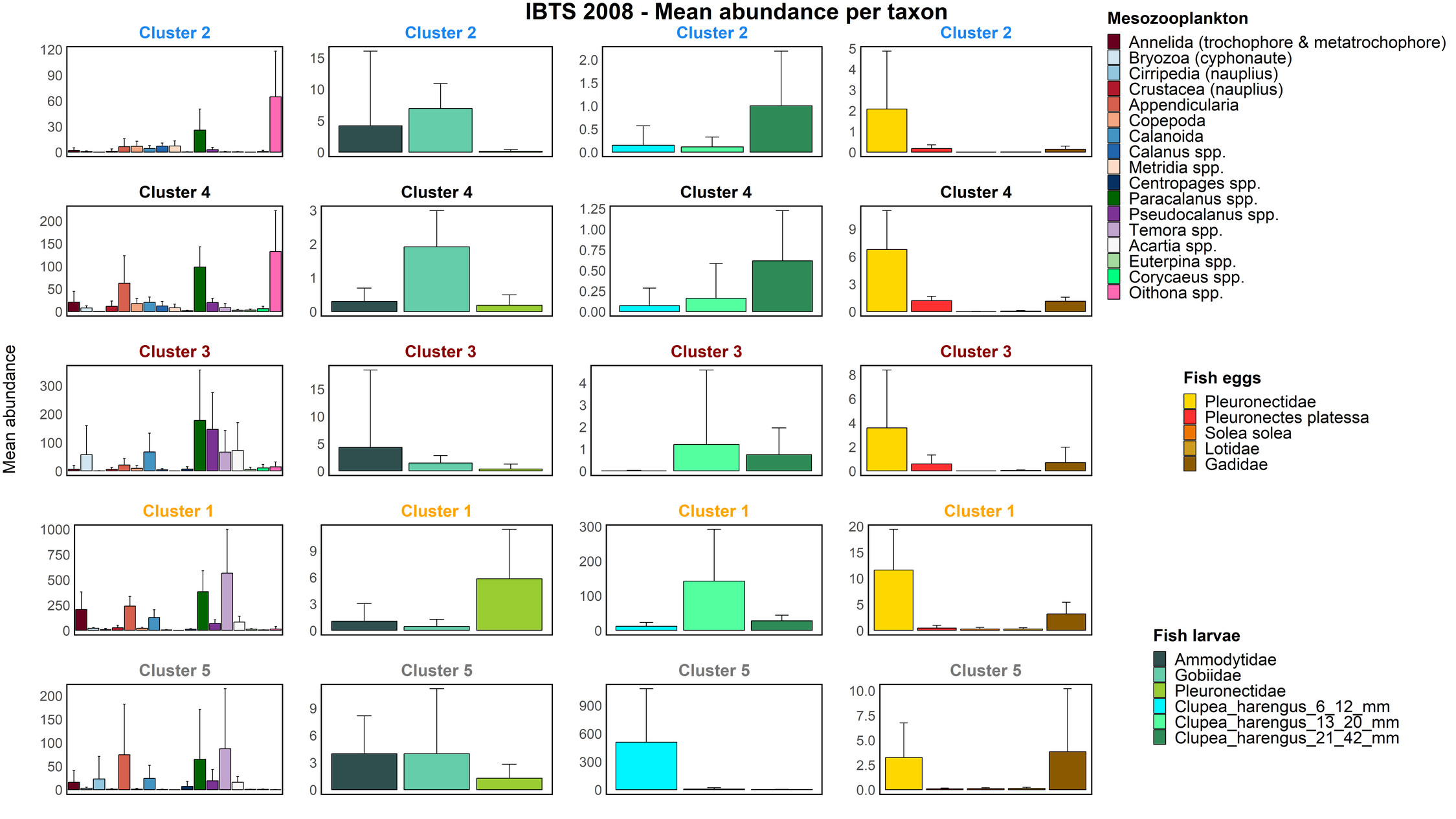

Supplement: S12 Fig — Mean abundance is therefore not comparable between clusters but gives further inside in community per cluster. (TIF) [file pone.0308803.s012.tif]

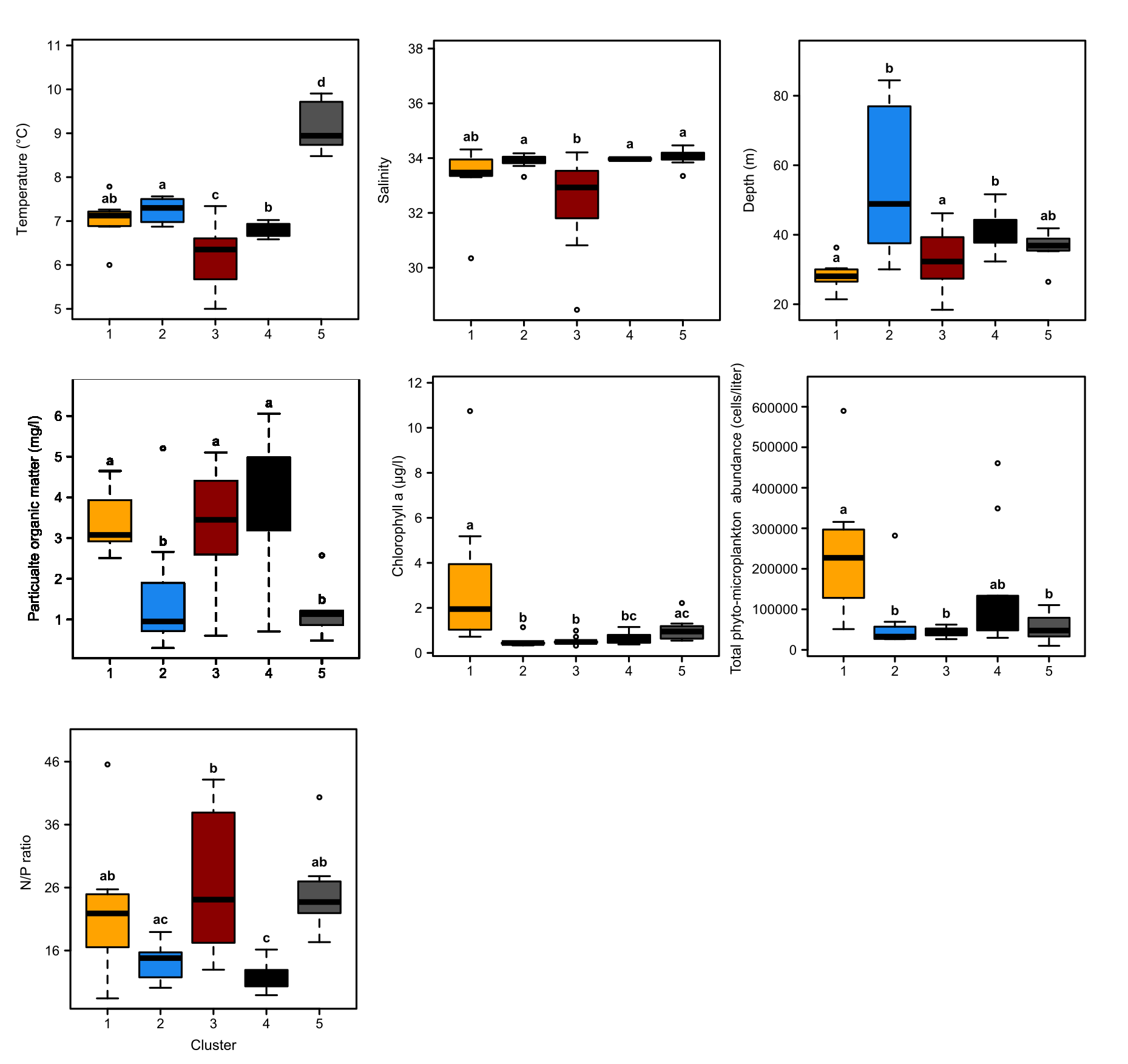

Supplement: S13 Fig — Boxplots displaying the variability of a potential driver per assemblage. Beginning in the upper left corner continuing to the right: temperature, salinity, depth, concentration of particulate organic matter, concentration of chlorophyll a, total phyto- and microplankton abundance and Nitrogen/Phosphorus ratio. (TIF) [file pone.0308803.s013.tif]

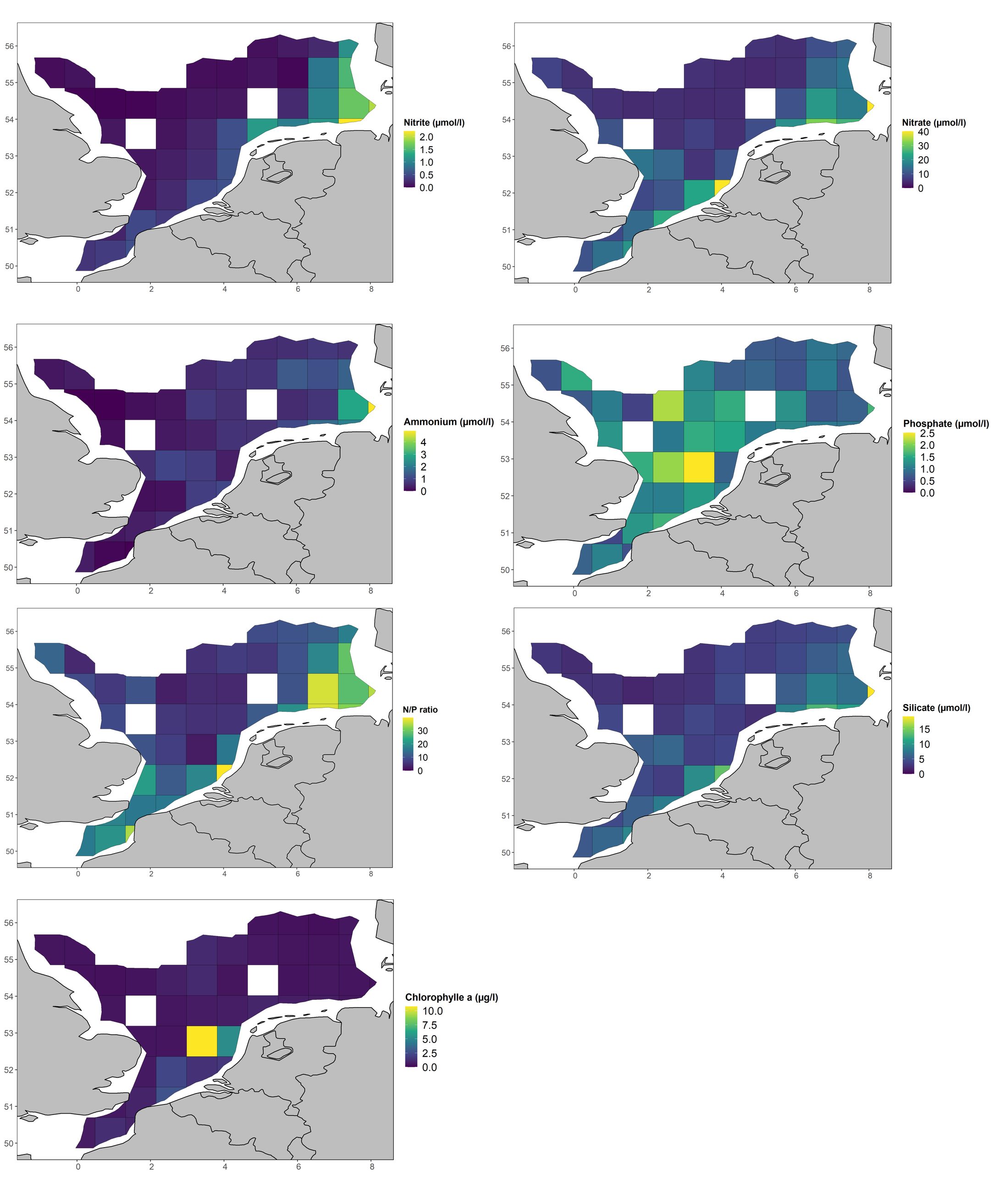

Supplement: S14 Fig — (TIF) [file pone.0308803.s014.tif]

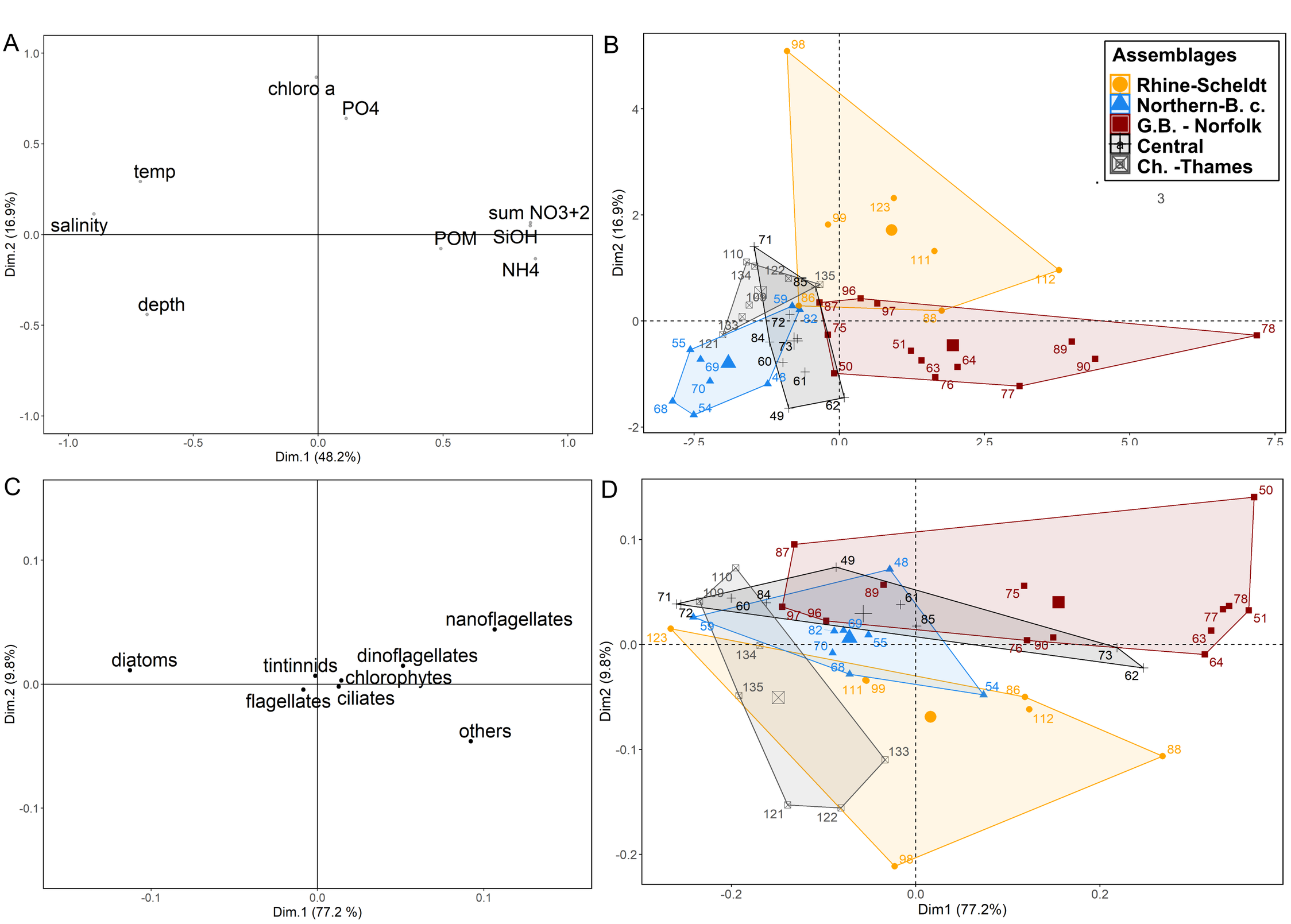

Supplement: S15 Fig — (A) Abiotic parameters displayed in a two-dimensional space of a PCA. (B) Assemblages/cluster displayed in the same two-dimensional space as in A. Numbers indicate grid cell ID. (C) Phyto- and microplankton groups displayed in a two-dimensional space of a PCA. (D) Assemblages/cluster displayed in the same two-dimensional space as in C. Numbers indicate grid cell ID. (TIF) [file pone.0308803.s015.tif]

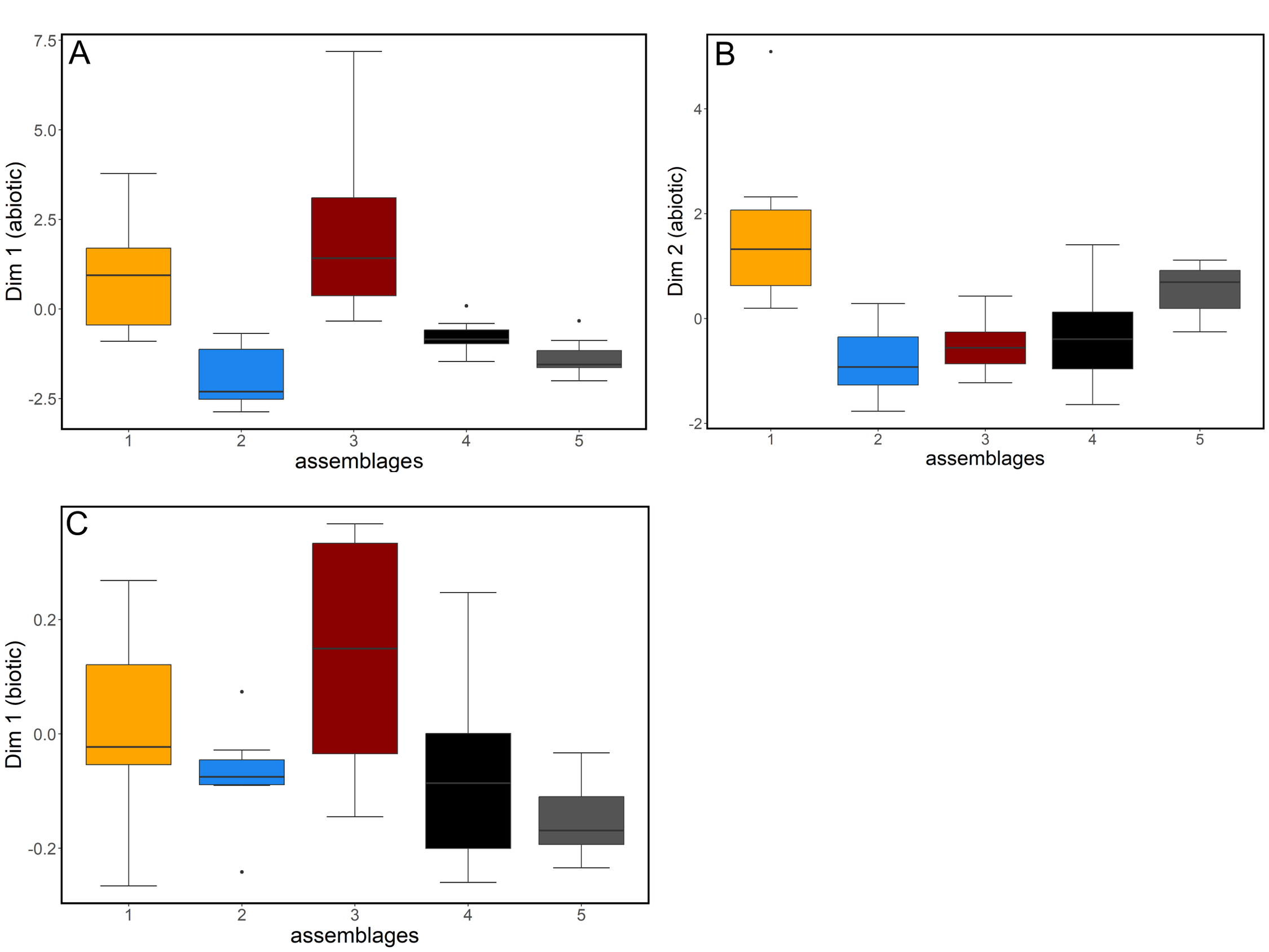

Supplement: S16 Fig — (A) First dimension of PCA applied to abiotic parameters. (B) Second dimension of PCA applied to abiotic parameters. (C) First dimension of PCA applied to biotic parameters. (TIF) [file pone.0308803.s016.tif]

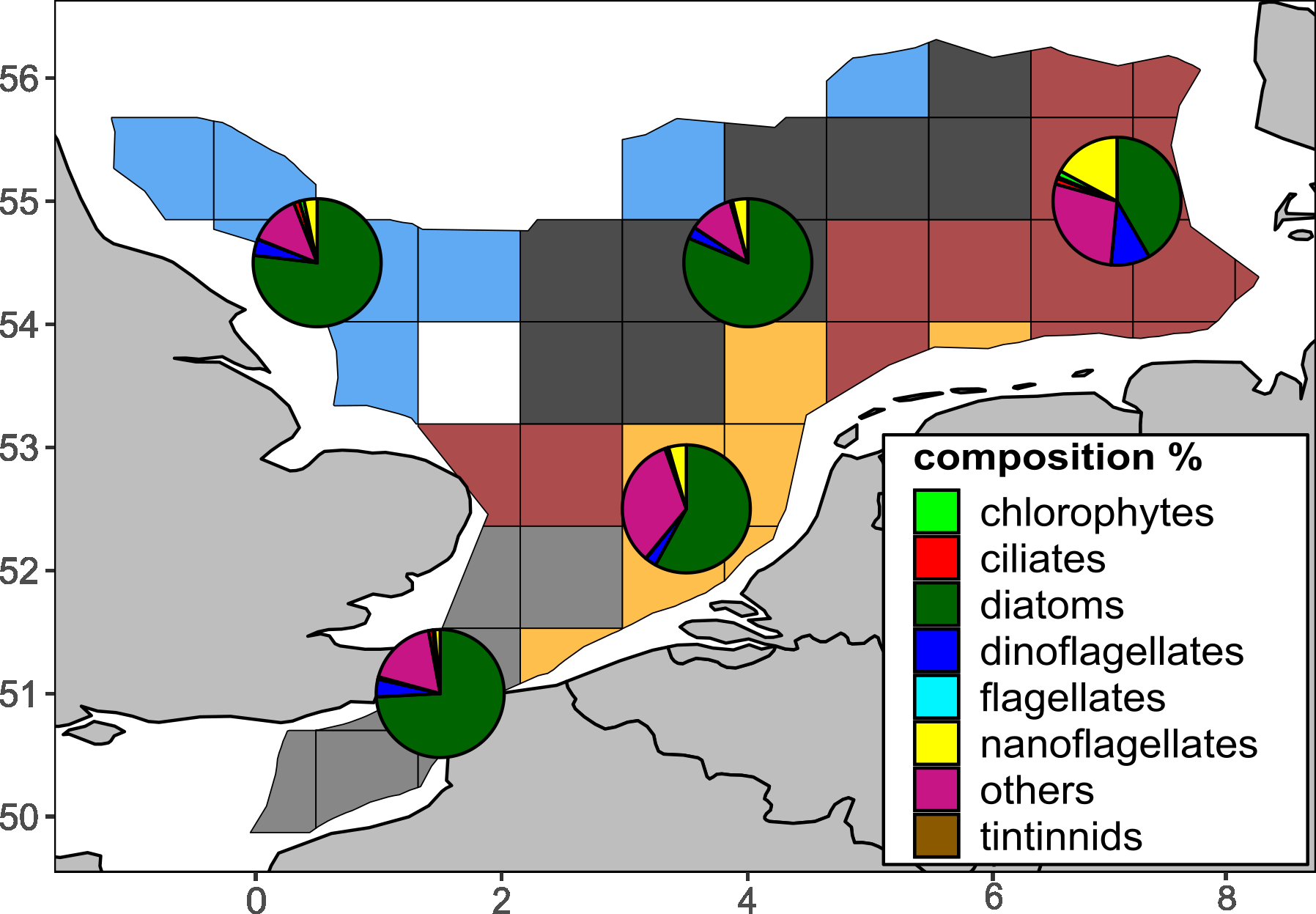

Supplement: S17 Fig — (TIF) [file pone.0308803.s017.tif]

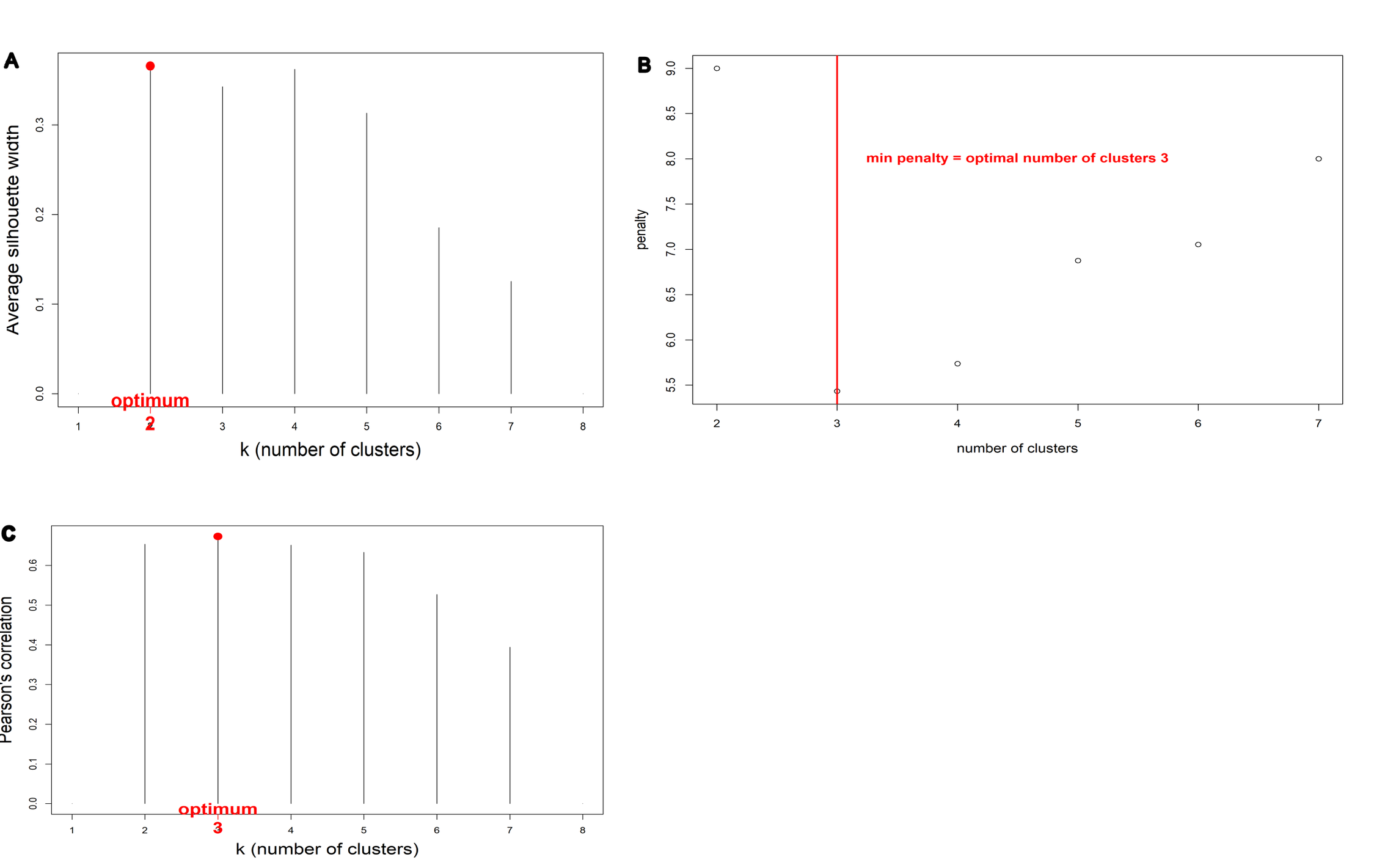

Supplement: S18 Fig — (A) Silhouette width indicating an optimum number of three clusters, (B) Kelly-Gardner-Sutcliffe penalty function proposing an optimal number of four clusters, (C) Mantel correlation indicating an optimal number of four clusters. (TIF) [file pone.0308803.s018.tif]

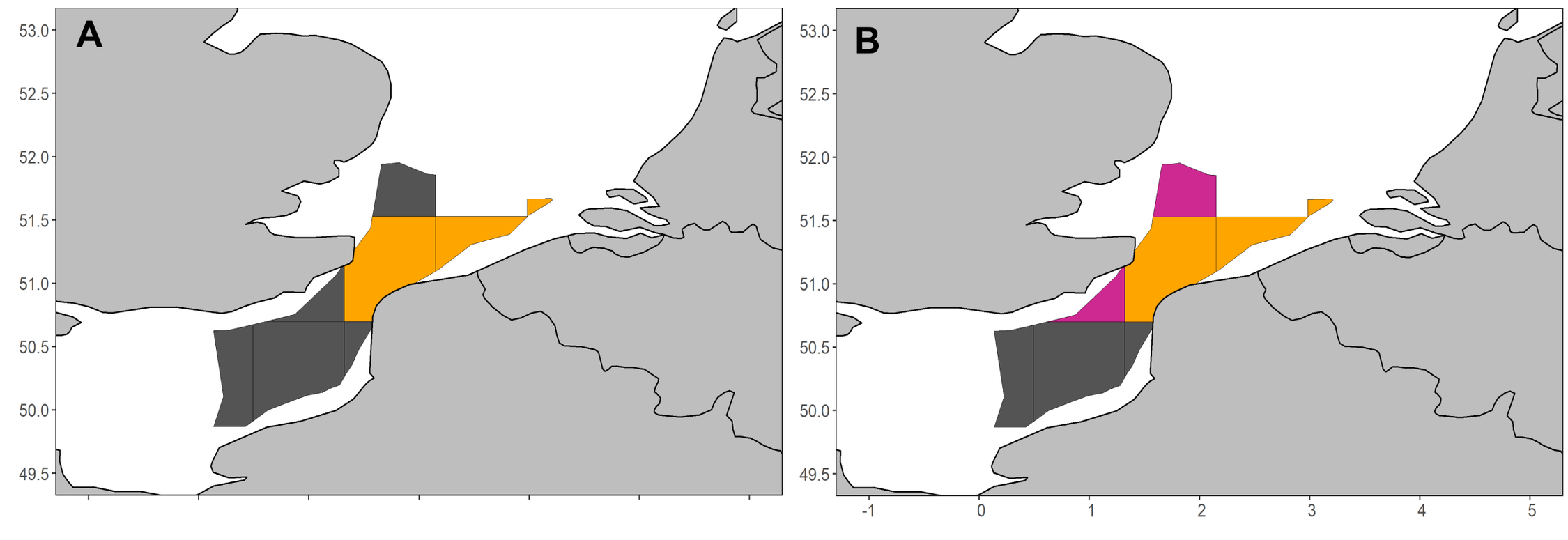

Supplement: S19 Fig — (A) Clusters derived using a k = 2; (B) Clusters derived using a k = 3. (TIF) [file pone.0308803.s019.tif]

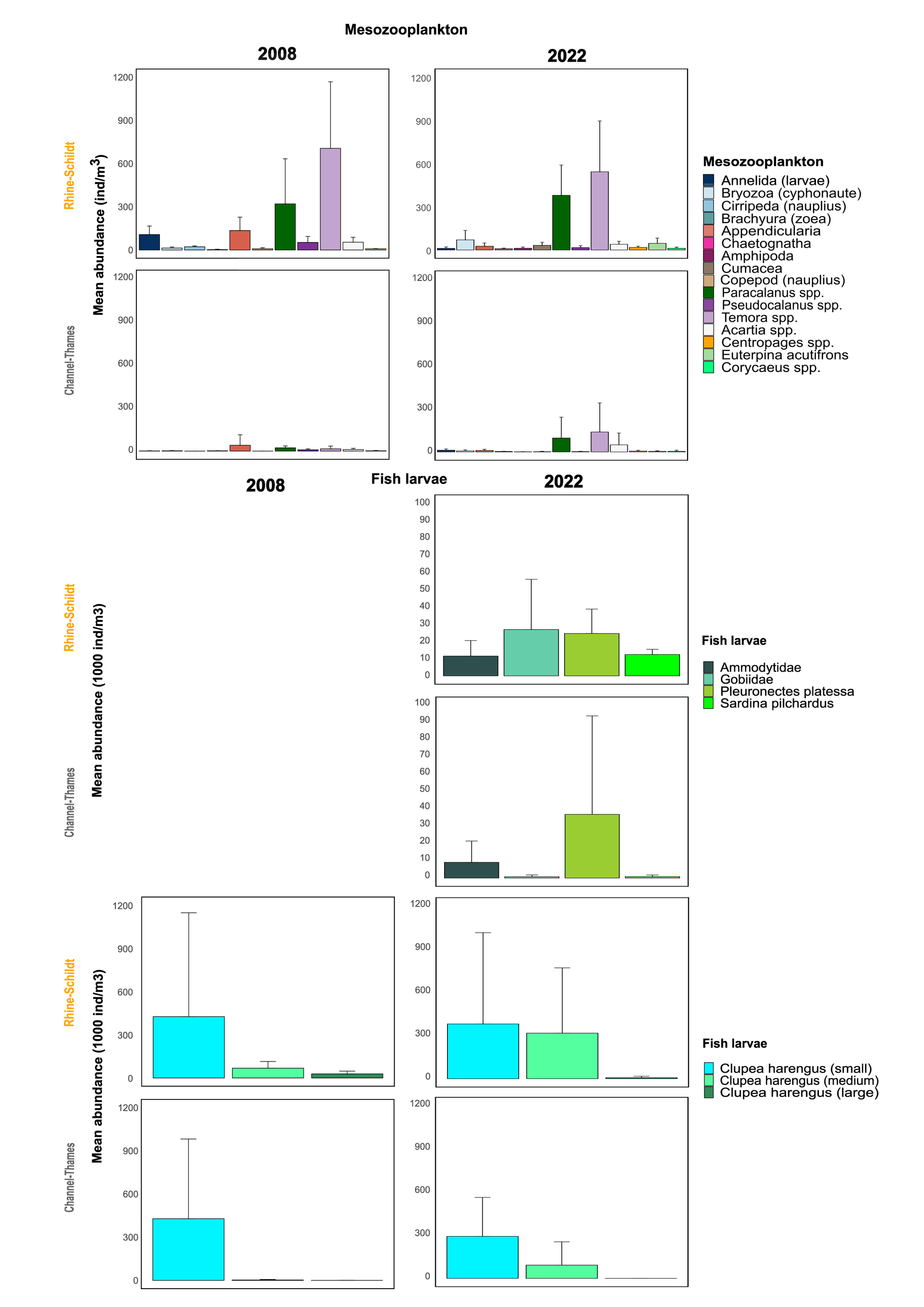

Supplement: S20 Fig — Taxa composition of assemblages in 2008 (left) and 2022 (right). Mean abundance of taxa was calculated with regard to the spatial distribution of clusters in 2008 for both years. (TIF) [file pone.0308803.s020.tif]

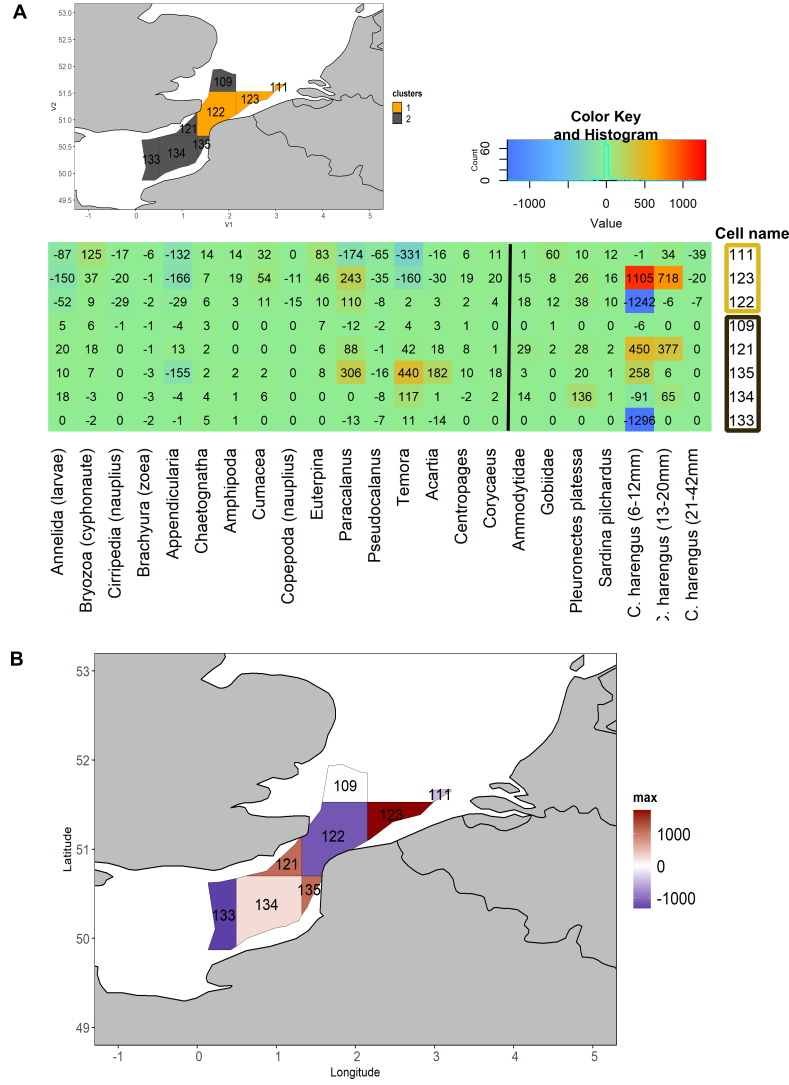

Supplement: S21 Fig — Abundance in 2008 was subtracted form abundance in 2022. A: Heatmap displaying increase or decrease per taxon and cell. B: Difference of total abundance of mesozoo- and ichthyoplankton per grid cell. (TIF) [file pone.0308803.s021.tif]

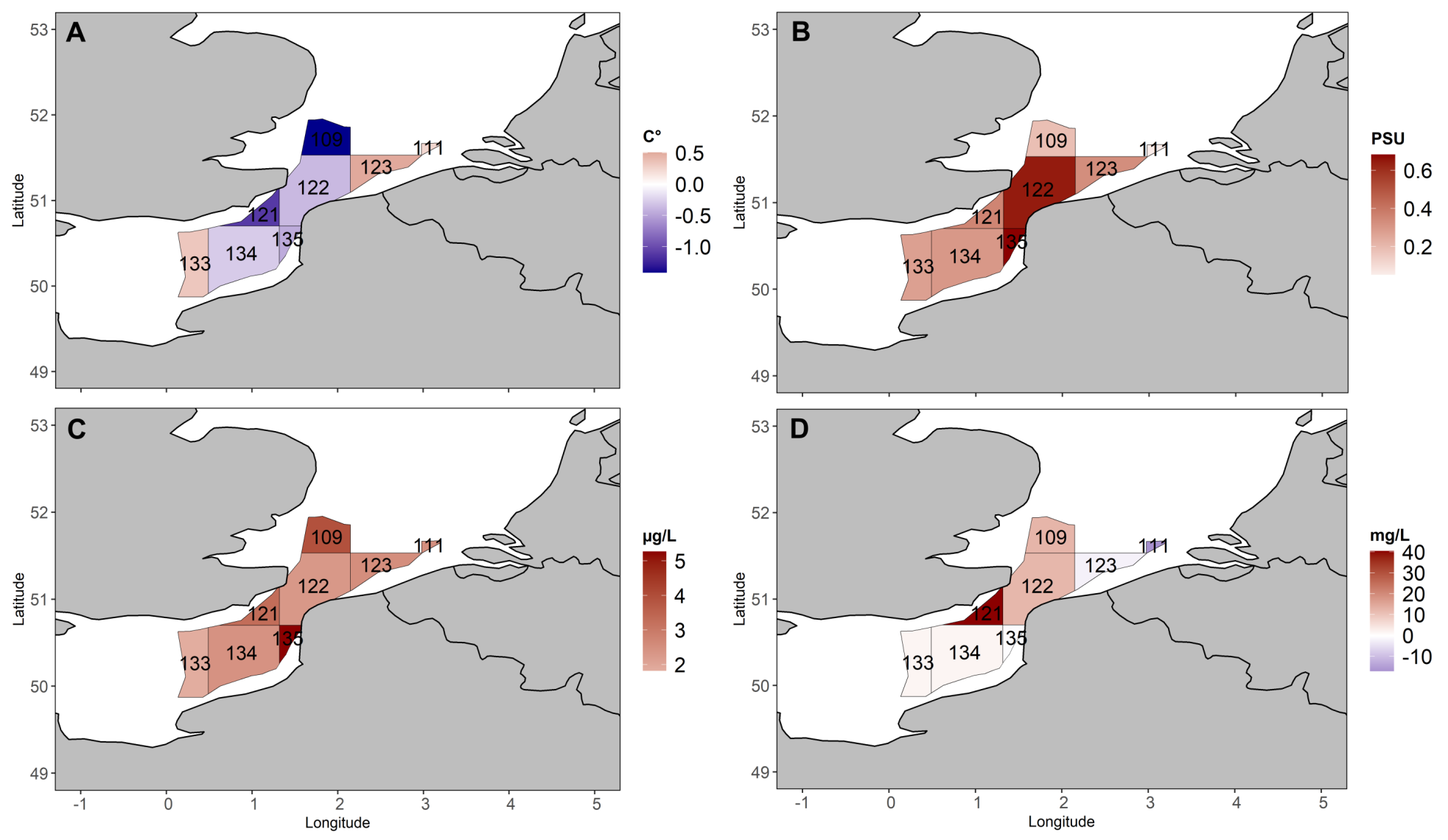

Supplement: S22 Fig — Measurements taken in 2008 were subtracted from measurements taken in 2022 so that positive values indicate an increase in 2022 compared to 2008. Numbers in grid cells indicate grid cell ID. A: Temperature; B: Salinity; C: Chlorophyll a; D: Particulate organic matter. (TIF) [file pone.0308803.s022.tif]
