# Supplementary material for: Winter distribution of zooplankton and ichthyoplankton assemblages in the North Sea and the English Channel
Source: PLoS One. 2024 Oct 7;19(10):e0308803. doi: 10.1371/journal.pone.0308803 (PMC11458026; doi:10.1371/journal.pone.0308803)
Supplement: S1 Table — (DOCX) [file pone.0308803.s023.docx]

| **Mesozooplankton** | **Fish larvae** | **Fish eggs** |  | classification |
| --- | --- | --- | --- | --- |
| Annelida | Ammodytidae | Gadidae |  | Phylum |
| Annelida (trochophore & metatrochophore larvae) | Gobiidae | Pleuronectidae |  | Subphylum |
| Chaetognatha | Pleuronectidae | Lotidae |  | Classe |
| Cnidaria | Syngnathidae | *Pleuronectes platessa* |  | Infraclass |
| Echinodermata | *Trisopterus luscus* | *Solea solea* |  | Subclass |
| Ctenophora | *Liparis liparis* |  |  |  |
| Crustacea | *Clupea harengus* |  |  | Order |
| Crustacea (nauplius larvae) | *Sardina pilchardus* |  |  | Infraorder |
| Crustacea (cyprid larvae) |  |  |  | Suborder |
| Bryozoa (cyphonaute larvae) |  | |  | Family |
| Gasteropoda |  |  |  | Genus |
| Bivalvia |  |  |  | Species |
| Asteroida (brachiolaria larvae) |  |  |  |  |
| Appendicularia |  |  |  |  |
| Cirripeda (nauplius larvae) |  |  |  |  |
| Copepoda |  |  |  |  |
| Copepoda (nauplius) |  |  |  |  |
| Calanoida |  |  |  |  |
| Cyclopoida |  |  |  |  |
| Harpacticoida |  |  |  |  |
| Amphipoda |  |  |  |  |
| Cumacea |  |  |  |  |
| Euphausiacea |  |  |  |  |
| Isopoda |  |  |  |  |
| Mysidacea |  |  |  |  |
| Siphonophora |  |  |  |  |
| Anomura (zoea larvae) |  |  |  |  |
| Brachyura (zoea larvae) |  |  |  |  |
| Brachyura (megalopa larvae) |  |  |  |  |
| Caridea (zoea) |  |  |  |  |
| Cladocera |  |  |  |  |
| Hyperiidae |  |  |  |  |
| *Acartia spp.* |  |  |  |  |
| *Calanus spp.* |  |  |  |  |
| *Candacia spp.* |  |  |  |  |
| *Centropages spp.* |  |  |  |  |
| *Corycaeus spp.* |  |  |  |  |
| *Euterpina spp.* |  |  |  |  |
| *Metridia spp.* |  |  |  |  |
| *Paracalanus spp.* |  |  |  |  |
| *Pseudocalanus spp.* |  |  |  |  |
| *Temora spp.* |  |  |  |  |
| *Clione spp.* |  |  |  |  |
| *Oithona spp.* |  |  |  |  |
| *Tomopteris spp.* |  |  |  |  |
| *Pleurobrachia spp.* |  |  |  |  |
