# Supplementary material for: Winter distribution of zooplankton and ichthyoplankton assemblages in the North Sea and the English Channel
Source: PLoS One. 2024 Oct 7;19(10):e0308803. doi: 10.1371/journal.pone.0308803 (PMC11458026; doi:10.1371/journal.pone.0308803)
Supplement: S2 Table — (DOCX) [file pone.0308803.s024.docx]

| **Mesozooplankton** | **Fish larvae** | classification |
| --- | --- | --- |
| Annelida | Ammodytidae | Phylum |
| Chaetognatha | Syngnathidae | Subphylum |
| Cnidaria | *Pomatoschistus sp* | Classe |
| Cnidaria (ephyra) | *Agonus cataphractus* | Infraclass |
| Annelida (larvae) | *Aphia minuta* | Subclass |
| Echinodermata (larvae) | *Crystallogobius linearis* | Order |
| Crustacea (megalopa) | *Pomatoschistus minutus* | Infraorder |
| Crustacea (nauplii) | *Clupea harengus* | Suborder |
| Bryozoa (cyphonaute) | *Liparis liparis* | Family |
| Bivalvia | *Microstomus kitt* | Genus |
| Mollusca | *Pleuronectes platessa* | Species |
| Ostracoda | *Sardina pilchardus* |  |
| Echinoidea (pluteus) | *Solea solea* |  |
| Ophiuroidea (pluteus) | *Syngnathus acus* |  |
| Cirripedia (cypris) |  |  |
| Cirripedia (naulius) |  |  |
| Copepoda (nauplii) |  |  |
| Amphipoda |  |  |
| Calanoida |  |  |
| Euphausiacea (calyptopis larvae) |  |  |
| Calanoida (copepodite) |  |  |
| Cumacea |  |  |
| Cyclopoida |  |  |
| Decapoda |  |  |
| Euphausiacea |  |  |
| Harpacticoida |  |  |
| Isopoda |  |  |
| Mysidacea |  |  |
| Mysidacea (larvae) |  |  |
| Siphonostomatoida |  |  |
| Decapoda (zoea) |  |  |
| Brachyura (zoea) |  |  |
| Brachyura |  |  |
| Anomura |  |  |
| Peltidiidae |  |  |
| Calanidae |  |  |
| Caprellidae |  |  |
| Caridea |  |  |
| Centropagidae |  |  |
| Calanidae (copepodite) |  |  |
| Gnathiidae |  |  |
| Oithonidae |  |  |
| Paguridae |  |  |
| Porcellanidae |  |  |
| *Acartia spp.* |  |  |
| *Calanus spp.* |  |  |
| *Candacia spp.* |  |  |
| *Centropages spp.* |  |  |
| *Corycaeus spp.* |  |  |
| *Evadne spp.* |  |  |
| *Labidocera spp.* |  |  |
| *Obelia spp.* |  |  |
| *Ophiura spp.* |  |  |
| *Podon spp.* |  |  |
| *Paracanlus.Pseudocalanus* |  |  |
| *Pseudodiaptomus spp.* |  |  |
| *Temora longicornis* |  |  |
| *Euterpina acutifrons* |  |  |
| *Pseudocalanus elongatus* |  |  |
| *Pleurobrachia pileus* |  |  |
| *Paracalanus parvus* |  |  |
| *Oikopleura dioica* |  |  |
