# Supplementary material for: Winter distribution of zooplankton and ichthyoplankton assemblages in the North Sea and the English Channel
Source: PLoS One. 2024 Oct 7;19(10):e0308803. doi: 10.1371/journal.pone.0308803 (PMC11458026; doi:10.1371/journal.pone.0308803)
Supplement: S1 File — (PDF) [file pone.0308803.s025.pdf]

S1 Section: Taxa determination mesozooplankton

Species determination was assisted by a ZooScan [1,2]. Briefly, a ZooScan allows scanning zooplankton samples as pictures, which are subsequently used by a machine-learning algorithm for species determination. The machine-learning algorithm used in 2008 was Plankton Identifier (http://www.obs-vlfr.fr/~gaspari/Plankton_Identifier/index.php) and Eco-Taxa (http://ecotaxoserver.obs-vlfr.fr) in 2022. Both algorithms are based on the ZooProcess software and produce comparable results. Samples stored in 1% formol were rinsed and specimens were divided in two size classes (A: 200-500 µm, B: >500 µm) to increase determination success of the method. To further enhance determination exactness, samples were fractionated by means of the Motoda method [1,3].

References

1. Grosjean P, Picheral M, Warembourg C, Gorsky G. Enumeration, measurement, and identification of net zooplankton samples using the ZOOSCAN digital imaging system. ICES J Mar Sci. 2004 Jan 1;61(4):518–25.

2. Gorsky G, Ohman MD, Picheral M, Gasparini S, Stemmann L, Romagnan JB, et al. Digital zooplankton image analysis using the ZooScan integrated system. J Plankton Res. 2010 Mar 1;32(3):285–303.

3. Motoda S. Devices of simple plankton apparatus. Mem Fac Fish Hokkaido. 1959;7(1–2):73–94.
