# Supplementary material for: Winter distribution of zooplankton and ichthyoplankton assemblages in the North Sea and the English Channel
Source: PLoS One. 2024 Oct 7;19(10):e0308803. doi: 10.1371/journal.pone.0308803 (PMC11458026; doi:10.1371/journal.pone.0308803)
Supplement: S2 File — (PDF) [file pone.0308803.s026.pdf]

S2 Section: Calculation of mesozooplankton abundance

Mesozooplankton abundance per taxon and station/sample was calculated as follows.

The water volume filtered by the WP2 bongonet was derived using equation (1), where *V* is the filtered volume of seawater in m^3, *T* the number of turns of the volume meter, *0.3* the conversion factor provided by the manufacturer of the volume meter and *D* the net diameter (D=0.57 m).

$$V = T * 0.3 * \left[\pi * \left(\frac{D}{2}\right)^2\right] \qquad (1)$$

The abundance per taxon and station (*Ab* in individuals per m^3) was calculated using equation (2), with *N* being the number of individuals identified per size class (A and B), *F* the fraction of the sample after application of the Motoda method (e.g. $\frac{1}{16}$) and *V* the volume filtered per station / sample.

$$Ab = \frac{\left(\frac{NA}{FA} + \frac{NB}{FB}\right)}{V} \qquad (2)$$
