# Supplementary material for: Winter distribution of zooplankton and ichthyoplankton assemblages in the North Sea and the English Channel
Source: PLoS One. 2024 Oct 7;19(10):e0308803. doi: 10.1371/journal.pone.0308803 (PMC11458026; doi:10.1371/journal.pone.0308803)
Supplement: S3 File — (PDF) [file pone.0308803.s027.pdf]

S3 Section: Calculation of fish larvae and fish eggs, phyto-microplankton

The water volume filtered by the sampling device was derived as described under S2 Section. The abundance per taxon and station (in individuals per m^3 for fish eggs, in individuals per 1000 m^3 for fish larvae, in cells per liter for phyto-microplankton) was calculated by dividing the number of individuals per station by the volume filtered.
