# Supplementary material for: Winter distribution of zooplankton and ichthyoplankton assemblages in the North Sea and the English Channel
Source: PLoS One. 2024 Oct 7;19(10):e0308803. doi: 10.1371/journal.pone.0308803 (PMC11458026; doi:10.1371/journal.pone.0308803)
Supplement: S4 File — (PDF) [file pone.0308803.s028.pdf]

S4 Section: Determination optimal grid cell size

The optimal grid cell size was determined using data from 2008 exclusively.

Mesozooplankton, fish larvae and fish egg datasets differed in their sampling extent as in their sampling resolution. To facilitate the combined analysis of all datasets, a common sampling area and grid had to be defined.

The area of analysis (polygon) was restricted to the sampling extent of the fish egg-dataset as the dataset covering the smallest sampling area. This facilitated availability of samples of all datasets in the entire study area. Sampling stations of mesozooplankton and fish larvae outside the polygon were excluded from analysis. Within the polygon 141 mesozooplankton samples, 129 fish larvae and 861 fish egg samples were collected.

As the ecoregions defined in subsequent analysis will be based on a grid dividing the study area in quadrats, we first calculated the optimal grid cell size (Lopt) building on an approach initially applied in agronomics [1]. This approach aims at reducing the undesirable nugget variance (derived from a semi-variogram) as much as possible, whilst minimizing the resulting decrease in informative variance of the spatial structure [1,2].

For this purpose, Tisseyre et al. [1] defined Lopt as the cell length, which optimizes the structural information in a grid cell, by maximizing the sum of two components. The first component is the proportion of nugget variance that is removed (PNR). The second component is the proportion of sill variance that is retained (PS). This relationship was specified in formula 3, where *fv* is the amount of information per grid cell of size v (area), P_{NR} is the proportion of initial noise that is removed and P_S is the proportion of remaining spatially structured variance.

$$fv = PNR + Ps \qquad (3)$$

Tisseyre et al. [1] showed that, with an exponential variogram model, equation (3) could be simplified as a function of the length of the raster cell (*L*), the range (*a*) estimated from the variogram model, and the sampling rate (*r*) in the study area (equation (4)).

$$fv = exp\left(-\frac{L}{2a}\right) + 1 - \frac{1}{rL^2} \qquad (4)$$

To calculate Lopt, we calculated the first derivative of fv relative to L (fv'), and numerically sought the value of L for which fv' was null:

$$fv' = -\frac{1}{2a} * exp\left(-\frac{L}{2a}\right) + \frac{2}{rL^3} \qquad (5)$$

As the sampling resolution of the mesozooplankton and fish larvae datasets (n=141, n=129, respectively) was much lower than that of the fish egg dataset (n=861), only the taxa of the former two datasets were considered to derive Lopt. To solve equation $f_V' = 0$, the range (*a*) of all mesozooplankton and fish larvae groups with a higher occurrence than 10 % and contributing to 95 % of cumulative frequency of abundance in the polygon were calculated. Non-stationarity data (variogram not displaying sill) with a spatial trend accounting for more than 20% ($R^2 >= 0.2$) of the variation were detrended using the least-square regression method. The trend was modelled by fitting a linear or quadratic regression to the spatial coordinates. If the regression model was significant with a R^2 higher than 0.2 the empirical variogram was calculated on the residuals [3]. In case linear and quadratic regression models were significant with a similar R^2, quadratic residuals were chosen for geostatistical analysis (S2 Fig). Finally, it was not possible to fit an exponential variogram model for certain taxa, owing to the spatial structure of their distribution. In case an exponential variogram model could not be fitted to the empirical variogram (8 taxa from 22), the respective taxonomic group was excluded from the grid cell size definition process. In a next step, f_V' was solved for all taxonomic groups with a nugget effect higher than zero (7 taxa from 14) using an evolutionary algorithm running 500 iterations testing 1000 numbers per cycle. Finally, it was verified that the received taxon specific Lopts were bigger than the minimum distance between stations and smaller than the practical range [1].

After having defined the taxon specific Lopt (S3 Fig), we sought a compromise value for which fv was close to the maximum taxon specific fv of all taxa, and for which the number of empty grid cells was kept to a minimum. In other words, the raster cell size chosen should result in a minimum of empty grid cells to achieve a maximum of cells containing information and be as close as possible to the taxa specific Lopts. The avoidance of empty cells with a central position was prioritized before the avoidance of empty cells situated at the margins.

We calculated the across-taxa median of the Lopt specific to each taxon. That median value resulted in a single cell of central position not containing sampling stations and was thus accepted as global Lopt.

All analyses were performed in R version 4.2.1 using the package gstat and sp for the geostatistical analyses and the package raster for definition of empty cells per grid cell size.

<u>References:</u>

1. Tisseyre B, Leroux C, Pichon L, Geraudie V, Sari T. How to define the optimal grid size to map high resolution spatial data? Precis Agric. 2018 Oct;19(5):957–71.

2. Bellehumeur C, Legendre P, Marcotte D. Variance and spatial scales in a tropical rain forest: changing the size of sampling units. Plant Ecol. 1997;130:89–98.

3. Loots C, Vaz S, Planque B, Koubbi P. What controls the spatial distribution of the North Sea plaice spawning population? Confronting ecological hypotheses through a model selection framework. ICES J Mar Sci. 2010 Mar 1;67(2):244–57.
