# Supplementary material for: Winter distribution of zooplankton and ichthyoplankton assemblages in the North Sea and the English Channel
Source: PLoS One. 2024 Oct 7;19(10):e0308803. doi: 10.1371/journal.pone.0308803 (PMC11458026; doi:10.1371/journal.pone.0308803)
Supplement: S5 File — (PDF) [file pone.0308803.s029.pdf]

S5 Section: Choice of taxa for clustering

The zooplankton taxonomic groups Copepoda, Calanoida, and Crustacea nauplius, identified by the species identification software, were excluded from the clustering analysis to assure that each taxon only contributed once to the clustering analysis and to keep the finest taxonomic resolution possible. The mentioned groups regroup taxa that are building a taxonomic group on their own in our data set. An example is the genus *Temora,* which is sorted by the software as proper group but individuals of this genus can also be found in the groups Copepoda and Calanoida.
