# Supplementary material for: Winter distribution of zooplankton and ichthyoplankton assemblages in the North Sea and the English Channel
Source: PLoS One. 2024 Oct 7;19(10):e0308803. doi: 10.1371/journal.pone.0308803 (PMC11458026; doi:10.1371/journal.pone.0308803)
Supplement: S6 File — (PDF) [file pone.0308803.s030.pdf]

S6 Section: Pilot study clustering method

In a pilot study (S5 Fig) two hierarchical and one fuzzy clustering method [1] were applied and the output mapped to get insight into spatial patterns of the datasets and to evaluate the most appropriate clustering algorithm.

Hierarchical clustering can be applied using different clustering algorithms. The algorithm representing the original distance matrix most adequately should be used [1]. To determine the most appropriate algorithm, eight different clustering algorithms (unweighted pair-group method using arithmetic averages, unweighted pair-group method using centroids, weighted pair-group method using arithmetic averages, weighted pair-group method using centroids, Ward's method, single linkage, complete linkage, divisive hierarchical method) were tested [2] using Shepard-like diagrams. These diagrams display the cophenetic correlation [1] between the cophenetic matrix produced by the algorithm and the original distance matrix. The algorithm with the highest cophenetic correlation was chosen for clustering and for the determination of the optimal number of clusters.

Hierarchical clustering was applied on principal components of a PCA using all dominant taxa and directly implemented on the data using the unweighted pair-group method with arithmetic averages (average linkage). The output of the fuzzy clustering was chosen as the most appropriate approach and used for further analysis.

References:

1. Borcard D, Gillet F, Legendre P. Numerical ecology with R. Vol. 2. Springer; 2011.
2. Hattab T, Albouy C, Ben Rais Lasram F, Le Loc'h F, Guilhaumon F, Leprieur F. A biogeographical regionalization of coastal Mediterranean fishes. J Biogeogr. 2015 Jul;42(7):1336–48.
