# Supplementary material for: Winter distribution of zooplankton and ichthyoplankton assemblages in the North Sea and the English Channel
Source: PLoS One. 2024 Oct 7;19(10):e0308803. doi: 10.1371/journal.pone.0308803 (PMC11458026; doi:10.1371/journal.pone.0308803)
Supplement: S7 File — (PDF) [file pone.0308803.s031.pdf]

S7 Section: Definition of optimal number of clusters

As mentioned above clustering methods require an *a priori* definition of the number of clusters (k) or the cut-off level [1]. To make this choice more objective, Kreft and Jetz [1] proposed the use of statistical methods for the definition of the optimal k. Using three different methods namely the Silhouette widths, the Mantel correlation between the distance matrix and binary matrix computed from the dendrogram [2] and the Kelly-Gardner-Sutcliffe penalty function [3] on clustering results achieved with the previously as most appropriately defined clustering algorithm resulted in different optimal k. To evaluate the k most appropriate to the data set, clustering was conducted using all statistically derived k. The resulting clusters were mapped and their ecological meaning evaluated assessing the existence of indicator species and considering the spatial coherence of the clusters (membership value and spatial distribution). Indicator taxa were determined by means of the IndVal method [4], which reveals taxa with a high fidelity and specificity for a cluster. Cluster-specific taxa display a high abundance in a cluster compared to the others. Taxa with a high fidelity for a cluster occur in a high number of grid cells belonging to that cluster. All indicator taxa considered, had a higher indicator value than 0.25 [4,5]. Analysis was conducted using the package labdsv and a p-value of 0.05 adjusted after Benjamini and Hochberg [6].

Using the average silhouette width, the Kelly-Gardner-Sutcliffe penalty function and the Mantel correlation, k values of 4 and 3 were found (S6 Fig). Restricting clustering to a number of 3 produced a continuous region of lower membership values towards the center of the study area (S7 Fig). As this region of lower membership values turned into a cluster when allowing for 4 clusters (S8 and S7B Figs), setting k to 4 was judged more appropriate to describe zooplankton assemblages present in the study area than setting k to 3. The blurred pattern of lower membership values resulting from a k of 4 and 5 indicated (S7C Fig), that a k higher than 5 would not reveal further spatially-coherent clusters. As indicator species were found for clusters one to five (Fig 3B), a distinctively lower mesozooplankton abundance was observed in cluster 5 compared to cluster 1 (Fig 3C) and abundance of herring larvae (6-12 mm) was distinctively higher in cluster 5 compared to cluster 1 (S11 Fig), a k of 5 was chosen as the most appropriate number of clusters.

<u>References:</u>

1. Kreft H, Jetz W. A framework for delineating biogeographical regions based on species distributions: Global quantitative biogeographical regionalizations. J Biogeogr. 2010 Nov;37(11):2029–53.

2. Borcard D, Gillet F, Legendre P. Numerical ecology with R. Vol. 2. Springer; 2011.

3. Hattab T, Albouy C, Ben Rais Lasram F, Le Loc'h F, Guilhaumon F, Leprieur F. A biogeographical regionalization of coastal Mediterranean fishes. J Biogeogr. 2015 Jul;42(7):1336–48.

4. Dufrêne M, Legendre P. Species assemblages and indicator species: The need for a felxible asymmetrical appeoach. Ecol Monogr. 1997 Aug;67(3):345–66.

5. Souissi S, Ibanez F, Hamadou RB, Boucher J, Cathelineau AC, Blanchard F, et al. A new multivariate mapping method for studying species assemblages and their habitats: Example using bottom trawl surveys in the Bay of Biscay (France). Sarsia. 2001 Dec 28;86(6):527–42.

6. Benjamini Y, Hochberg Y. Controlling the false discovery rate: a practical and powerful approach to multiple testing. J R Stat Soc Ser B Methodol. 1995;57(1):289–300.
