# Supplementary material for: Winter distribution of zooplankton and ichthyoplankton assemblages in the North Sea and the English Channel
Source: PLoS One. 2024 Oct 7;19(10):e0308803. doi: 10.1371/journal.pone.0308803 (PMC11458026; doi:10.1371/journal.pone.0308803)
Supplement: S8 File — (PDF) [file pone.0308803.s032.pdf]

S8 Section: Detailed characterization of assemblages.

The mesozooplankton of the **Channel-Thames assemblage** was dominated by *Temora spp*. (87 ind/m^3, 29 %), Appendicularia (74 ind/m^3, 24 %) and *Paracalanus spp*. (65 ind/m^3, 21%) (S11 Fig and Fig 4).

The highest mean abundance of the smallest size fraction of herring larvae (6-12mm) was found in this assemblage with a mean abundance of 508 individuals per 1000 m^3 constituting 97 % of the larval assemblage. Furthermore, the mean abundance of Gobiidae larvae was the second highest among clusters (4 ind/1000 m^3, 0.8 %). Abundance of Ammodytidae larvae (4 ind/1000 m^3, 0.7 %) was comparable to the German Bight-Norfolk and the Northern-British coast assemblage.

Eggs of the family Gadidae (4 ind/1000 m^3, 54 %) and Pleuronectidae (1 ind/1000 m^3, 45 %) dominated the egg assemblage.

The indicator taxa for this cluster were herring larvae (6 and 12 mm), Cirripedia nauplius larvae, zoea larvae of the infra-order Anomura, larvae of *Sardina pilchardus* and *Trisopterus luscus* (Fig 3B).

The Channel-Thames region was characterized by negative values of dimension 1 of the abiotic PCA (A1) meaning warmer temperature, average salinity, low nitrogen, silicate and POM concentration and average values on dimension 2 (A2) i.e. phosphate and chlorophyll concentration (Fig 2). The phyto- and microplankton community were characterized by diatoms that accounted for 74% of total abundance (negative values on dimension 2 of the biotic PCA (B1)) (S17 Fig and Fig 2).

The **Rhine-Scheldt assemblage** was characterized by the highest mean abundance of zooplankton (mesozooplankton and ichthyoplankton) (S11 Fig and Fig 3C). Mesozooplankton was dominated by the copepod genera *Temora* (566 ind/m^3, 35 %) and *Paracalanus* (381 ind/m^3, 24%), species belonging to the class Appendicularia (239 ind/m^3, 15 %) and trochophore and metatrochophore larvae of the phylum Annelida (205 ind/m^3, 13 %) (Figs 4 and *5*).

The fish larvae assemblage was dominated by herring larvae of size 13-20 mm (141 ind/1000 m^3, 75 %), followed by size fraction 21-42 mm (27 ind/1000 m^3, 14 %) and 6-12 mm (12 ind/1000 m^3, 6 %). Larvae of the family Pleuronectidae displayed the highest mean abundance among clusters (6 ind/1000 m^3, 3 %).

Displaying the highest and second highest mean abundance among assemblages, eggs of Pleuronectidae (12 ind/m^3, 76 %) and Gadidae (3 ind/m^3, 20 %) dominated the egg assemblage (S11 Fig and Fig 4).

The Rhine-Scheldt assemblage was characterized by 15 indicator species which are enumerated in the following (Fig 3B): *Clupea harengus* size class 13-20 mm, *Clupea harengus* size class 21-42 mm, trochophore and metatrochophore larvae of the phylum Annelida, Annelida, *Tempora spp*., Pleuronectidae larvae, Appendicularia, *Solea solea* eggs, Crustacea nauplii, *Euterpina spp*., Lotidae eggs, Calanoida, *Paracalanus spp*. and Pleuronectidae eggs (Fig 3B). The Rhine-Scheldt region covered the region with highest total zooplankton abundance (Fig 3C). It was characterized by elevated abundance

of phyto- and microplankton (Fig 2F) corresponding positive to values on A2 representing elevated chlorophyll a concentration (Fig 2C). The phyto-microplankton assemblage was dominated by diatoms (58%) and the group of others (33%) (average value on B1) (S17 Fig and Fig 2E). Positive values on A1 represented elevated concentration of POM, nitrogen, phosphate and silicate and average temperature, salinity and depth (S16 Fig). The N/P ratio of 17±12 indicated rather balanced nutrient availability with regard to the Redfield ratio (S14 Fig).

The **German Bight-Norfolk assemblage** was dominated by the copepod genera *Paracalanus* (177 ind/m^3, 30 %), *Pseudocalanus* (146 ind/m^3, 25 %), *Acartia* (71 ind/m^3, 12 %), *Temora* (65 ind/m^3, 11 %) and cyphonaute larvae of the phylum Bryozoa (57 ind/m^3, 10 %). The latter displayed the highest contribution to the community among clusters (S11 Fig and Fig 4).
Larvae of the family Ammodytidae dominated the larvae assemblage (4 ind/1000 m^3, 54 %). Herring larvae abundance was low with herring larvae of 13 to 20 mm displaying a mean abundance of 1 individual per 1000 m^3 (contribution 15 %).
The fish egg assemblage was dominated by Pleuronectidae eggs with 4 individuals per m^3 (73 %).
Fish larvae of the family Syngnathidae (0.66), cyphonaute larvae of the phylum Bryozoa (0.64) and the copepod genus *Pseudocalanus* (0.57) were the indicator taxa for this cluster (Fig 3B). All in all, the east of the German Bight-Norfolk assemblage covered a region of elevated zooplankton abundance (Fig 3C). The German Bight-Norfolk region was characterized by positive values on A1 meaning cold temperature, shallow depth and low salinity as well as elevated concentrations of POM, nitrogen and silicate. Negative values on A2 represented low concentration of phosphate and chlorophyll (Fig 2). A low concentration of phosphate (S14 Fig) and an elevated concentration of nitrogen resulted in an elevated N/P ratio of 21 ±11 (S14 Fig). The phyto- microplankton was dominated by diatoms (42%), others (28%) and nanoflagellates (17%) (positive values on B1) whereby nanoflagellates and dinoflagellates (10%) displayed the highest chair among assemblages (S17 Fig and Fig 2).

The **Central assemblage** was dominated by the copepod genera *Oithona* and *Paracalanus* (132 and 97 ind/m^3, representing 34 and 25 %, respectively) and Appendicularians (62 ind/m^3, 15 %).
The mean abundance of fish larvae was low in the Central assemblage. The egg assemblage was dominated by Pleuronectidae eggs (7 ind/m^3, 75 %), followed by eggs of *Pleuronectes platessa* (1 ind/m^3) and gadoid species (1 ind/m^3) (S11 Fig and Fig 4).
Ten indicator taxa characterized the Central region: Cyclopoida, *Oithona spp*., *Mitridia spp*., Hyperiidae, Cnidaria, *Calanus spp*., Bivalvia, Gasteropoda and Chaetognatha, and one being *Pleuronectes platessa* eggs (Fig 3B).

The Central region displayed average depth, salinity, temperature, silicate and POM concentration (values around zero on A1). Values around zero on A2 mean average chlorophyll a and phosphate concentration (Fig 2). Nitrogen was low resulting in an N/P ratio of 6±3 (S14 Fig and Fig 2). Total phyto-microplankton abundance was elevated (Fig 2F) and dominated by diatoms (81%) (negative value on B1) (S17 Fig and Fig 2).

The dominant taxa of the **Northern-British coast assemblage** were the copepod genera *Oithona* and *Paracalanus* with 65 and 26 individuals per m^3, contributing with 54 and 22 % to the total abundance of the taxa, respectively. Compared to the other clusters, the Northern-British coast assemblage displayed the second highest mean abundance of *Calanus* (7 ind/m^3, 6 %) and *Mitridia* spp. (7 ind/m^3, 6 %) (S11 Fig and Fig 4).

Concerning ichthyoplankton, the Northern-British coast assemblage was dominated by Gobiidae larvae (7 ind/1000 m^3, 55 %) that displayed a similar abudance as in the Channel-Thames assemblage. Mean abundance of herring larvae of all size classes were equal to or lower than one individual per 1000 m^3 in this assemblage representing a maximum contribution of 8 % to the larval assemblage.

The fish egg assemblage was dominated by Pleuronectidae eggs (2 ind/m^3, 87 %), which were found in similar abundance in the German Bight and the Channel-Thames region.

Two indicator taxa were revealed for the Northern-British coast region, namely the order Euphausiacea and Gobiidae larvae (Fig 3B).

With regard to environmental conditions, this region was characterized by deep depth, elevated salinity and warm temperature and low concentrations of POM, nitrogen, silicate (negative values on A1) and phyto-microplankton abundance (Fig 2). Negative values on A2 mean low chlorophyll a concentration (S14 Fig). N/P ratio was low (8±3). Diatoms dominated the phyto-microplankton assemblage as indicated by negative values on B1.
