# Supplementary material for: Winter distribution of zooplankton and ichthyoplankton assemblages in the North Sea and the English Channel
Source: PLoS One. 2024 Oct 7;19(10):e0308803. doi: 10.1371/journal.pone.0308803 (PMC11458026; doi:10.1371/journal.pone.0308803)
Supplement: S9 File — (PDF) [file pone.0308803.s033.pdf]

S9 Section: Maps and heatmaps of inter-annual differences of taxa abundance and environmental parameters per grid cell

To further evaluate the inter-annual differences and similarities in the zooplankton assemblages revealed by the GLMM, the difference in abundance was calculated per cell and was mapped and displayed as heatmap (S21 Fig A).

As can be seen in S21 Fig the abundance of mesozooplankton taxa per cell and per region did not display a great variability, which was in accordance with the output of the GLMM. The significant decrease of Appendicularia in the Rhein-Scheldt region was mostly due to a decrease in the northern cells 111 and 123 (S21 Fig A).

The significantly higher overall larvae abundance in 2022 was influenced by plaice larvae, that had an abundance increase of 136 individuals in cell 134.

Elevated variability in abundance per cell could be detected for small and medium sized herring larvae. The decreases and increases between the cells per clusters did equilibrate each other, however, so that in the overall assemblage the abundance of herring larvae remained rather stable, which was in accordance with the output of the GLMM.

The differences in total abundance (mesozooplankton and ichthyoplankton) was very variable from cell to cell (S21 Fig B) not revealing further trends in interannual differences between 2008 and 2022.

Also cell specific interannual variability of environmental parameters per cell (S22 Fig) did not reveal clear patterns and remained small so that further hypothesis about potential environmental drivers of the abundance differences found remain difficult.

In conclusion, the interannual comparison between 2008 and 2022 presented in the manuscript and above could show that overall community composition remained stable despite a decrease in Appendicularians and increase in fish larvae other than herring.

More sophisticated analyses such as a Generalized Additive Mixed Models were attempted, but failed (lack of statistical power and/or convergence) due to restricted number of datapoints (3 to 5 cells per cluster, around 43 sampling stations), parameters and spatial cover available in both years.
